# Supplementary material for: Bias-invariant RNA-sequencing metadata annotation
Source: Gigascience. 2021 Sep 22;10(9):giab064. doi: 10.1093/gigascience/giab064 (PMC8559615; doi:10.1093/gigascience/giab064)
Supplement: giab064_GIGA-D-20-00367_Revision_1 [file giab064_giga-d-20-00367_revision_1.pdf]

GigaScience

Bias invariant RNA-seq metadata annotation

--Manuscript Draft--

**Manuscript Number:**	GIGA-D-20-00367R1
**Full Title:**	Bias invariant RNA-seq metadata annotation
**Article Type:**	Technical Note
**Funding Information:**	Deutsche Forschungsgemeinschaft (CRU 306 P-C) — Mr. Sven Heins<br>Deutsche Forschungsgemeinschaft (296 P8) — Mr. Hannes Wartmann<br>Deutsche Forschungsgemeinschaft (CRC 1286 Z2) — Prof. Dr. Stefan Bonn
**Abstract:**	Background : Recent technological advances have resulted in an unprecedented increase in publicly available biomedical data, yet the reuse of the data is often precluded by experimental bias and a lack of annotation depth and consistency. Missing annotations makes it impossible for researchers to find datasets specific to their needs. Findings : Here we investigate RNA-seq metadata prediction based on gene expression values. We present a deep-learning based domain adaptation algorithm for the automatic annotation of RNA-seq metadata. We show how our algorithm outperforms existing linear regression based approaches as well as traditional neural network methods for the prediction of tissue, sample source, and patient sex information across several large data repositories. By using a model architecture similar to siamese networks the algorithm is able to learn biases from datasets with few samples. Conclusion : Using our novel domain adaptation approach we achieved metadata annotation accuracies up to 12.3% better than a previously published method. Using the best model we provide a list of more than 10,000 novel tissue and sex label annotations for 8,495 unique SRA samples. Our approach has the potential to revive idle datasets by automated annotation making them more searchable. The source code as well as an example are available at: github.com/imsb-uke/rna_augment
**Corresponding Author:**	Stefan Bonn<br>Zentrum für Molekulare Neurobiologie<br>Hamburg, Bitte wählen Sie Region, Land oder Bundesland GERMANY
**Corresponding Author Secondary Information:**	
**Corresponding Author's Institution:**	Zentrum für Molekulare Neurobiologie
**Corresponding Author's Secondary Institution:**	
**First Author:**	Hannes Wartmann
**First Author Secondary Information:**	
**Order of Authors:**	Hannes Wartmann<br>Sven Heins<br>Karin Kloiber<br>Stefan Bonn
**Order of Authors Secondary Information:**	
**Response to Reviewers:**	Rebuttal of 'Bias invariant RNA-seq metadata annotation'<br><br>The reviewers raised a couple of very valid points of critique, especially with respect to potential overfitting of the ANN models. We addressed all concerns and believe that these changes significantly improved the scientific standard of the revised manuscript. In the end, we would like to thank the reviewers for their excellent suggestions, comments, and time.

Powered by Editorial Manager® and ProduXion Manager® from Aries Systems Corporation

In the PDF version, uploaded with the manuscript as "answers to reviewers" and tagged as "Supplementary Material", reviewer questions are in black font, our answers in blue font and text changes to the manuscript in black font in italics.

Reviewer reports:

Reviewer #1: In this manuscript, the authors address the task of phenotype prediction from gene expression data, with a focus on gene expression profiles measured via RNA-seq and the phenotypes of tissue, sample source (tissue biopsy or cell line culture), and sex. The primary motivation for the task is the improvement of metadata labeling RNA-seq samples, particularly in public databases such as the Sequence Read Archive (SRA), for which the metadata are often incomplete and unstandardized. Recently, a linear regression based approach was shown to be effective for this task (Ellis et al. 2018). This work explores the use of non-linear artificial neural networks (ANNs) as well as a "domain adaptation" (DA) training approach, which aims to reduce issues resulting from dataset-specific biases (also referred to as "batch effects"). The results of a series of thorough experiments involving phenotype prediction on SRA and TCGA samples indicate that the ANNs as well as the DA training approach improve upon the performance of the prior linear regression model. The authors then use their methods to provide phenotype labels for SRA samples missing this information.

I fully agree that improvements to the metadata for databases such as the SRA are important, both for more accurate retrieval of relevant datasets as well as for large-scale statistical meta-analyses or machine learning with data in these databases. I also agree that methods addressing dataset-specific biases, or batch effects, are critical in this context. Thus, the DA approach introduced in this manuscript is of great interest.

Overall, I found the manuscript to be well written and the experiments quite thorough. However, I have a few concerns regarding the evaluations that I believe need to be addressed in order for the accuracy improvements to be convincing.

Major comments:

1. A priori, I would expect prediction of sex from gene expression data would be a relatively trivial task using the counts of reads mapping to the X and Y chromosomes. Figure S1 confirms this expectation, at least for the GTEx and TCGA datasets: the male and female samples are easily, and linearly, separable. Thus, I was surprised that there were accuracy gains with the ANNs for this task. Looking at the right (SRA) panel in Figure S1, a major concern here is that the ground-truth sex labels on the SRA samples, which were used for the test set, are likely incorrect for a non-negligible number of samples. Because of this issue, it is possible that the ANNs are actually learning to predict such samples *incorrectly* in truth (but correctly with respect to the test labels). For example, perhaps the MetaSRA is systematically assigning an incorrect sex label to certain cell lines and the ANNs are then learning features of those cell lines that allow them to predict the sex correctly with respect to the MetaSRA label, but incorrectly in truth. A thorough investigation into the apparent performance gains for sex prediction would help to clear up this issue.

This is actually a great comment and yes, the assumption that the model might overfit if the training data would contain a considerable amount of false annotations is quite conceivable. We therefore first performed an exploratory analysis of potential misannotations in the SRA dataset by investigating the range of chrY total sum counts per data source (see figure below).

Fig. S4. Misclassification in MetaSRA. Histogram of the total sum of normalized counts mapped to the chrY for GTEx, TCGA and SRA. Male and female clearly overlap in SRA, indicating mislabeling by MetaSRA.

The figure, which was added to the revised manuscript, shows histograms of all samples of GTEx, TCGA and SRA, respectively. Plotted are the sum counts on the

Powered by Editorial Manager® and ProduXion Manager® from Aries Systems Corporation

chrY. The plot supports the notion that there are many FEMALE labeled samples that have a high chrY expression in the SRA data (and vica-versa). A threshold for chrY total count sum was chosen to clearly identify true labels. Because some GTEx and TCGA samples have a non-zero chrY sum count, we picked a threshold to define FEMALE at sum count chrY <= 2. Given this threshold we identified 366 of 3240 SRA samples labeled FEMALE to be above that threshold (i.e. they are probably MALE). Next we observed that 271 of these 366 samples were in the training set (FEMALE n=1,017, MALE n=1,246). If the model overfit on this wrongly labeled data, the trained model would predict the wrongly labeled training data wrong (i.e. with the training label FEMALE, and not with the likely correct label MALE). In other words, the potentially mis-annotated true male samples (falsely annotated as female) amount to 21.7% of all male samples (for females 25.3%). The DA model assigned 220 of these 271 training samples as FEMALE. We take this as evidence that the model overfit on the training data. In our answer to comment 2 by the reviewer we show that the ANN starts overfitting if >20% of the training data of a single class is incorrectly annotated, as is the case here. A similar observation was made for the samples annotated as MALE by MetaSRA that are above the chosen threshold.

To avoid overfitting of the model on wrong labels, we cleaned the SRA training and test set and removed all ambiguous samples according to the sum count chrY threshold stated above. This significantly changed the results for SEX phenotype prediction. For example, the DA model now predicts at 0.99 accuracy compared to 0.93 with the unfiltered training data. The difference in classification accuracy between the different models (LIN, MLP and DA) is now in the range of 1%. We still observed a small performance increase for the ANN models and decided to keep the phenotype in the study but gave it less weight by moving it into the supplementary figures. The SEX phenotype was removed from the main results figure 3 and merged with supplementary figure 8.

In the method subsection Phenotype Classification Experiments, we changed the paragraph about the sex phenotype from 'Sex: In total, 159 SRA studies contained samples annotated with male and or female by MetaSRA. These studies were combined into the training set (studies=78, n=2,317), and test set (studies=81, n=923) (Supplementary Tables 2 and 3). For model validation, GTEx was randomly split into training and test sets with an 80:20 ratio for both sex and tissue classification.' to 'Sex: We noticed SRA samples identified as female by MetaSRA to have a significant amount of reads mapped to chrY (Supplementary Figure 4). All samples labeled as female with a total normalized count >= 2 and all samples labeled male with a total normalized count <2 were removed. In total, 149 SRA studies contained samples annotated with male and or female by MetaSRA. These studies were combined into the training set (studies=73, n=2,017), and test set (studies=76, n=791) (Supplementary Tables 2 and 3). For model validation, GTEx was randomly split into training and test sets with an 80:20 ratio for both sex and tissue classification.'

Supplementary Table 2,3 and 6 were adjusted according to the new data set sizes and experiment results.

As suggested by the reviewer, we then sought to correct potential mis-annotations of the MetaSRA data by predicting their labels using the model trained on the high-confidence annotations. We used the MLP G+S model to predict the true corrected label for the removed SEX samples. For 82% of the 132 filtered samples, the MLP model predicted the opposite of the presumably wrong MetaSRA labels. However, our MLP model was able to confirm the MetaSRA label for 24 samples. These samples had a mean chrY count sum of 2.4 (i.e. close to the cutoff value). We were able to manually confirm some samples. For example, SRR1164833, SRR1164787 and SRR1164842 are samples from a prostate cancer study labeled as MALE by MetaSRA. Our MLP model correctly classified these samples despite the fact that their chrY total sum count was between 0.4 and 1.4. On the other hand, SRR16076 54 / 56 / 61 / 62 / 64 / 65/ 70 / 71 are annotated as FEMALE by MetaSRA and the MLP but had a chrY total sum count of 2-5.3. Because the MLP is able to correctly classify these borderline cases, we are convinced that no overfitting on the training data is taking place.

These findings were added to the results section 'ANN Models Can Correct Mislabeling in MetaSRA', which summarizes the new results obtained answering the reviewers question 1-3 (paragraph shown as answer to question 2).

2. Related to comment #1, the same issue is also a concern for the prediction of tissue

Powered by Editorial Manager® and ProduXion Manager® from Aries Systems Corporation

in the SRA, and potentially also for sample source. That is, if there are systematic annotation errors by the MetaSRA with respect to tissue of origin, the ANNs could actually be learning and propagating these systematic errors. Because the linear regression model is more limited, it is less able to learn such errors and is, in fact, more robust to them. In summary, the authors should provide some evidence that the presented performance gains are not largely due to learning such systematic label errors in the MetaSRA. Note that the MetaSRA is the result of an automated pipeline, not manual curation, so a certain fraction of errors are to be expected.

Again, a great comment and suggestion by the reviewer. We fully agree with the reviewer that a certain fraction of errors is to be expected in the MetaSRA annotation. An overfitting experiment was designed, to investigate the possibility that the ANN models overfit on wrongly annotated data. In a nutshell, the ANN models predict correctly when the level of mis-annotations in the training set does not exceed ~20%, above ~20% mis-annotations result in progressively increasing model overfitting. We added the following text to the methods section: '
Test for Overfitting
MetaSRA provides labels for SRA data generated in an automated way. We have identified mislabeled samples for the sex phenotype (see Methods). The following experiment was designed to test the ANN based model's susceptibility to overfitting on mislabeled training data. An MLP model was trained on GTEx data on four tissue classes (i.e., brain, esophagus, lung and skin). A range of fractions of the brain samples were randomly assigned to skin tissue (i.e., 0.01,0.025,0.05,0.1,0.20,0.5 and .8). The model was then trained on GTEx samples of the four classes, including the mislabeled brain samples. We tested the models overfitting capabilities by letting it predict the label of the mislabelled brain samples. If the model overfits, these samples should be predicted to be from skin tissue. The same experiment was conducted for the sex phenotype by mislabeling male samples as female.'. We also added a novel Fig. S10 to the manuscript, showing stable prediction performance of the ANNs with training data mis-labels of up to ~20%: '

Fig. S10. Test of Overfitting. An MLP model was trained on GTEx data. An increasing fraction of one class was assigned a wrong class label (e.g., brain to skin). The model was trained on the partially mislabeled data and the mislabeled data was predicted by the model after training. We quantify the model's susceptibility to overfitting by letting it correct the mislabeled training data. The MLP model was able to correct all mislabeled data up to a mislabeling fraction of 20%. We conclude that the ANN models are very robust in dealing with mislabeled data.

The following text was added to the results section: '
ANN Models Can Correct Mislabeling in MetaSRA
Given the difficulties with metadata standards in SRA data, mislabeling in MetaSRA is to be expected. To understand if and when ANN models would overfit on mislabelled MetaSRA data, we trained an MLP on partially mislabeled samples (see Methods). Supplementary Figure 10 shows that the MLP model correctly predicts brain samples, even if they were presented as skin samples during model training. A decrease of this accuracy was observed if more than 20\% of all brain samples were mislabeled as skin. A similar observation was made for the sex phenotype (Supplementary Figure 10). We concluded that our models are robust if less than 20\% mislabelled data is present during training. More importantly, these models can be used to correct mislabeled MetaSRA data.
In the specific case of sex classification, the MLP G+S was used to predict the true corrected label for the SEX samples that were removed from training due to low sex-chromosome counts (see Methods). For 82% of the 132 filtered samples, the MLP model predicted the opposite of the presumably wrong MetaSRA labels. However, our MLP model was able to confirm the MetaSRA label for 24 samples. These samples had a mean chrY count sum of 2.4 (i.e. close to the cutoff value). Manual confirmation revealed a high model accuracy. For example, SRR1164833, SRR1164787 and SRR1164842 are samples from a prostate cancer study labeled as MALE by MetaSRA. Our MLP model correctly classified these samples despite the fact that their chrY total sum count was between 0.4 and 1.4. On the other hand, SRR16076 54 / 56 / 61 / 62 / 64 / 65/ 70 / 71 are annotated as FEMALE by MetaSRA and the MLP but had a chrY total sum count of 2-5.3. We see the correct classification of these

Powered by Editorial Manager® and ProduXion Manager® from Aries Systems Corporation

borderline cases as further evidence that no overfitting is taking place.
A list of all SRA samples for which the MetaSRA labels and the predicted labels mismatched is available in the Supplementary Material.'. We thank the reviewer for this great comment and hope that the revised manuscript builds a strong case for the stability of the approach taken.

3. Also continuing the line of thought from comments #1 and #2, an additional major application of phenotype prediction is *correction* of mislabeled samples, but this is not discussed in this manuscript. I don't think the authors necessarily need to demonstrate this application (and in fact they do briefly in the "Prediction of SRA Sex" section of the results), but a deeper analysis of this might go hand in hand with addressing comments #1 and #2.

Another valuable suggestion, which we have addressed in the answers to comments #1 and #2 (and the revised document). We really think that the first three main comments of the reviewer and our investigation into them strengthened the overall quality of the manuscript considerably.

4. An important contribution of this work is the set of newly-predicted phenotype labels. I cannot find mention of where this set can be accessed. Perhaps it can be archived at a site such as Zenodo, if it is not already.

We apologize if we did not make the newly-predicted phenotype labels available to the reviewer in the initial submission. We have now uploaded all supplementary data to gigadb.org, as requested by the editor. In addition, the data can now also be downloaded from the git page https://github.com/imsb-uke/rna_augment/tree/master/supplementary%20material

Minor comments:

5. In the introduction, the authors describe some prior DA approaches and then state that "All these methods have been implemented and applied by us for RNA-seq phenotype prediction and found not to be scalable to a situation with hundreds of different and scarce target domains, encountered, for instance, in the SRA." This would seem to be a result rather than a statement of prior facts, and should be moved to the results section (ideally with experiments), unless this was shown in a prior publication.

We completely agree that our work on other architectures that did not provide good results are results rather than background information. We fear, however, that expanding on these 'negative' results too much would further complicate and lengthen a manuscript that is already quite long and non-trivial. In agreement with the reviewer, we therefore added an extended text to the novel methods section 'Other Models': '
Other Models
While developing our DA model we did a thorough literature research and implemented and tested multiple architectures and strategies. Here we give a brief overview of the models we found not suitable for the problem of bias invariant RNA-seq metadata annotation. The first strategy that has been tested was interpolation between source and target domain by training feature extractors on an increasing ratio of target to source domain data. The second strategy was adversarial training by applying two loss functions. The first loss function forces the model to learn weights for the class prediction task, while the second forces the model to learn to ignore differences between the source and target domain. We also implemented Tzeng's [ref?] adaptation of this idea, proposing a model using a separate source and target encoder, using them as 'real' and generator input for a generative adversarial network that is capable of ignoring bias. These models ultimately failed due to the hundreds of dataset biases in the SRA data and their relatively small sample size (data not shown). For the case of scarce target data an approach was previously proposed using Siamese networks. The trained model achieved an msa of 0.83 and mca of 0.79 for tissue classification on SRA data. The mca achieved is comparable to the results of the MLP model, however, the msa score is 6% lower than even the LIN model. The more challenging task of learning to map the bias embedding into the pre-learned class embedding, as presented in this paper, finally resulted in the desired outcome.'. In addition, the beginning of the methods section 'Model Architecture' was changed from 'Our DA architecture is based on the Siamese network architecture.' to 'Our DA architecture is

Powered by Editorial Manager® and ProduXion Manager® from Aries Systems Corporation

based on the Siamese network architecture. A Siamese network usually shares the weights between two equal networks, here however, we do not use weight sharing. Weight-sharing and other types of architecture did not prove to be applicable to this problem (see Methods section Other Models).' to reference the novel section. Again, we thank the reviewer for this excellent comment, which increased the quality of our manuscript.

6. At the beginning of the methods section, I found the phrase "which we define as the number of unique dataset biases present within one data source" confusing. Only later did I come to understand that this was simply referring to the number of studies. I think this could be made clearer earlier in the text. Also, that phrase references Fig S1, which shows the sex labels on the samples from each source, and doesn't really show the heterogeneity of the source. It is unclear why that figure is referenced here.

We thank the reviewer for pointing out this misleading reference. To correct this mistake, we have modified the beginning of the first paragraph of subsection "Data Acquisition". 'To train and test models we gathered data from three different sources, each with a different level of homogeneity, which we define as the number of unique dataset biases present within one data source (Supplementary Figure 1).' to 'To train and test models we gathered data from three different sources (i.e. GTEx, TCGA and SRA), each with a different level of heterogeneity (Supplementary Figure 1). We measure data source heterogeneity by the number of unique dataset (or studies) in the source. Each dataset (or study) is believed to have a unique bias.'
We also agree that the current supplementary figure does not visualize the data heterogeneity in the data sources sufficiently. We thus decided to replace it with the following figure:

Fig. S1. Visualizing Data Set Bias. GTEx is a single-study data source, while SRA is a multi-study data source. A) T-SNE plot of gene expression values of GTEx and B) SRA samples, belonging to five different tissues. The GTEx data is more coherently clustered compared to the SRA data. The individual studies in the SRA data appear to form less homogeneous clusters, indicating a larger within-variance in the data source.

We believe that these changes better reflect the data and claims of our study and thank the reviewer for the suggestions.

7. Sample source definition: I understand that the MetaSRA sample type classifications were used, but it is not clear to me how they were mapped to "biopsy" and "lab grown cell line" categories. It sounds like "tissue" was mapped to "biopsy" but I'm not sure about the rest. One MetaSRA category is "primary cells", which can be cells sorted from a disassociated (biopsied) tissue sample. Are those also considered "biopsy?"

We agree with the reviewer that there is potential for confusion between the phenotype tissue and the category TISSUE of phenotype sample source. Therefore, we renamed the MetaSRA category TISSUE to biopsy. Throughout the text (e.g. in the caption of Figure 1) we try to emphasize this by writing "tissue (e.g. lung, heart)". To make this even more clear, we added the following three sentences to the method section Phenotype Classification Experiments. Below we marked in red the added information:'
Phenotype Classification Experiments.
Tissue: To ensure that … For model validation GTEx was randomly split into training and test sets with an 80:20 ratio for both sex and tissue classification. Sample Source: A confidence cutoff of >= 0.7 was applied (provided by MetaSRA), reducing the total amount of annotated samples for SRA from 23,651 to 17,343. MetaSRA provided six different types of sample source. The two largest classes, TISSUE and CELL LINE were selected. In this study we renamed the MetaSRA label TISSUE to biopsy to not be confused with the phenotype tissue (e.g., heart, lung, skin). For each of the two selected categories we sorted all available studies by number of samples, placed the first third of studies into the training (studies=420, n=12,725), the second third into the test (studies=422, n=3,144) and the last third into the SRA validation set (studies=418, n=1,124) (Supplementary Tables 2 and 3). A list of the sample ids and corresponding

Powered by Editorial Manager® and ProduXion Manager® from Aries Systems Corporation

labels is available in the Supplementary Material.'.
The reviewer also mentioned the MetaSRA term PRIMARY CELLS. It is true that the MetaSRA defines PRIMARY CELLS as a subtype of TISSUE in their hierarchical classification (supplementary figure 6, Bernstein et al. 2017). However, we believe that samples obtained from biopsies are split into altered and unaltered cells. The full name of the PRIMARY CELLS label is PRIMARY SPECIALIZED CELLS which would indicate some kind of alteration to the sample. PRIMARY CELLS and the other 3 categories that are not TISSUE or CELL LINE were thus mapped to "others" (the "catch-all") class during the annotation phase. We now specify this in the result section "Prediction and Availability of Novel Metadata" with the following sentence: 'Specifically, we first trained a new MLP model to identify the sample source biopsy vs. all other sample sources available in the SRA data as defined by MetaSRA.'. We hope that these changes help readers understand the terminology used in this manuscript.

8. What was used for "gene length" to normalize to TPM?

We extracted the 'gene length' from Gencode v25, GRCh38, 07.2016. We have integrated this information in the revised document section Dimensionality Reduction and Normalization 'First standard log2 Transcript per Million (TPM) normalization was applied to normalize for gene length (Gencode v25, GRCh38, 07.2016) and library size.'.

9. "Metadata annotation" section: I did not understand the phrase "no samples were discharged because of their tissue label." Are samples being removed from the training or test sets with some criteria?

This sentence is indeed hard to understand. In brief, we downloaded ~50,000 SRA samples from recount2 and selected the samples with a tissue label belonging to the 16 classes. For the annotation we take all samples. The samples not belonging to one of the 16 tissues goes into a specially "catch-all" class.
In the revised document we have removed 'no samples were discharged because of their tissue label.' from the paragraph as we believe the next sentence 'Samples from a tissue class other than the original 16 classes were pooled together into a 'catch-all' class, resulting in 17 classes.' makes the point perfectly clear. We hope that this change adequately addresses the reviewer's justified critique.

10. DA model architecture: the text says that the model "is trained on semi-hard triplets" and then gives a definition of this based on Euclidean distances in the embedding spaces. This confuses me because bias embedding mapper (BM) is what is being trained here, so this appears circular. How do you get the distances without already having the BM?

We are sorry for not clarifying this better in the first manuscript. The BM is pretrained on the source domain, as it is a direct copy of the trained SM. We have changed 'For a second training cycle, the bias mapper is created with the same architecture as the SM. The CL is removed and the weights of the SM are frozen. Triplets of data are forward propagated through the BM and SM in parallel (Figure 2C).' to 'For the second training cycle, the SM and the CL are separated and their weights frozen. Frozen weights are not updated during the second training cycle. The bias mapper is created by copying the architecture and weights of the trained source mapper. SM and BM are trained on triplets drawn from the source and the bias domain. Samples from the source domain are passed through the SM, samples from the bias domain through the BM at the same time (Figure 2C).' in the revised document. In addition, we changed 'Where d(i,j) are the distances in embedding space between the respective outputs of the BM and SM on samples i and j.' to 'Where d(i,j) are the distances between the constricted embedding space of the SM and the bias mapping into that space of the BM on samples i and j.'.
We also added the new section 'Other Models' to the methods, introducing the msa and mca achieved with a Siamese network where weights are shared between the SM and BM. (see response to minor comment 5).

11. Figure 3D: y-axis appears to be mislabeled

We thank the reviewer for pointing out this mistake. In the revised document, we have

corrected the y-axis labeling of what is now Figure 3C.

Reviewer #2: Summary:
The authors present a Domain adaptation model that uses a Siamese network architecture to lead missing metadata from bulk RNAseq data and compare the performance to a previously published linear regression model (LIN) and a multilayer perceptron (MLP). As data sources, the authors used GTEx, SRA, TCGA. The DA model outperforms the LIN and MLP, when many classes to learn (e.g. in case of tissues), but not in the case of sex and sample source. While the authors present their work in a concise and clear way, I think that they can improve their manuscript in several points.

Major:

I did not see a cross-validation of any of the used models. Instead, the authors varied the random seeds for model initialization. While I appreciate the split by study in the case of the SRA data, I think that the authors should add a cross-validation approach on the model training to increase robustness, even if that means that the training dataset has varying size.

Overfitting is a pivotal concern in any Machine Learning task, and we believe the reviewer addresses a very critical point. Domain adaptation methods such as Siamese Neural Networks have been developed to overcome the problem of overfitting, by learning latent space representations that reliably map two (or more) biases to the same manifold.
To understand if and when our ANN models start to overfit, we chose to simulate mis-labelled data in the training sets of the models and subsequently observed ‘overfitting’ of the model based on ‘wrong’ predictions (predicting the mis-annotated class of the data).
In a nutshell, the ANN models predict correctly when the level of mis-annotations in the training set does not exceed ~20%, above ~20% mis-annotations result in progressively increasing model overfitting. We added the following text to the methods section: ‘
Test for Overfitting
MetaSRA provides labels for SRA data generated in an automated way. We have identified mislabeled samples for the sex phenotype (see Methods). The following experiment was designed to test the ANN based model's susceptibility to overfitting on mislabeled training data. An MLP model was trained on GTEx data on four tissue classes (i.e., brain, esophagus, lung and skin). A range of fractions of the brain samples were randomly assigned to skin tissue (i.e., 0.01,0.025,0.05,0.1,0.20,0.5 and .8). The model was then trained on GTEx samples of the four classes, including the mislabeled brain samples. We tested the models overfitting capabilities by letting it predict the label of the mislabelled brain samples. If the model overfits, these samples should be predicted to be from skin tissue. The same experiment was conducted for the sex phenotype by mislabeling male samples as female.’. We also added a novel Fig. S10 to the manuscript, showing stable prediction performance of the ANNs with training data mis-labels of up to ~20%: ’

Fig. S10. Test of Overfitting. An MLP model was trained on GTEx data. An increasing fraction of one class was assigned a wrong class label (e.g., brain to skin). The model was trained on the partially mislabeled data and the mislabeled data was predicted by the model after training. We quantify the model's susceptibility to overfitting by letting it correct the mislabeled training data. The MLP model was able to correct all mislabeled data up to a mislabeling fraction of 20%. We conclude that the ANN models are very robust in dealing with mislabeled data.

The following text was added to the results section: ‘
ANN Models Can Correct Mislabeling in MetaSRA
Given the difficulties with metadata standards in the SRA state above, mislabeling in MetaSRA is to be expected. We designed an overfit test for the ANN models where we trained an MLP on partially mislabeled samples (see Methods). Supplementary Figure 10 shows that the MLP model correctly predicts brain samples, even if they were presented as skin samples during model training. A decrease of this accuracy was

Powered by Editorial Manager® and ProduXion Manager® from Aries Systems Corporation

observed if more than 20% of all brain samples were mislabeled as skin. A similar observation was made for the sex phenotype (Supplementary Figure 10). We concluded that our models can be used to correct mislabeled MetaSRA data.
In the specific case of sex classification, the MLP G+S was used to predict the true corrected label for the removed SEX samples. For 82% of the 132 filtered samples, the MLP model predicted the opposite of the presumably wrong MetaSRA labels. However, our MLP model was able to confirm the MetaSRA label for 24 samples. These samples had a mean chrY count sum of 2.4 (i.e. close to the cutoff value). Manual confirmation revealed a high model accuracy. For example, SRR1164833, SRR1164787 and SRR1164842 are samples from a prostate cancer study labeled as MALE by MetaSRA. Our MLP model correctly classified these samples despite the fact that their chrY total sum count was between 0.4 and 1.4. On the other hand, SRR16076 54 / 56 / 61 / 62 / 64 / 65/ 70 / 71 are annotated as FEMALE by MetaSRA and the MLP but had a chrY total sum count of 2-5.3. We see the correct classification of these borderline cases as further evidence that no overfitting is taking place.
A list of all SRA samples for which the MetaSRA labels and the predicted labels mismatched is available in the Supplementary Material.'. We thank the reviewer for this great comment and hope that the revised manuscript builds a strong case for the stability of the approach taken. While we have not used a cross-validation approach to assess and minimize model overfitting, we hope that our results are compelling enough to convince the reviewer of the robustness of our approach. One of the reasons we chose not to go via cross-validation is the training set size differences that would make cross-validation results hard to compare (as the author also correctly stated). On another note, it might be interesting for the reviewer to also read our response to reviewer 1's first main concern (overfitting of sex annotations), which further proves the validity of the reviewer's excellent comment.

Page 8: The introduction of the "percentage point" (ppt) as metric is superfluous. I suggest to use percent (%) instead, if the authors really want to state relative changes. Further, I have the impression that ppt and % are used synonymously, so the authors should use the unit consistently.

We apologize for this unnecessary confusion. The ppt was dropped, and the result section was changed accordingly.

Figure 3: Plotting the relative change to the baseline model overrates the actual improvement of the DA approach and is not statistically sound. I suggest to state absolute changes in accuracy (or mca/msa) instead.

We thank the reviewer for pointing out this problem. In the revised manuscript, we have changed figure 3 to show absolute accuracy. To highlight changes, we took the liberty to rescale the X-axis from ~0.6 to 1. In case the reviewer deems it necessary to show the full spectrum from 0 to 1 we would be more than willing to adjust the X-axis.

Statistical analysis page 8: When using a t-test, the authors assume that the msa/mca scores would be normally distributed around some unknown mean value. By looking at the boxplots, I think that the assumption is not necessarily true and I suggest to use a non-parametric test (e.g. Wilcoxon rank sum test) instead of the t-test.

This is an excellent observation, and we have replaced the t-test in the Prediction of SRA Tissue section with a non-parametric Mann-Whitney test and changed the text of the revised manuscript accordingly.

The method subsection Statistical Tests was changed from 'Accuracy distributions for sex and tissue prediction were tested for statistically significant differences using a t-test (two distributions, scipy.stats.ttest_ind v 1.3.1) or ANOVA (more than two distributions, scipy.stats.f_oneway) with a significance threshold of 0.01.' to 'Accuracy distributions were tested for significance using the non-parametric Mann-Whitney-U-Test (scipy.stats.mannwhitneyu v 1.3.1).'

Identifying novel training data (page 10): I would be very careful in including predicted labels as ground truth data (which is known as data imputation task). In these cases, retraining a classifier on both ground truth and predictions will lead to overfitting and

Powered by Editorial Manager® and ProduXion Manager® from Aries Systems Corporation

spurious results, when the predicted labels dominate the ground truth labels.

The reviewer states that re-training on predicted data could result in overfitting, which is per se a valid concern. We would like to highlight, however, that 'E/M-like' approaches that train iteratively on 'harder' samples have shown to give superior classification and regression performances in several contexts. We agree that we haven't shown beyond reasonable doubt that in this case the re-training is not overfitting, which is why we have decided to remove this last paragraph. We thank the reviewer for raising this very good point of critique.

Minor:

Page 4: Gene selection based on Gini index: was there an overlap of the genes used for tissue and sample source classification? I would assume so from the setup with the range of Gini indices.

This is an interesting question, which we followed up upon. A list of all used genes per phenotype is now available for download at giga.db. The file input_features.xlsx contains the list of genes used for each phenotype and the intersection between the phenotypes. The sex and sample source phenotype share 166 input features, sex and tissue phenotype 155, and sample source and tissue share 2976 input features.

Figures and Subfigures are not fully in the order of first appearance (esp. subfigures 1 B-C compared to figure 2).

Unfortunately, we cannot find the noted inconsistent labeling of Figure 1 (B) compared to Figure 2.

Figure 1A: Datasets could be visualized as bar charts to give the reader an idea about the dataset sizes. Exact numbers can be stated in the supplement or figure legend

While we agree with the reviewer on almost every other point raised, we beg to differ here. We think that the number of samples per data source is of secondary importance in this study, it is rather the number of biases in the training data that is of utmost relevance. In case the reviewer and the editor insist on the suggested change, however, we will comply.

Figure 1: The figure design is clean, however, the green TCGA box is not colorblind friendly and difficult to distinguish from the purple GTEx box. Consider a lighter color.

We thank the reviewer for this valid comment, we have changed the TCGA box to yellow in the revised manuscript.

Figure 3:
I struggle to understand the plot, especially the numbers at the top of each boxplot (please clarify in the figure legend, that this is msa and mca).
What was the baseline model in Figure 3D? Why do you present this as main figure, when the relative changes are within 1% (and therefore within the range of the noise level)?

We have significantly revised the figure and legend, in accordance with the reviewer's excellent suggestions and valid points of critique.

Supplementary Figure 7A:
The PCA analysis of the ovary data is interesting. The differences of TCGA-ovary and GTEx-ovary data is reflected in PC1 and I was wondering whether the authors could give a more detailed explanation on the reasons for this systematic bias, e.g. by analysing the loadings of PC1.

We agree with the reviewer, that the observed shifts in the GTEx and TCGA domain are of great interest and it could be informative to scrutinize the loadings of the respective PC1. Unfortunately, we did not find any biological pathway or category that might be enriched in PC1 when performing a PANTHER pathway enrichment analysis on the top 1% and top 5% genes (ranked according to absolute loading). Also, just

Powered by Editorial Manager® and ProduXion Manager® from Aries Systems Corporation

	looking at the genes themselves did not yield any insights to us, which might be due to the fact that we do not possess enough biological understanding of the matter. Therefore, we were not able to come up with any biological explanation of the shift between TCGA-ovary and GTEx-ovary data in PC1.<br>We would like to stress, however, that our manuscript is already quite large and complex and we fear that a closer inspection of genes and pathways responsible for cluster differences might be outside the scope of this study. We will include this information into a revised version if the reviewer or editor deems it relevant.<br><br>Page 9: Clarify. "For example, SRP056612 is a study on the effect of the coronavirus on cultured kidney and<br>lung cells [39] and SRP045611 is a study involving HEK cells, which lack the Y chromosome but are annotated as male by MetaSRA [40]."<br>As far as I understood the cited reference, it corresponds to the MERS coronavirus.<br><br>We thank the author for noting this and we corrected this in the revised manuscript.<br><br>Second, HEK cells are (most likely) of female origin, therefore, clearly state the nature of the mislabeling (I consider this as human error in the MetaSRA).<br><br>We completely agree with the reviewer that the annotation error is in the MetaSRA data. The nature of the error is either, as the reviewer suggested, a human error during submission, or a mapping error of the MetaSRA pipeline. Since we don't know which of the two is the problem we opted to not state the nature of the error in more detail.<br><br>By the way, line numbers would have been nice to comment on certain passages.<br><br>We absolutely agree with the reviewer, line numbers can greatly facilitate the review process. We tried to adhere to the GIGA Science submission guidelines, which unfortunately do not mention line numbers.
**Additional Information:**	
**Question**	**Response**
Are you submitting this manuscript to a special series or article collection?	No
**Experimental design and statistics**<br><br>Full details of the experimental design and statistical methods used should be given in the Methods section, as detailed in our Minimum Standards Reporting Checklist. Information essential to interpreting the data presented should be made available in the figure legends.<br><br>Have you included all the information requested in your manuscript?	Yes
**Resources**<br><br>A description of all resources used, including antibodies, cell lines, animals and software tools, with enough information to allow them to be uniquely	Yes

identified, should be included in the Methods section. Authors are strongly encouraged to cite Research Resource Identifiers (RRIDs) for antibodies, model organisms and tools, where possible.<br><br>Have you included the information requested as detailed in our Minimum Standards Reporting Checklist?	
**Availability of data and materials**<br><br>All datasets and code on which the conclusions of the paper rely must be either included in your submission or deposited in publicly available repositories (where available and ethically appropriate), referencing such data using a unique identifier in the references and in the "Availability of Data and Materials" section of your manuscript.<br><br>Have you have met the above requirement as detailed in our Minimum Standards Reporting Checklist?	No
If not, please give reasons for any omissions below.<br><br>as follow-up to "**Availability of data and materials**<br><br>All datasets and code on which the conclusions of the paper rely must be either included in your submission or deposited in publicly available repositories (where available and ethically appropriate), referencing such data using a unique identifier in the references and in the "Availability of Data and Materials" section of your manuscript.<br><br>Have you have met the above requirement as detailed in our Minimum Standards Reporting Checklist?	All data used is publicly available, however, the specific datasets we created and used for training and testing are not (yet). It is our understanding that, if this manuscript will be send for review, we are given the opportunity to upload them to GigaDB.

Powered by Editorial Manager® and ProduXion Manager® from Aries Systems Corporation

"	

Powered by Editorial Manager® and ProduXion Manager® from Aries Systems Corporation

Click here to access/download;LaTeX - Main Document (TeX file);oup-authoring-template.tex

```
This is pdfTeX, Version 3.14159265-2.6-1.40.21 (TeX Live 2020/W32TeX)
(preloaded format=pdflatex 2020.5.12)  11 JUN 2021 07:31
entering extended mode
 restricted \write18 enabled.
 %&-line parsing enabled.
**oup-authoring-template.tex
(./oup-authoring-template.tex
LaTeX2e <2020-02-02> patch level 5
L3 programming layer <2020-05-05>

! LaTeX Error: File `oup-authoring-template.cls' not found.

Type X to quit or <RETURN> to proceed,
or enter new name. (Default extension: cls)

Enter file name:
! Emergency stop.
<read *>

l.26 ^^M

*** (cannot \read from terminal in nonstop modes)


Here is how much of TeX's memory you used:
 21 strings out of 480681
 570 string characters out of 5908536
 236875 words of memory out of 5000000
 15943 multiletter control sequences out of 15000+600000
 532338 words of font info for 24 fonts, out of 8000000 for 9000
 1141 hyphenation exceptions out of 8191
 14i,0n,17p,182b,10s stack positions out of
5000i,500n,10000p,200000b,80000s
!  ==> Fatal error occurred, no output PDF file produced!
```

Click here to access/download;Manuscript;GIGA_revision.pdf

Journal Title Here, 20xx, pp. 1–32

doi: DOI HERE
Advance Access Publication Date: Day Month Year
Paper

PAPER

Bias invariant RNA-seq metadata annotation

Hannes Wartmann,[1] Sven Heins,[1] Karin Kloiber[1] and Stefan Bonn[1,*]

[1]Institute of Medical Systems Biology, Center for Biomedical AI, University Medical Center Hamburg-Eppendorf, 20251, Hamburg, Germany
[*]Corresponding author. sbonn@uke.de
FOR PUBLISHER ONLY Received on Date Month Year; revised on Date Month Year; accepted on Date Month Year

Abstract

Background: Recent technological advances have resulted in an unprecedented increase in publicly available biomedical data, yet the reuse of the data is often precluded by experimental bias and a lack of annotation depth and consistency. Missing annotations makes it impossible for researchers to find datasets specific to their needs. **Findings**: Here we investigate RNA-seq metadata prediction based on gene expression values. We present a deep-learning based domain adaptation algorithm for the automatic annotation of RNA-seq metadata. We show how our algorithm outperforms existing linear regression based approaches as well as traditional neural network methods for the prediction of tissue, sample source, and patient sex information across several large data repositories. By using a model architecture similar to Siamese networks the algorithm is able to learn biases from datasets with few samples. **Conclusion**: Using our novel domain adaptation approach we achieved metadata annotation accuracies up to 15.7% better than a previously published method. Using the best model we provide a list of more than 10,000 novel tissue and sex label annotations for 8,495 unique SRA samples. Our approach has the potential to revive idle datasets by automated annotation making them more searchable. The source code as well as an example are available at: `github.com/imsb-uke/rna_augment`

Key words: RNA-seq metadata, data reusability, automated annotation, machine learning, domain adaptation, bias invariance

Introduction

Next generation RNA-sequencing (RNA-seq) has been a pillar of biomedical research for many years [1, 2]. It allows researchers to simultaneously quantify and compare the expression of tens of thousands of genomic transcripts. A continuous drop in cost makes RNA-seq a widely available method of choice to uncover the molecular basis of biological development and diseases [3, 4]. As a result of this, recent years

© The Author 20xx. Published by Oxford University Press. All rights reserved. For permissions, please e-mail: journals.permissions@oup.com

have seen a strong growth in publicly accessible RNA-seq data. The actual reuse and integration of this data, however, has been largely limited by the lack of consistent metadata annotation and individual dataset bias [5, 6]. The lack of metadata annotation for RNA-seq samples, such as tissue of origin, disease or sex phenotype, prohibits experimenters from finding data that is relevant to their research. Moreover, dataset biases [7] due to differences in protocols and technologies [8] or of a biological nature hinder integration and comparative analysis.

To allow for efficient data reuse, publicly available data has to be harmonized and well annotated with standardized metadata and subsequently be made accessible (and searchable) [9]; This practice is followed by the Genotype-Tissue Expression Project (GTEx) [10], and The Cancer Genome Atlas (TCGA). Nevertheless, the primary database for next-generation sequencing projects, the Sequence Read Archive (SRA) [11], stores raw sequencing information that lacks rigorous standards of curation, which limits the reusability of its data.

Efforts to predict missing or sparse metadata in public RNA-seq resources have shown promising results. For instance, recently published studies used text mining approaches to retrieve missing annotation from associated abstracts or free text annotations in the data sources [12, 13, 14]. Others have used RNA-seq expression values for phenotype prediction. For example, machine learning (ML) has successfully been applied to disease and cell type classification [15, 16] or survival outcomes on TCGA data [17]. Others have taken advantage of prior domain knowledge such as gene regulatory networks for enhanced feature selection [18, 19]. Recently a linear regression model fitted to GTEx data has been presented for the prediction of tissue, sex and other phenotypes of SRA and TCGA samples [20]. These efforts provide evidence that missing RNA-seq metadata can be successfully predicted based on genomic expression values using ML approaches.

Artificial neural networks (ANNs) in their various forms and functions consistently outperform classical ML approaches in a large variety of biological tasks, including classification, data generation and segmentation [21, 22, 23, 24]. Given large training datasets, these algorithms can learn complex representations of data by automatically weighting and combining features non-linearly. This has led us to hypothesize that ANN based models could increase the performance in metadata prediction beyond that of classical ML approaches such as linear regression. Of special interest in this context is domain adaptation (DA) [25], a subfield of ML which aims to specifically alleviate problems conferred by dataset bias [26]. The aim of DA is to build and train ANNs on a source domain in such a way that the model performs well on a biased target domain.

Here we present a DA approach capable of leveraging a number of dataset biases, boosting generalizability of phenotype prediction. We developed the model using three data sources (GTEx, TCGA and SRA) of different size and with a different degree of bias. To validate our approach we compare it to a previously suggested linear model (LIN) [20] as well as a standard supervised multi-layer perceptron (MLP) for prediction of tissue of origin, sex and sample source. Importantly, we find that our DA network significantly outperforms the strongly supervised LIN model by up to 12.3% in prediction accuracy. We subsequently apply trained models to generate and make available new metadata for 8,495 unique SRA samples.

Methods

Data Acquisition

To train and test models we gathered data from three different sources (i.e. GTEx, TCGA and SRA), each with a different level of heterogeneity (Supplementary Figure 1). We measure data source heterogeneity by the number of unique dataset (or studies) in the source. Each dataset (or study) is believed to have a unique bias. Biases stem from the unique circumstances, protocols and reagents used as well as biological factors of the study [7, 8]. Here we define a dataset as all the RNA-seq samples from one study based on the assumption that they were obtained and processed under equal conditions. To avoid additional biases by the use of different bioinformatic alignment pipelines [27] all data was downloaded from recount2 (release 13.09.19, `https://jhubiostatistics.shinyapps.io/recount/`). Recount2 aggregates raw RNA-seq data from different sources and re-runs the data through the Rail-RNA alignment pipeline [28]. The RSE V2 files of all available RNA-seq projects (n=2,036) from recount2 were downloaded using the

recount R package (v 1.11.13). The downloaded data was separated into three different data sources according to their origin. Figure 1A gives a general overview of the data obtained, the pre-processing steps and data set preparation.

GTEx

The Genotype-Tissue Expression Project v6 (`https://www.gtexportal.org/`) comprises 9,662 samples from 554 healthy donors across 31 tissues. GTEx strives to build a highly homogeneous dataset with strict guidelines on donor selection, biopsy and sequencing methodology (more information at: `https://www.gtexportal.org/home/documentationPage`). We considered the GTEx data source to have a single dataset bias.

SRA

From the Sequencing Read Archive, a total of 2,034 studies containing a total of 49,657 samples were downloaded from recount2. Every SRA study was potentially processed at a different site by a different technician following different standards. In addition, the underlying biological condition of the samples is often unclear. We assume each study to have a unique dataset bias which makes the SRA a highly heterogeneous data source. In addition, data annotation is not standardized, resulting in sparse metadata with low fidelity.

TCGA

RNA-seq data for The Cancer Genome Atlas (`https://www.cancer.gov/about-nci/organization/ccg/research/structural-genomics/tcga`) was downloaded consisting of 11,284 samples spanning 26 tissues. While there are 740 samples of healthy donors across 20 tissues, more than 90% of the samples are tumor biopsies from different tissues and different stages of tumor progression. TCGA accepts sequence data from different locations using different sequencing technologies. Despite the high level of standardization and reliability of metadata information, heterogeneity is also inherent to the TCGA dataset due to the biological context (cancers, stages) albeit not as pronounced as in the SRA.

Preprocessing of SRA Data Source

In this study we focus on bulk mRNA-seq data, as it is by far the most frequent RNA type in either of the three data sources used. The following approaches were used to remove data from single-cell and small RNA-seq studies from further analysis: First, we identified small RNA-seq data on the basis of the total fraction of small RNA counts and protein coding RNAs. Specifically, we considered a subset of the Gencode gene types (i.e. protein_coding and processed_pseudogene vs. rRNA, miRNA, misc_RNA, snRNA and lincRNA). Every sample that had its maximum total count fraction not allocated to either protein_coding or processed_pseudogene was removed from further analysis (Supplementary Figure 2). Second, we removed single-cell RNA-seq studies by scanning titles and abstracts for variations of the words 'single cell' and manually validated and excluded the identified samples. In addition to this semi-automatic validation step we manually validated the 50 largest projects within the SRA data source and removed samples that did not qualify as bulk RNA-seq data. Most importantly, we noticed numerous technical replicates in the remaining SRA data. Using technical replicates to train and test a classification model inflates the reported metrics. Therefore, only samples with a unique experiment accession (SRX) were retained. From the 49,657 SRA samples downloaded initially, 29,685 samples and 1,833 unique studies passed our preprocessing steps.

Metadata

We considered three different phenotypes for expression based prediction. Explicitly, we predicted the tissue of origin of a biopsy (e.g. heart, lung, kidney, ovary), the patients' sex, and sample source (denoting whether the sample was from a patient biopsy or a lab grown cell line) (Figure 1A).

GTEx and TCGA

Tissue and sex annotation for GTEx were extracted from the official sample annotation table as provided by GTEx (GTEx_Data_V6_Annotations_SampleAttributesDS.txt, from `https://storage.googleapis.com/gtex_analysis_v6/annotations`). An annotation file for TCGA was provided by recount2. For tissue and sex annotation we took columns gdc_cases.project.primary_site and gdc_cases.demographic.gender respectively. Sample source was assumed to be of type biopsy for all GTEx (n=9,662) and TCGA (n=11,284) samples.

SRA

For the SRA samples we relied on normalized metadata provided by MetaSRA [14]. Available SRA identifiers were downloaded through the GUI

on http://metasra.biostat.wisc.edu by searching for all 31 GTEx tissues (site accessed on 11.09.2019). Supplementary Table 1 lists assumed mappings from GTEx tissue names to MetaSRA tissue names where no direct mapping was available. Of the 31 tissues available for GTEx we were able to identify samples for 26 in MetaSRA, resulting in 6,183 annotated SRA samples. Sample identifiers for sex were accessed through the same GUI by searching for male organism and female organism + Homo sapiens cell line which resulted in 3,240 annotated SRA samples. Sample source was determined using the sqlite file provided by MetaSRA (metasra.v1-5.sqlite, http://metasra.biostat.wisc.edu/download.html, column sample_type) resulting in 28,043 annotated samples across six sample source categories.

Tissue Label Harmonization

GTEx, TCGA and SRA have 17 common tissue types (Supplementary Figure 3). Bladder was removed due to its small sample size (GTEx n=11). We kept samples of comparable size in SRA (adrenal gland n=14, testis n=14, pancreas n=17 in the SRA training data), as the SRA training data is mainly used for bias injection, such that size was not considered an exclusion criterion. This resulted in 5,480, 8,624, and 3,252 tissue annotated samples across 16 tissues for GTEx, TCGA and SRA, respectively (Supplementary Tables 2 and 3).

Dimensionality Reduction and Normalization

The downloaded gene count table provided counts for 58,037 genes (Gencode v25, GRCh38, 07.2016). First standard log2 Transcript per Million (TPM) normalization was applied to normalize for gene length (Gencode v25, GRCh38, 07.2016) and library size. We next reduced the number of input features (genes), aiming to keep features that contain information and removing potentially uninformative features. First, all non-protein coding genes were removed, reducing the gene set by 65.5% to 19,950 genes. For sex classification, only protein coding genes on the X and Y chromosome (n=913) were selected. For retaining only genes that show significant dispersion across tissues, we computed the Gini coefficient [15, 29, 30] for all remaining genes across all GTEx samples. Housekeeping genes, for example, are known to be expressed similarly across tissues and would score a low Gini coefficient (i.e. high dispersion). Low and high cutoffs were applied during hyperparameter optimization. For tissue classification, genes with Gini coefficients g between 0.5 and 1 were retained, resulting in a features space of dimension d=6,974. For sex classification, genes with $0.4 < g < 0.7$ were used (d=190). Sample source classification included genes with $0.3 < g < 0.8$ (d=8,679) (Supplementary Table 2, list of input features in Supplementary Material).

Dataset Preparation

Phenotype Classification Experiments.

Tissue: To ensure that dataset biases are not shared between train and test sets, SRA data was always split on the study level. For tissue of origin prediction, the two largest SRA studies per class were put in the training set. This ensured maximal bias variability in the remaining test data, ensuring a realistic test score. Of the 178 SRA studies containing tissue annotated samples, 30 studies were selected for the training set (n=1,721) and 148 studies for the test set (n=1,531) (Supplementary Tables 2 and 3). **Sex**: We noticed SRA samples identified as female by MetaSRA to have a significant amount of reads mapped to chrY (Supplementary Figure 4). All samples labeled as female with a total normalized count $\geqslant 2$ and all samples labeled male with a total normalized count < 2 were removed. In total, 149 SRA studies contained samples annotated with male and or female by MetaSRA. These studies were combined into the training set (studies=73, n=2,017), and test set (studies=76, n=791) (Supplementary Tables 2 and 3). For model validation, GTEx was randomly split into training and test sets with an 80:20 ratio for both sex and tissue classification. **Sample Source**: A confidence cutoff of $\geqslant 0.7$ was applied (provided by MetaSRA), reducing the total amount of annotated samples for SRA from 23,651 to 17,343. MetaSRA provided six different types of sample source. The two largest classes, TISSUE and CELL LINE, were selected. In this study we renamed the MetaSRA label TISSUE to biopsy to not be confused with the phenotype tissue (e.g., heart, lung, skin). For each of the two selected categories we sorted all available studies by number of samples, placed the first third of studies into the training (studies=420, n=12,725), the second third into the test (studies=422, n=3,144) and the last third into the SRA validation set (studies=418, n=1,124) (Supplementary Tables 2 and 3). A list of the sample ids and corresponding labels is available in the Supplementary Material.

Metadata Annotation.
After determining the best model for each phenotype, we re-trained the models for automated metadata annotation. The same datasets as defined above were used for the sex metadata annotation. **Tissue**: We followed the same pipeline as described above. Samples from a tissue class other than the original 16 classes were pooled together into a 'catch-all' class, resulting in 17 classes. In total 44 SRA studies were selected for the training set (n=3,370) and 203 studies for the test set (n=2,813). **Sample Source**: Contrary to before, for metadata annotation we used all available classes in the SRA data source. All classes that are not of type biopsy were grouped into a single 'catch-all' class while the same cutoff as before was applied. The training set (n=16,463) is made up of 974 SRA studies and the test set (n=3,707) of 492 studies.

Multilayer Perceptron - MLP

MLPs use fully connected neural network layers to learn non-linear features from a raw input space [31] and constitute the most basic form of ANNs. All our ANN based models were developed and trained on tf.keras (Tensorflow 2.1). The hyperparameters for each prediction task were determined using exhaustive iterative random search (keras tuner 1.0.1) (Supplementary Table 4). In case of approximately equal accuracy on the validation set, the least complex model was chosen. A single hidden layer was used in each case with 128, 128 and 32 nodes for tissue, sample source, and sex prediction, respectively (Supplementary Table 5, Supplementary Figure 5). Each network was trained for 10 epochs with a batch size of 64. Performance was quantified by mean sample accuracy and mean class accuracy and subsequently used to benchmark our DA approach.

Domain Adaptation Model - DA

Many DA models correct bias between two domains, a source and a target domain. In biological research, however, one is often confronted with many small datasets, each potentially with its unique dataset bias. Therefore, we specifically designed our DA model to be able to learn from very few data by using a Siamese network architecture [32]. The Siamese network learns bias from pairs or triplets of training samples by exposing each sample in multiple relationships to the model. We distinguished three different types of input data for our model. The source domain is a large single-bias dataset used to learn the feature embedding for the classification task (in our case: GTEx). The bias domain contains labeled samples from multiple smaller datasets (in our case: SRA) each with its own bias. The target domain refers to unlabeled and biased datasets we want to classify (unlabeled SRA or TCGA data).

Model Architecture
Our DA architecture is based on the Siamese network architecture. A Siamese network usually shares the weights between two equal networks. Here, however, we do not use weight sharing. Weight sharing and other types of architecture did not prove to be applicable to this problem (see Methods section Other Models). It consists of three modules: A source mapper (SM) and bias mapper (BM) which correspond to the Siamese part of the model, as well as a classification layer (CL). These modules give rise to three different configurations, i.e. two training cycles and a prediction configuration (see Supplementary Figure 6 for a brief illustration). In the first training cycle, the source mapper (SM) and the classification layer (CL) are combined to form an MLP (Figure 2A). The task of the SM is to learn a mapping from the input space to an embedding space from which the CL can predict phenotype classes. The SM-CL module is trained with a batch size of 64 for 10 epochs. Because the SM-CL MLP is trained on a large single-bias dataset, it will likely overfit and thus not readily generalize to other datasets (Figure 2B). For the second training cycle, the SM and the CL are separated and their weights frozen. Frozen weights are not updated during the second training cycle. The bias mapper is created by copying the architecture and weights of the trained source mapper. SM and BM are trained on triplets drawn from the source and the bias domain (Figure 2C). Samples from the source domain are passed through the SM, samples from the bias domain through the BM at the same time. Each triplet is made up of an anchor (a) sampled from the bias domain, and a positive (p) and a negative sample (n) from the source domain. The anchor and the positive sample have equal class labels, whereas the negative sample is from a randomly selected different class. The triplet loss function [33] was used to optimize the model during training:

$$\mathcal{L} = max(d(a,p) - d(a,n) + m, 0)$$

Where $d(i,j)$ are the distances between the constricted embedding space of the SM and the bias mapping into that space of the BM on samples i and j. For improved

training time and robustness, our model is trained on semi-hard triplets [33]

$$d(a,p) < d(a,n) < d(a,p) + m$$

with a margin parameter m. The distances are defined as Euclidean distances in embedding space:

$$d(a,p) = \|\sigma(BM(a)) - \sigma(SM(p)))\|$$
$$d(a,n) = \|\sigma(BM(a)) - \sigma(SM(n)))\|$$

σ is the sigmoid activation function for the embedding vector. The SM-BM module was trained for 10 epochs with a batch size of 64. Hyperparameters were determined as described above (Supplementary Table 5, Supplementary Figure 5). As this training cycle proceeds, the BM learns to map its output onto the SM embedding space. After training, the bias mapper and the classification layer are combined to a BM-CL MLP and can be used for prediction of the target domain (Figure 2D). The source code as well as an example are available at: `github.com/imsb-uke/rna_augment`.

Linear Regression Model - LIN

We used the metadata prediction performance of the LIN model described in Ellis et al. [20] as a point of reference. The LIN model was optimized on the same data as all other models (see data section of methods). For each experimental setup, the following steps were conducted in R version 3.6.3 in order to build the corresponding phenotype predictor and evaluate its accuracy based on the test data:

1. calculating the coverage matrix for the training samples based on the regions reported in Ellis et al. [20] by employing the function 'coverage_matrix_bwtool' (R package recount.bwtool version 0.99.31).
2. building the model by running 'filter_regions' and 'build_predictor' (R package phenopredict version 0.99.0) with the same parameters used in Ellis et al. [20]
3. testing the model on the test samples with 'extract_data', 'predict_pheno', 'test_predictor' (R package phenopredict version 0.99.0)

Notably, our experiments differ from the original work [20] solely by applying additional preprocessing steps to the samples (see Methods), which may be responsible for observed small differences in performance. For implementation details and code examples for the before-mentioned functions, see the documentation (`http://rdrr.io/github/ShanEllis/phenopredict/`).

Nomenclature of Experiments

Each experiment was named after the model, the training and the test data used. The possible models are LIN (linear model [20]), MLP (multi-layer perceptron) and DA (novel domain adaptation approach). The data sources are named G (GTEx), T (TCGA) and S (SRA). If only the SRA training data is used (i.e. if the model is evaluated on the SRA test data) we write S_{small}. If the SRA train and test sets are combined for training we write S_{large}. For instance, an experiment using an MLP, trained on a mix of GTEx and SRA and evaluating on SRA data would be named MLP G+S_{small}-S.

Impact of Data Diversity and Quantity on Model Performance

To analyze the effect of training data diversity on prediction accuracy, the following experiments were designed. First, MLP S-S models for sample source prediction were trained with an increasing number of unique SRA studies in the training data, systematically increasing bias diversity. Only SRA studies containing > 100 samples for either class were considered. In order to control for training set size, each SRA study was subsampled to 50 samples before training. Six iterations of this training process were conducted starting with one study (i.e. one bias) per class (biopsy vs. cell line). At each step one additional SRA study per class was subsampled ending with six SRA biases and 350 samples in the training set per class. As a control experiment we chose the largest SRA study available for each class to create a training set with a single bias per class. Starting with 50 samples per class in six iterations we subsampled an additional 50 samples ending with 350 samples, thereby assessing the effect on performance that can be attributed to the dataset size. Subsampling and random selection of SRA studies were repeated 10 times with different seeds and each configuration was trained on 10 different seeds, yielding an estimate of uncertainty.

Test for Overfitting

MetaSRA provides labels for SRA data generated in an automated way. We have identified mislabeled samples for the sex phenotype (see Methods).

The following experiment was designed to test the ANN based model's susceptibility to overfitting on mislabeled training data. An MLP model was trained on GTEx data on four tissue classes (i.e., brain, esophagus, lung and skin). A range of fractions of the brain samples were randomly assigned to skin tissue (i.e., 0.01,0.025,0.05,0.1,0.20,0.5 and .8). The model was then trained on GTEx samples of the four classes, including the mislabeled brain samples. We tested the model's overfitting capabilities by letting it predict the label of the mislabelled brain samples. If the model overfits, these samples should be predicted to be from skin tissue. The same experiment was conducted for the sex phenotype by mislabeling male samples as female.

Metrics

We report micro and macro accuracy which are equivalent to mean sample accuracy (msa) and mean class accuracy (mca) respectively. Sample accuracy is a measure of absolute performance on the test data. It reports the fraction of correctly classified samples over all classes:

$$\mathrm{msa} = \frac{\sum_i^N \mathbb{1}_{y_i}(\hat{y}_i)}{N}$$

Where N is the number of samples, y the true label and $\hat{y}$ the predicted label, and $\mathbb{1}$ is the indicator function. Given the large class imbalance in some of our experiments, an increase in accuracy in a small class will not be captured by this metric. Average class accuracy, on the other hand, reports the average sample accuracy per class, weighing each class equally and thereby capturing local improvements of the models:

$$\mathrm{mca} = \frac{\sum_{j=1}^C \frac{1}{M_j} \sum_{i=1}^{M_j} \mathbb{1}_{y_{ij}}(\hat{y}_{ij})}{C}$$

Here, C is the number of classes, M_j is the number of samples for class j, and y_{ij} and $\hat{y}_{ij}$ are the true and predicted values, and $\mathbb{1}$ is the indicator function.

Statistical Tests

Accuracy distributions were tested for significance using the non-parametric Mann-Whitney-U-Test (scipy.stats.mannwhitneyu v 1.3.1).

Other Models

While developing our DA model we did a thorough literature research and implemented and tested multiple architectures and strategies. Here we give a brief overview of the models we found not suitable for the problem of bias invariant RNA-seq metadata annotation. The first strategy that has been tested was interpolation between source and target domain by training feature extractors on an increasing ratio of target to source domain data [34]. The second strategy was adversarial training by applying two loss functions. The first loss function forces the model to learn weights for the class prediction task, while the second forces the model to learn to ignore differences between the source and target domain [35]. We also implemented Tzeng et al.'s adaptation of this idea [36], proposing a model using a separate source and target encoder, using them as 'real' and generator input for a generative adversarial network [37] that is capable of ignoring bias. These models ultimately failed due to the hundreds of dataset biases in the SRA data and their relatively small sample size (data not shown). For the case of scarce target data an approach was previously proposed using Siamese networks [32, 38]. The trained model achieved an msa of 0.83 and mca of 0.79 for tissue classification on SRA data. The mca achieved is comparable to the results of the MLP model, however, the msa score is 6% lower than even the LIN model. The more challenging task of learning to map the bias embedding into the pre-learned class embedding, as presented in this paper, finally resulted in the desired outcome.

RESULTS

Experimental Setup

This study aims to find the best model for RNA-seq metadata annotation based on gene expression. Three different data sources were selected for which phenotype data was available (Figure 1A). Each of the three data sources comes with a different number of data set biases. Briefly, GTEx is a large homogeneous dataset containing healthy samples following a strict centralized standard protocol. TCGA contains pooled samples from different cancers, disease stages and sequencing centers. Our SRA data is made up of hundreds of individual studies following no centralized standard, containing the largest number of biases of all three data sources. Bias in a test dataset that has not been learned by a model can severely compromise performance. We hypothesized that

exposing classification models to a sufficient number of dataset biases will enable them to learn a generalized internal feature representation. Such a model would be able to classify data with previously unseen biases. To test and benchmark our models we selected the classification tasks of (1) tissue of origin of a given RNA-seq sample, (2) biopsy vs. cell-line origin of a sample (i.e. sample source), and (3) sample sex (Figure 1A).

Three different machine learning models were compared (Figure 1B). First, a fully connected ANN (MLP) was tested because of its capability to create novel latent features (see methods for model details). Second, we developed a domain adaptation (DA) approach (Figure 2), a subfield of machine learning dealing with dataset biases. Lastly, the LIN model trained on GTEx data, proposed in Ellis et al. [20], was used as the baseline for all tissue and sex classification experiments.

Models were trained on either GTEx or a mix of GTEx and SRA data and tested on TCGA and SRA data. Uncertainties for MLP and DA models were estimated from 10 training runs with different random seeds (Figure 1B).

Domain Adaptation Outperforms Other Models on Tissue Classification

We first tested the performance of the LIN, MLP, and DA algorithms to predict the tissue of origin on GTEx (n=5,480), TCGA (n=8,624), and SRA (train n=1,721, test n=1,531) datasets. A subset of 16 tissue labels was chosen that is common to all three data sources (see methods, Supplementary Figure 3, Supplementary Table 3). First, we conducted a single-bias experiment, i.e. MLP G-G (see Nomenclature of Experiments in methods). The nearly perfect score of mean sample accuracy (msa) 0.996 and mean class accuracy (mca) 0.99 (data not shown) confirmed that the MLP yielded highly accurate results when trained and tested on a single-bias dataset (for details on model training, validation, and testing see methods).

Prediction of SRA Tissue

Metadata prediction on SRA was the most challenging and interesting task due to the potentially large number of different biases in the data source. We re-trained and tested LIN G-S on our datasets and achieved a msa of 0.893 and a mca of 0.765 for the 16 tissues (Figure 3A). Of note is the significantly higher accuracy achieved with LIN G-S compared to the one reported by Ellis et al. [20] (0.519 msa). MLP G-S (msa: 0.872, mca: 0.77) had a higher mca but a lower msa than the corresponding LIN model (Figure 3A). In the next step we investigated the effect of adding bias to the training dataset on prediction performance. In particular, we first predicted SRA tissue from S_{small} data. MLP S_{small}-S (msa: 0.894, mca: 0.746) matched the base model's msa score but performed slightly worse using the mca metric. Similarly, the LIN S_{small}-S model matched the msa of LIN G-S but showed an increased performance for mca (msa: 0.893, mca: 0.795) Notably, by only using the small SRA training dataset, we lose the advantage of the large sample size of GTEx. Based on this we hypothesized that by combining SRA and GTEx in the training data, we may be able to leverage both sample size and diversity.

The LIN G+S_{small}-S model increased its msa to 0.908 and mca to 0.785 which in turn is 1% lower than the LIN S_{small}-S model. The two best performing models were MLP G+S_{small}-S and DA G+S_{small}-S, outperforming LIN G-S on msa by 2.5% and mca 5.5% (MLP G+S_{small}-S msa: 0.915, mca: 0.817 and DA G+S_{small}-S msa: 0.922, mca: 0.821). No significant difference in the mean performance was detected between these two models (msa p-val>0.02, mca p-val>0.4, Mann-Whitney). Crucially, however, DA G+S_{small}-S exhibited the lowest standard deviation (std=0.003 for msa and std=0.009 for mca) of all models tested (Supplementary Table 6). For this reason DA G+S_{small}-S was considered the best model for the prediction of tissue on the highly heterogeneous SRA test data. The best model increased the msa score by 3.6% compared to LIN G+S_{small}-S and mca by 5.6% compared to the baseline LIN S_{small}-S, the best performing linear models for the respective metrics.

Prediction of TCGA Tissue

Next, model performance on TCGA data was assessed (Figure 3B). The baseline model LIN G-T achieved msa 0.718 and mca 0.638. Applying the MLP model on the same data resulted in a drop of msa and mca of 2.4 and 3.3%, respectively (MLP G-T msa: 0.684, mca: 0.605). For TCGA tissue prediction we used S_{large} for training, essentially doubling the SRA training data (SRA train + SRA test set: n=3,252). LIN S_{large}-T improved accuracy by 6.6% for msa and 8.6% for mca to 0.784 and 0.724 respectively. In comparison, MLP S_{large}-T increased model performance by 11.4% to 0.832 (by 11.7% to 0.755) for msa (mca) with respect to LIN G-T. Combining GTEx and SRA training data reduced

LIN G+S_{large} performance to msa 0.725 and mca 0.651. The best accuracy was achieved by our MLP G+S_{large} (msa: 0.842, mca: 0.773) and DA G+S_{large} (msa: 0.875, mca: 0.813) models. The DA model had thus a 15.7% and 9.1% performance increase for msa compared to LIN G-T and LIN S_{large}-T, respectively. In addition to being the top performer, DA G+S_{large}-T also was the most robust model for this task, having the lowest variation in its results (std=0.004 for msa and std=0.006 for mca) (Supplementary Table 6).

We repeated the prediction for TCGA with the models trained for SRA tissue prediction (previous section), i.e. on S_{small}, which allows us to assess the influence of the amount of bias injection on model performance. Whereas the addition of more SRA data to the training data set had little influence on LIN models (except for a slight increase of ∼0.2% for G-S_{large}-T), both MLP and DA model accuracies improved significantly (by between 5 and 9%) upon addition of additional SRA data (Supplementary Table 6).

Notably, adding 5,480 GTEx training samples to MLP S_{small} (MLP-S_{small} → MLP G+S_{small}) increased msa from 0.748 to 0.764 and msa from 0.688 to 0.716 on the TCGA test set. On the other hand, adding 1,531 SRA samples (MLP-S_{small} → MLP S_{large}) increased msa to 0.832 and msa to 0.755, underlining our model's ability to incorporate multiple biases for better generalization (Supplementary Table 6).

Expression Based Prediction of Sample Source

SRA data stems from multiple different sources, from which we selected the two largest, namely either biopsy or (immortalized) cell lines, whereas GTEx and TCGA data are exclusively from biopsies. Starting from the hypothesis that fundamental differences do show on an expression level, we set out to train LIN and MLP models on SRA data to predict the sample source of SRA, GTEx and TCGA. Of note, while we were able to approximately reproduce the original results for LIN S_{small}-G and LIN S_{small}-S we were not able to do so for LIN S_{small}-T (msa: 0.998 reported in publication [20]). LIN S_{large}-G (msa/mca 0.951) did slightly better than MLP S_{large}-G (msa and mca of 0.943). MLP S_{large}-T achieved msa and mca 0.971, outperforming LIN S_{large}-T with (msa and mca of 0.882). MLP S_{small}-S achieved msa 0.95 and mca 0.941, outperforming LIN S_{small}-S with msa 0.89 and mca of 0.884 (Figure 3C).

Multi-Bias Data Enhances Tissue Classification on TCGA

For tissue classification on TCGA, mean class accuracy increased by 16.8% between MLP G-T and MLP G+S_{large}-T. This confirms our hypothesis that the homogeneity of the GTEx data did not allow the MLP G-T model to generalize to TCGA data, while the addition of SRA training data in MLP G+S_{large}-T resulted in a model with significantly improved generalization. To further investigate this result, we took a closer look at the per class accuracy for the TCGA tissue prediction (Figure 3D, Supplementary Figure 7). MLP G-T was unable to predict samples for three tissues, namely bone marrow (msa: 0.08), ovary (msa: 0.02) and uterus (msa: 0.07), whereas all our other models achieved accuracies between 0.7 and 1.0 on these tissues. Adding SRA data to the training set enabled the model to achieve per tissue sample accuracy of 1.00, 0.704 and 0.67 for bone marrow, ovary and uterus, respectively. We used principal component analysis (PCA) to visualize the dataset bias for ovary tissue (Figure 3E). Interestingly, the GTEx-ovary and TCGA-ovary data points show little overlap in the PCA plot, while the SRA-ovary data overlaps with GTEx- as well as TCGA-ovary data, forming a 'bridge'.

Linear Model Sufficient For Sex Classification

For sex classification, only genes on the X and Y chromosome were used as input features (d=190). We first tested the trivial case MLP G-G by splitting GTEx into training and test sets, achieving sample and class accuracy of 0.995 (data not shown).

Prediction of TCGA Sex

Sex phenotype prediction on TCGA data was the only task where the linear model outperformed the ANN models. The baseline LIN G-T as well as the other linear models LIN S_{large}-T and LIN G+S_{large}-T achieved almost perfect accuracy on the TCGA data (msa/mca 0.989 for LIN G-T and LIN G+S_{large}-T, msa 0.988 and mca 0.987 for LIN S_{large}-T). Our best model, based on the data annotation provided by MetaSRA, was MLP G+S_{large}-T with msa 0.964 and mca 0.962 (Supplementary Figure 8).

Prediction of SRA Sex

All linear models for the prediction of sex for SRA data achieved an accuracy (msa: 0.98 and mca: 0.98 for LIN G-S and LIN G+S_{small}-S, msa: 0.979 and mca: 0.979 for LIN S_{small}-S). This result is significantly

better than what was previously reported (msa: 0.863 [20]). The MLP G-S model (msa: 0.971 and mca: 0.979) did, on average, perform worse than all the linear models. While adding SRA data to the training set did not improve the LIN model, it increased the performance of MLP and DA models. DA G+S_{small}-S (msa: 0.99 and mca: 0.987), MLP S_{small}-S (msa: 0.994 and mca: 0.994) and MLP G+S_{small}-S (msa: 0.993 and mca: 0.992). Results are shown in Supplementary Figure 8.

According to MetaSRA all our training and testing data for sex prediction on SRA stem from patient biopsies. However, at least two of the largest misclassified SRA studies in the test set are clearly cultured cell lines. For example, SRP056612 is a study on the effect of the MERS coronavirus on cultured kidney and lung cells [39] and SRP045611 is a study involving HEK cells, which lack the Y chromosome but are annotated as male by MetaSRA [40]. These are two examples of errors in the MetaSRA. Clearly, mislabeled data can compromise classifier accuracy, either by providing the wrong ground truth for training or by reporting the false label at the point of prediction. As described in the methods section, obviously mislabeled samples have been removed.

Training Data Diversity Outweighs Quantity

Our experiments on phenotype classification seem to indicate that increased training data diversity might enhance classification performance. To learn more about the relationship between the amount of training data and model performance, MLP G-S was trained on an increasingly large subset of the GTEx training data for tissue classification. We observed a limited effect on model performance with increased training dataset size. The msa reaches its peak with one third of the available training data, while the mca saturates at about half of the available training data (Supplementary Figure 9).

To test the effect of bias in the training data, an MLP S_{small}-S for sample source classification was trained on an increasing number of biases in the training set. As a control experiment an MLP was trained with the same amount of data but drawn from a single-bias source. We observed a positive correlation between msa and the number of biases in the training set (Figure 4A). Contrary to that, increasing the number of training samples by the same amount but from a single-bias source did not lead to better model performance (Figure 4B), validating our assumptions. Both experiments support our assumption that ANN based models can integrate different biases in the training set and translate them into better model performance compared to other methods.

ANN Models Can Correct Mislabeling in MetaSRA

Given the difficulties with metadata standards in SRA data, mislabeling in MetaSRA is to be expected. To understand if and when ANN models would overfit on mislabelled MetaSRA data, we trained an MLP on partially mislabeled samples (see Methods). Supplementary Figure 10 shows that the MLP model correctly predicts brain samples, even if they were presented as skin samples during model training. A decrease of this accuracy was observed if more than 20% of all brain samples were mislabeled as skin. A similar observation was made for the sex phenotype (Supplementary Figure 10). We concluded that our models are robust if less than 20% mislabelled data is present during training. More importantly, these models can be used to correct mislabeled MetaSRA data.

In the specific case of sex classification, the MLP G+S was used to predict the true corrected label for the SEX samples that were removed from training due to low sex-chromosome counts (see Methods). For 82% of the 132 filtered samples, the MLP model predicted the opposite of the presumably wrong MetaSRA labels. However, our MLP model was able to confirm the MetaSRA label for 24 samples. These samples had a mean chrY count sum of 2.4 (i.e. close to the cutoff value). Manual confirmation revealed a high model accuracy. For example, SRR1164833, SRR1164787 and SRR1164842 are samples from a prostate cancer study labeled as MALE by MetaSRA. Our MLP model correctly classified these samples despite the fact that their chrY total sum count was between 0.4 and 1.4. On the other hand, SRR16076 54 / 56 / 61 / 62 / 64 / 65 / 70 / 71 are annotated as FEMALE by MetaSRA and the MLP but had a chrY total sum count of 2-5.3. We see the correct classification of these borderline cases as further evidence that no overfitting is taking place.

A list of all SRA samples for which the MetaSRA labels and the predicted labels mismatched is available in the Supplementary Material.

Prediction and Availability of Novel Metadata

We have used our best models to predict high-quality metadata for published SRA samples lacking information on tissue, sex, or sample source.

Prediction of sex is straightforward because our models were trained on all possible biological categories. For tissue and sample source, however, our models were trained on a subset of all potential classes in the unlabeled data. If, for example, we try to label a sample of a tissue type unknown by the model, the model will force one of the learned classes onto that sample. To deal with this in the best possible way for sample source classification, we modified the classification task into one vs. all. Specifically, we first trained a new MLP model to identify the sample source biopsy vs. all other sample sources available in the SRA data as defined by MetaSRA. This model (i.e. MLP S_{small}-S) achieved msa 0.947 and mca 0.93 on a test set (data not shown) and MLP S_{large} was subsequently used to identify all of our yet unannotated SRA samples of source type biopsy. At a probability cutoff of 0.5 we identified 1,072 new SRA samples as originating from a biopsy.

Second, we extended the tissue classification task to 17 classes by adding a 'catch-all' class. To this end, we extended the training data to all GTEx (n=9,366) and SRA (n=6,183) data with tissue labels and assigned the placeholder class for every sample that did not belong to the original set of 16 tissues. That way we ensure that the learned model will not force known classes on every tissue type. With this approach, the DA G+S_{small} model achieved msa 0.912 and mca 0.787 (data not shown). Training and test datasets were subsequently combined to train DA G+S_{large} for annotation prediction of unlabeled SRA samples. We predicted the tissue of origin for all SRA samples of source type biopsy for which no entry on MetaSRA was available (n=2,818).

Third, 8,495 SRA biopsy samples with missing sex information were predicted using MLP G+S_{large}. Supplementary Figure 11 shows the true positive rate for each phenotype and each class on the test set. We provide this information such that users can make their own decision on probability cutoffs applied to each class. We provide the full list of all classified SRA samples as well as the probability output of the classifier in the Supplementary Material.

Discussion

We developed a novel deep-learning based domain adaptation approach for automated bias invariant metadata annotation. To the best of our knowledge this is the first time domain adaptation has been applied to this problem. We were able to outperform the current best model [20] on tissue prediction by 2.9% for SRA and 15.7% for TCGA data on mean sample accuracy. We can confirm, as was previously reported [17], that ANNs trained on single-bias training data do not perform better than linear models. Given multi-bias training data, however, we showed that MLPs, and especially our DA algorithm, have an advantage over standard machine learning approaches (e.g. linear regression). Our current models help researchers to verify the sex, tissue and sample type of RNA-seq samples in the presence of bias. This metadata information is currently rarely given for datasets downloaded from the SRA but can be of crucial importance.

The main strength of our method is its ability to incorporate dataset bias from datasets with only a few samples by applying a Siamese network-like architecture. The model learns to ignore bias by repeated exposure to (a few) samples in (many) different contexts, i.e. as triplets. In addition, it does not rely on feature selection but uses normalized gene count tables and lets the network learn which features carry important information.

Different types of experiments showed the importance of training models on a multi-bias dataset. First, we showed for every phenotype classification that models which had SRA samples included in the training data performed better than models trained only on GTEx data. For tissue classification, we further showed that the effect of adding SRA samples to the training data outweighs adding 3.2x as much GTEx data (MLP S_{small} →MLP S_{large} vs. MLP S_{small} →MLP G-S_{small}). Second, for SRA tissue classification we showed that there is a limit of accuracy that can be achieved irrespective of the size of the training set. Our experiment showed that peak accuracy is already reached by using 50% of the available data. Lastly, for sample source classification, we directly compared the relationship between the number of biases in the training data, the number of samples and the model performance. We found a positive correlation between the diversity of the training data and the accuracy achieved by that model.

We showed that our models are robust to overfitting, if up to 20% of the training samples per class are mislabeled. Our models are able to predict the correct class of a sample, even if the sample was mislabeled during model training. This property of our models was exploited for the correction of wrongly annotated metadata in the MetaSRA and made publicly available.

Lastly, we generated novel metadata for SRA samples using our best performing models, adding over 10,000 new metadata entries for 8,495 SRA samples. The newly generated metadata is now publicly available and can be used for future research. We see this as a first and important step in the general direction of an effort to make publicly available data more accessible and reusable in an automated way.

We observed some limitations to our DA approach. Our experiments showed that the DA model does not perform as well as the MLP for classification tasks with a low number of classes (e.g. sex). At least for the TCGA tissue classification, it seems that a minimum of about 8 classes is needed for the DA model to be able to unfold its full potential consistently. Our experiments indicate that the difference between DA and MLP performance will keep increasing, in favor of the DA model, the more classes we add (Supplementary Figure 12). Adding more tissue classes to our model is an important next step. Another limitation is posed by the need for labeled data to train the bias mapper.

Whereas currently the scope of our predictive models has been limited by the availability of data (e.g. intersecting tissue types between datasets, limited size of datasets), the approach is ready to incorporate more data, biases, classes, and more phenotypes, and there is reason to believe that this will confer increased performance of ANN based models, in particular DA models. At the same time, automated annotation ensures that the vast amount of data, currently lying idle in online repositories and institutional data centers, can indeed be leveraged. We believe that this synergy is capable of producing a large and comprehensive body of annotated biological data that will boost knowledge discovery for biomedical research.

Availability of supporting source code and requirements

Project name: Bias invariant RNA-seq metadata annotation
Project home page: https://github.com/imsb-uke/rna_augment
Operating system(s): Platform independent
Programming language: Python
Other requirements: TensorFlow 2.1
License: MIT

Acknowledgments

We would like to thank the members of the Institute for Medical Systems Biology for helpful discussions and comments. In particular, we would like to thank Sergio Oller for help with IT problems and support. In addition, we would like the ZMNH IT for their continuous support. We would like to acknowledge TCGA Research Network (`https://www.cancer.gov/tcga`) for data provision.

Funding

This work was supported by the DFG grants CRU 306 P-C, 296 P8, and CRC 1286 Z2 for SH, HW, and SB, respectively.

Conflict of Interest

The authors declare no conflict of interest.

References

1. R. Hrdlickova, M. Toloue, and B. Tian. Rna-seq methods for transcriptome analysis. *Wiley Interdiscip Rev: RNA*, 8(1):e1364, 2017.
2. S. Goodwin, J.D. McPherson, and W.R. McCombie. Coming of age: ten years of next-generation sequencing technologies. *Nat. Rev. Genet.*, 17(6):33, 2016.
3. Z. Wang, M. Gerstein, and M. Snyder. Rna-seq: a revolutionary tool for transcriptomics. *Nat. Rev. Genet.*, 10(1):57–63, 2009.
4. V. Costa, M. Aprile, R. Esposito, and A. Ciccodicola. Rna-seq and human complex diseases: recent accomplishments and future perspectives. *Eur. J. Hum. Genet.*, 21(2):134–142, 2013.
5. P.A.C. 't Hoen, M.R. Friedländer, J. Almlöf, M. Sammeth, I. Pulyakhina, S.Y. Anvar, J.F. Laros, H.P. Buermans, O. Karlberg, M. Brännvall, and J.T den Dunnen. Reproducibility of high-throughput mrna and small rna sequencing across laboratories. *Nat. Biotechnol.*, 31(11):1015–1022, 2013.
6. S. Li, P.P. Łabaj, P. Zumbo, P. Sykacek, W. Shi, L. Shi, J. Phan, P.Y. Wu, M. Wang, C. Wang, and D. Thierry-Mieg. Detecting and correcting systematic variation in large-scale rna sequencing data. *Nat. Biotechnol.*, 32(9):888–895, 2014.

7. A. Torralba and A.A. Efros. Unbiased look at dataset bias. In *CVPR 2011*, pages 1521–1528. IEEE, 2011.
8. M.A. Taub, H.C. Bravo, and R.A. Irizarry. Overcoming bias and systematic errors in next generation sequencing data. *Genome Med*, 2(12):87, 2010.
9. Z.D. Stephens, S.Y. Lee, F. Faghri, R.H. Campbell, C. Zhai, M.J. Efron, R. Iyer, M.C. Schatz, S. Sinha, and G.E. Robinson. Big data: astronomical or genomical? *PLoS Biol.*, 13(7):p.e1002195, 2015.
10. J. Lonsdale, J. Thomas, M. Salvatore, R. Phillips, E. Lo, S. Shad, R. Hasz, G. Walters, F. Garcia, N. Young, and B. Foster. The genotype-tissue expression (gtex) project. *Nat. Genet.*, 45(6):580–585, 2013.
11. R. Leinonen, H. Sugawara, M. Shumway, and International Nucleotide Sequence Database Collaboration. The sequence read archive. *Nucleic Acids Res.*, 39(suppl1):D19–D21, 2010.
12. N.H. Shah, C. Jonquet, A.P. Chiang, A.J. Butte, R. Chen, and M.A. Musen. Ontology-driven indexing of public datasets for translational bioinformatics. *BMC Bioinformatics*, 10(S2):S1, 2009.
13. E. Galeota and M. Pelizzola. Ontology-based annotations and semantic relations in large-scale (epi) genomics data. *Brief. Bioinformatics*, 18(3):403–412, 2017.
14. M.N. Bernstein, A. Doan, and C.N. Dewey. Metasra: normalized human sample-specific metadata for the sequence read archive. *Bioinformatics*, 33(18):2914–2923, 2017.
15. K. Hatje, R.U. Rahman, R.O. Vidal, D. Simm, B. Hammesfahr, V. Bansal, A. Rajput, M.E. Mickael, T. Sun, S. Bonn, and M. Kollmar. The landscape of human mutually exclusive splicing. *Mol. Syst. Biol.*, 13(12):959, 2017.
16. M.N. Bernstein, Z. Ma, M. Gleicher, and C.N. Dewey. Cello: Comprehensive and hierarchical cell type classification of human cells with the cell ontology. *BioRxiv*, page 634097, 2020.
17. A.M. Smith, J.R. Walsh, J. Long, C.B. Davis, P. Henstock, M.R. Hodge, M. Maciejewski, X.J. Mu, S. Ra, S. Zhao, and D. Ziemek. Standard machine learning approaches outperform deep representation learning on phenotype prediction from transcriptomics data. *BMC bioinformatics*, 21(1):1–18, 2020.
18. K. Zarringhalam, D. Degras, C. Brockel, and D. Ziemek. Robust phenotype prediction from gene expression data using differential shrinkage of co-regulated genes. *Sci. Rep.*, 8(1):1–10, 2018.
19. T. Kang, W. Ding, L. Zhang, D. Ziemek, and K. Zarringhalam. A biological network-based regularized artificial neural network model for robust phenotype prediction from gene expression data. *BMC bioinformatics*, 18(1):656, 2017.
20. S.E. Ellis, L. Collado-Torres, A. Jaffe, and J.T. Leek. Improving the value of public rna-seq expression data by phenotype prediction. *Nucleic Acids Res.*, 46(9):e54–e54, 2018.
21. M. Marouf, P. Machart, V. Bansal, C. Kilian, D.S. Magruder, C.F. Krebs, and S. Bonn. Realistic in silico generation and augmentation of single-cell rna-seq data using generative adversarial networks. *Nat. Commun.*, 11(1):1–12, 2020.
22. K. Menden, M. Marouf, S. Oller, A. Dalmia, D.S. Magruder, K. Kloiber, P. Heutink, and S. Bonn. Deep learning–based cell composition analysis from tissue expression profiles. *Sci. Adv.*, 6(30):eaba2619, 2020.
23. P. Mamoshina, A. Vieira, E. Putin, and A. Zhavoronkov. Applications of deep learning in biomedicine. *Mol. Pharm.*, 13(5):1445–1454, 2016.
24. M. Wainberg, D. Merico, A. Delong, and B.J. Frey. Deep learning in biomedicine. *Nat. Biotechnol.*, 36(9):829–838, 2018.
25. G. Csurka. Domain adaptation for visual applications: A comprehensive survey. *arXiv*, page 1702.05374, 2017.
26. T. Tommasi, N. Patricia, B. Caputo, and T. Tuytelaars. A deeper look at dataset bias. *arXiv*, page 1505.01257, 2017.
27. S. Arora, S.S. Pattwell, E.C. Holland, and H. Bolouri. Variability in estimated gene expression among commonly used rna-seq pipelines. *Sci. Rep.*, 10(1):1–9, 2020.
28. A. Nellore, L. Collado-Torres, A.E. Jaffe, J. Alquicira-Hernández, C. Wilks, J. Pritt, J. Morton, J.T. Leek, and B. Langmead. Rail-rna: scalable analysis of rna-seq splicing and coverage. *Bioinformatics*, 33(24):4033–4040, 2017.
29. L. Ceriani and P. Verme. The origins of the gini index: extracts from variabilità e mutabilità (1912) by corrado gini. *J. Econ. Inequal.*, 10(2):421–443, 2012.
30. J.D. Zhang, K. Hatje, G. Sturm, C. Broger, M. Ebeling, M. Burtin, F. Terzi, S.I. Pomposiello, and L. Badi. Detect tissue heterogeneity in gene expression data with bioqc. *BMC Genomics*, 18(1):1–9, 2017.

31. Y. LeCun, Y. Bengio, and G. Hinton. Deep learning. *Nature*, 521(7553):436–444, 2015.
32. S. Chopra, R. Hadsell, and Y. LeCun. Learning a similarity metric discriminatively, with application to face verification. In *Computer Vision and Pattern Recognition, 2005 (CVPR 2005)*, page 539–546. IEEE, 2005.
33. F. Schroff, D. Kalenichenko, and J. Philbin. Facenet: A unified embedding for face recognition and clustering. In *Proceedings of the IEEE conference on computer vision and pattern recognition*, pages 815–823, 2015.
34. S. Chopra, S. Balakrishnan, and R. Gopalan. Dlid: Deep learning for domain adaptation by interpolating between domains. In *ICML workshop on challenges in representation learning*, volume 2, 2013.
35. Y. Ganin, E. Ustinova, H. Ajakan, P. Germain, H. Larochelle, F. Laviolette, M. Marchand, and V. Lempitsky. Domain-adversarial training of neural networks. *J. Mach. Learn. Res*, 17(1):2096–2030, 2016.
36. E. Tzeng, J. Hoffman, K. Saenko, and T. Darrell. Adversarial discriminative domain adaptation. In *Proceedings of the IEEE conference on computer vision and pattern recognition*, pages 7167–7176. IEEE, 2017.
37. I. Goodfellow, J. Pouget-Abadie, M. Mirza, B. Xu, D. Warde-Farleyn, S. Ozair, A. Courville, and Y. Bengio. Generative adversarial nets. In *Advances in Neural Information Processing Systems*, pages 2672–2680, 2014.
38. S. Motiian, Q. Jones, S. Iranmanesh, and G. Doretto. Few-shot adversarial domain adaptation. In *Advances in Neural Information Processing Systems*, pages 6670–6680, 2017.
39. M.L. Yeung, Y. Yao, L. Jia, J.F. Chan, K.H. Chan, K.F. Cheung, H. Chen, V.K. Poon, A.K. Tsang, K.K. To, and M.K. Yiu. Mers coronavirus induces apoptosis in kidney and lung by upregulating smad7 and fgf2. *Nat. Microbiol.*, 1(3):1–8, 2016.
40. Y. Kravtsova-Ivantsiv, I. Shomer, V. Cohen-Kaplan, B. Snijder, G. Superti-Furga, H. Gonen, T. Sommer, T. Ziv, A. Admon, I. Naroditsky, and M. Jbara. Kpc1-mediated ubiquitination and proteasomal processing of nf-kb1 p105 to p50 restricts tumor growth. *Cell*, 161(2):333–347, 2015.

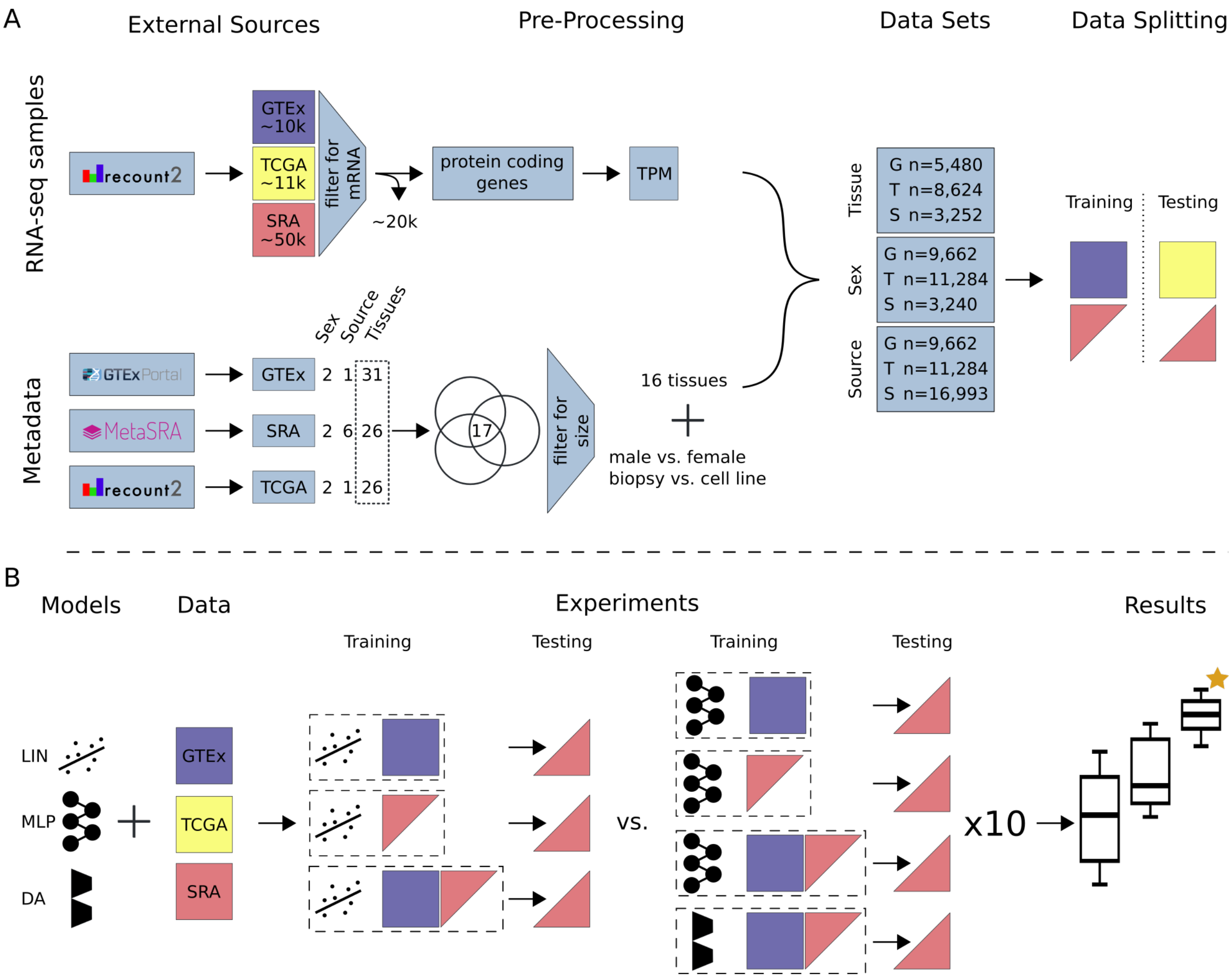

Fig. 1. Study Overview. (A) All data available on recount2 was downloaded and split into three data sources: (i) GTEx, (ii) TCGA and (iii) SRA. Single-cell and small RNA samples as well as technical replicates were removed from the SRA data. Protein coding genes were selected from the gene count tables and TPM normalized. Metadata for tissue of origin (e.g. heart), source (e.g. biopsy) and sex phenotype was collected, if available. A subset of 17 tissues (common to GTEx, TCGA and SRA) was selected and filtered for class size, resulting in 16 tissue classes. For sample source the two largest classes in SRA were selected. Samples were subsequently annotated and training and testing data sets were created. GTEx was only used for model training unless stated otherwise. TCGA was only used for model testing. SRA was split such that samples from one study are exclusively in the train or test set. (B) We compare three models: LIN (linear model), MLP (multi-layer perceptron) and DA (novel domain adaptation algorithm). Experiments are different combinations of models and data sources. Here, an exhaustive list of experiments for tissue and sex classification tested on SRA data is depicted. Each configuration (dashed box) is made up of a model and training data. The previously published LIN model served as a benchmark for our MLP and DA model. Each model configuration was trained 10 times with different seeds to give an estimation of uncertainty. The best model (orange star) was chosen by comparing average performance across all seeds. After determination of the best model, all available data was used for model training. Previously unlabeled SRA data was automatically annotated with the appropriate metadata. A list of all new metadata can be downloaded with the Supplementary Material.

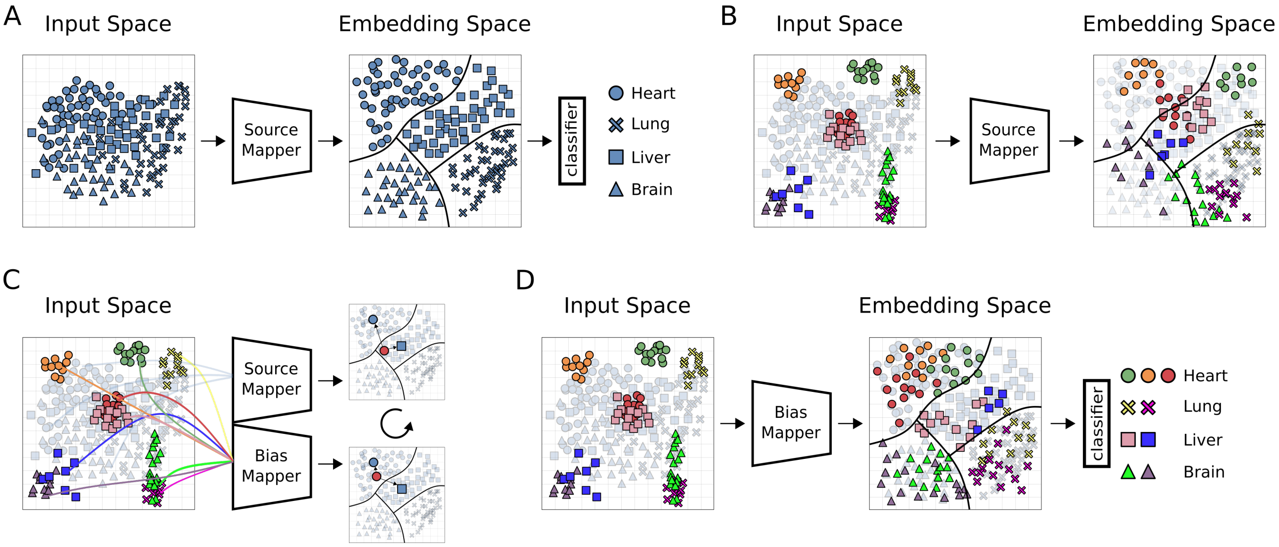

Fig. 2. Overview Domain Adaptation Model. Illustration of our DA model architecture and training. Shapes of (hypothetical) data points represent classes, colors are datasets with unique biases. Source Mapper (SM), Bias Mapper (BM) and classifier layer (CL) are ANN modules. (A) First training cycle: The SM is trained on a single bias dataset, the source domain (SD). In this step, the SM learns a feature embedding. The CL learns how to partition this embedding space into classifiable regions and draws decision boundaries (black lines). B) For biased test data (colored sample data points), the same classes may occupy distinct regions in input space. In this case, the source mapper may not be able to map the samples to the correct region of embedding space, compromising classification performance of the CL. C) In order to learn the mapping of different biases to the embedding learned in A, a bias mapper (BM) is created by copying the SM, and trained weights of the SM are fixed. In this second training cycle, triplets of samples are passed through the SM-BM configuration, consisting of an anchor from the bias domain and two samples from the source domain, one of them with a matching label. The triplet loss function is defined to minimize distance of like labels in embedding space and to maximize distance of opposite labels. This process is repeated until the SM has learned to map all known biases into the previously learned embedding space. (D) The BM is now able to map data points from previously unseen datasets into the embedding space where the CL can classify them.

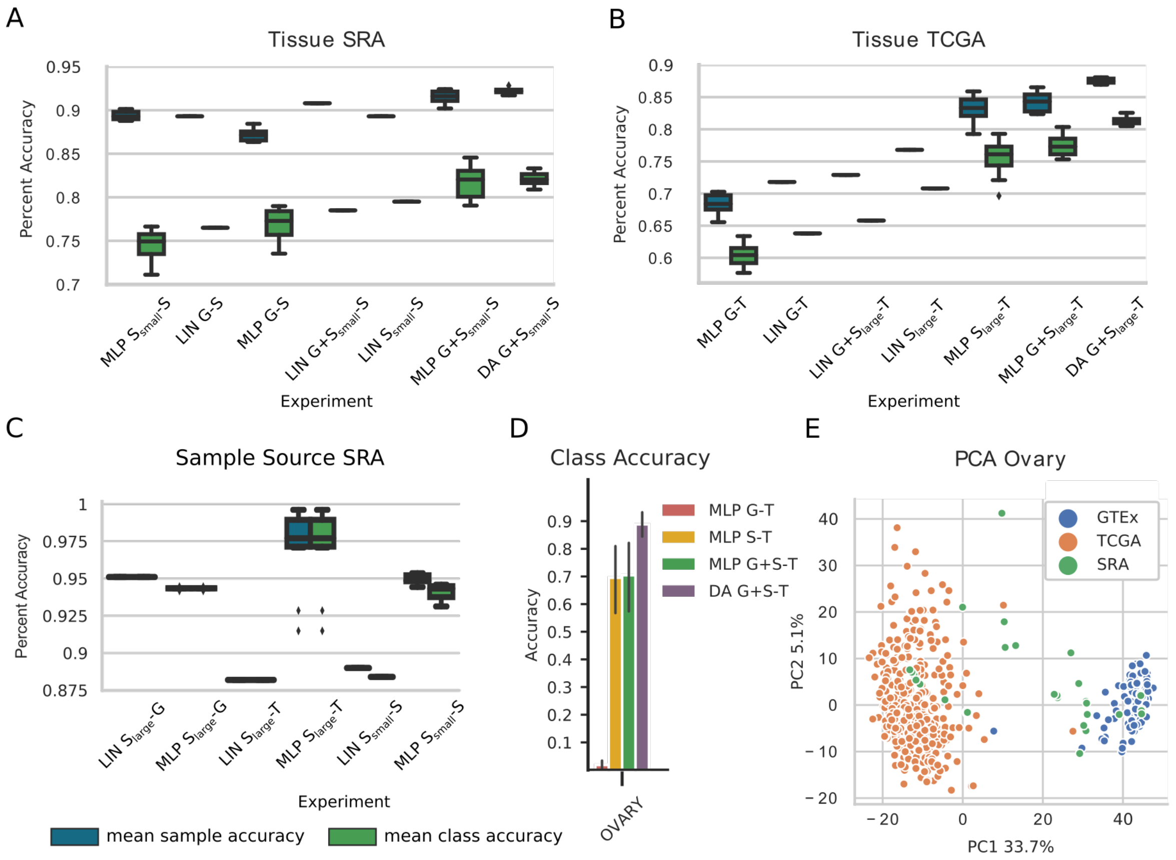

Fig. 3. Phenotype Prediction Results for A, B) prediction of tissue of origin on SRA and TCGA (16 classes) and C) prediction of sample source on SRA (2 classes). Indices 'small' and 'large' refer to the different size of SRA training data used due to splits of the data set in SRA prediction. Box plots represent model uncertainty of ANN based models, estimated from training with different random seeds (n=10). Mean sample accuracy and mean class accuracy were calculated for each seed. For panel A-C) LIN G-X is the baseline proposed in [20]. D) Accuracy of each ANN model predicting ovary tissue on the TCGA data source and E) a PCA plot of the gene expression values for the ovary tissue samples. A domain shift (i.e. bias) is clearly visible between GTEx and TCGA, leading to the poor performance of MLP G-T on ovary. SRA data in the training set helps to establish a good accuracy. LIN=linear regression, MLP=multilayer perceptron, DA=domain adaptation, G=GTEx, T=TCGA, S=SRA.

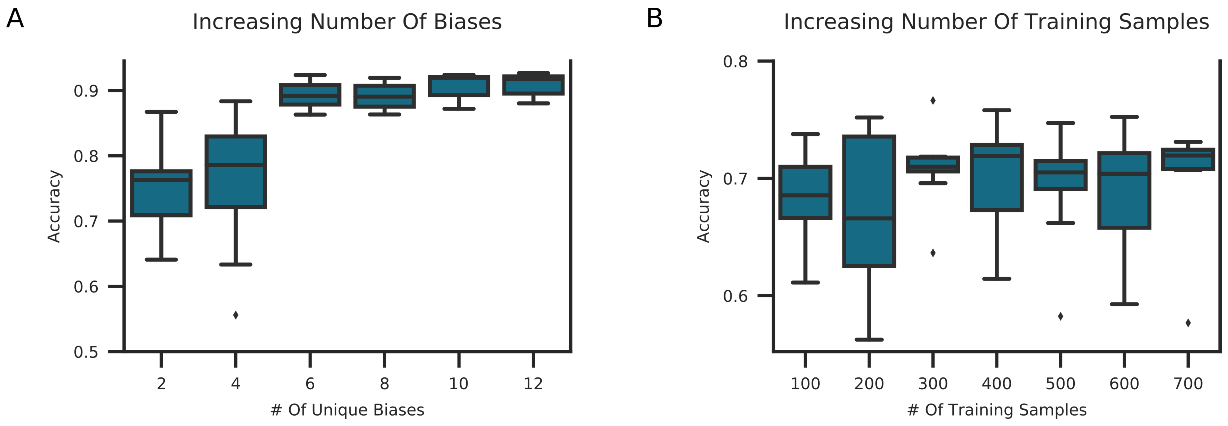

Fig. 4. Increasing Bias Vs. Increasing Sample Size in Training Data. A) A MLP S_{small} for sample source prediction on SRA data was trained by randomly sampling an increasing number of SRA studies per class. Each study was subsampled to 50 samples. Studies were drawn from all SRA studies with n > 100 for either sample source tissue or cell line. B) To differentiate the effect of increased bias vs. increased sample size, the same model was trained by randomly subsampling the largest available SRA study per class. At each step an additional 50 samples were added to the training set per class. Models were run with 10 different seeds and the mean sample accuracy was computed. Box plots are produced by 10 random sampling iterations. We observe a positive correlation between training data diversity and accuracy.

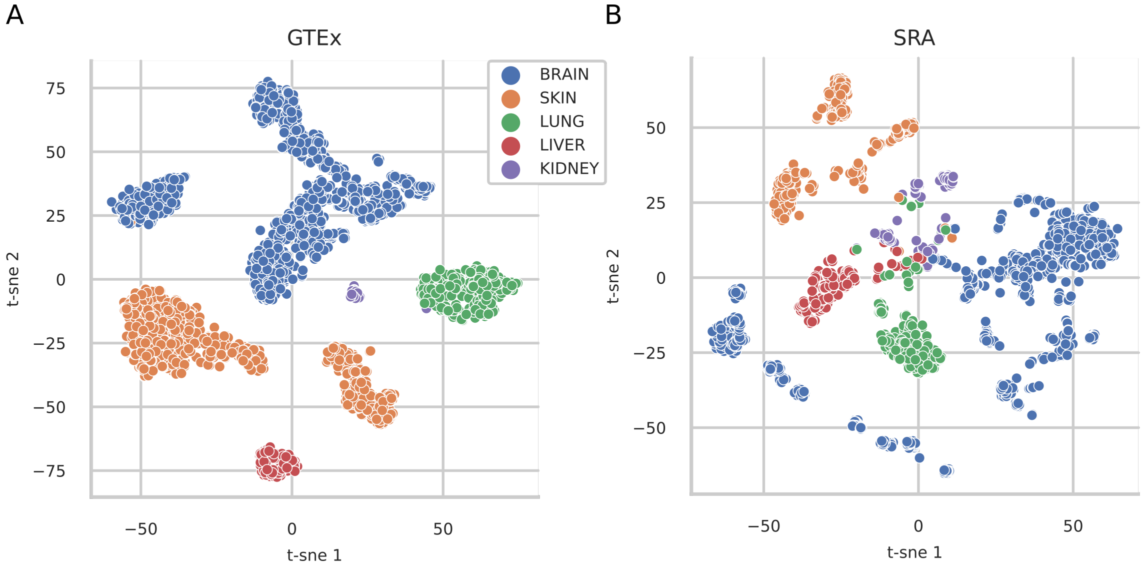

Fig. S1. Visualizing Data Set Bias. GTEx is a single-study data source, while SRA is a multi-study data source. A) T-SNE plot of gene expression values of GTEx and B) SRA samples, belonging to five different tissues. The GTEx data is more coherently clustered compared to the SRA data. The individual studies in the SRA data appear to form less homogeneous clusters, indicating a larger within-variance in the data source.

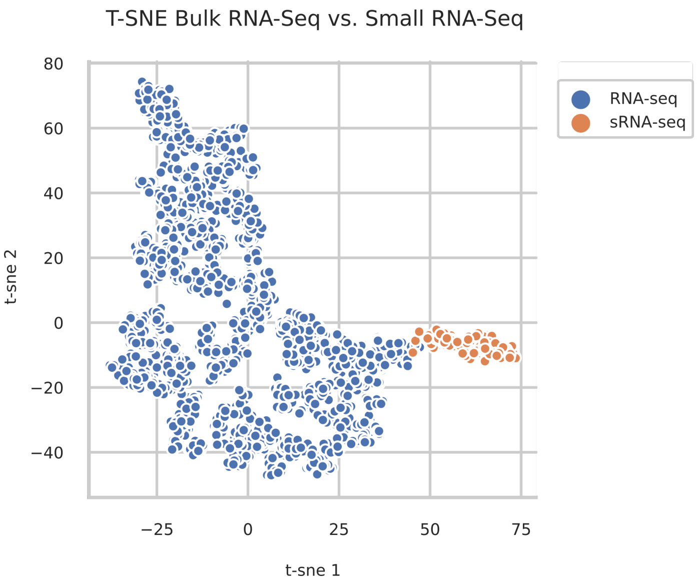

Fig. S2. T-SNE on Fraction of Total Gene Count Per Gene Type. The fraction of the total log TPM normalized counts per gene type was calculated for all types that can be associated with mRNA or small RNA. T-SNE was applied on the resulting vectors of fraction per gene type. Samples with their maximum fraction in a gene type belonging to a small RNA category were labeled orange, else blue. The scatter plot shows samples labeled as small RNA-seq all cluster together, suggesting a valid approach.

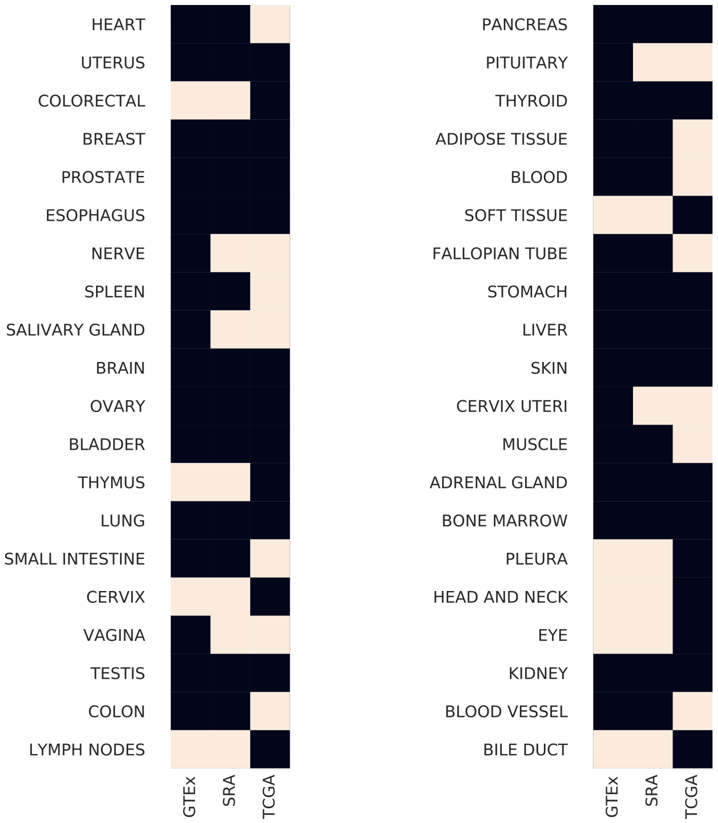

Fig. S3. Tissue Label Overlap Between GTEx, TCGA and SRA. GTEx v6 provides samples for 31 tissues and TCGA for 26. MetaSRA provided labels for 26 of the 31 GTEx tissues. This figure depicts the 40 tissues which form the union between the three data sources, a black square indicating that a tissue is present in the respective dataset. 17 Tissues are shared between GTEx, TCGA and SRA, 16 of which were used for tissue prediction.

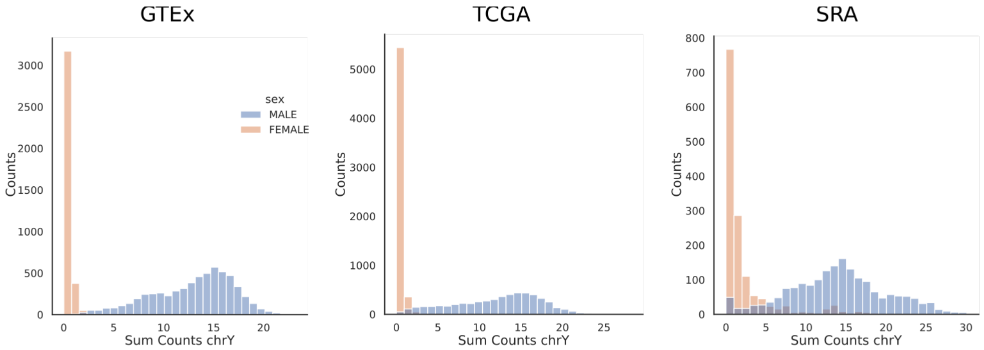

Fig. S4. Misclassification in MetaSRA. Histogram of the total sum of normalized counts mapped to the chrY for GTEx, TCGA and SRA. Male and female clearly overlap in SRA, indicating mislabeling by MetaSRA.

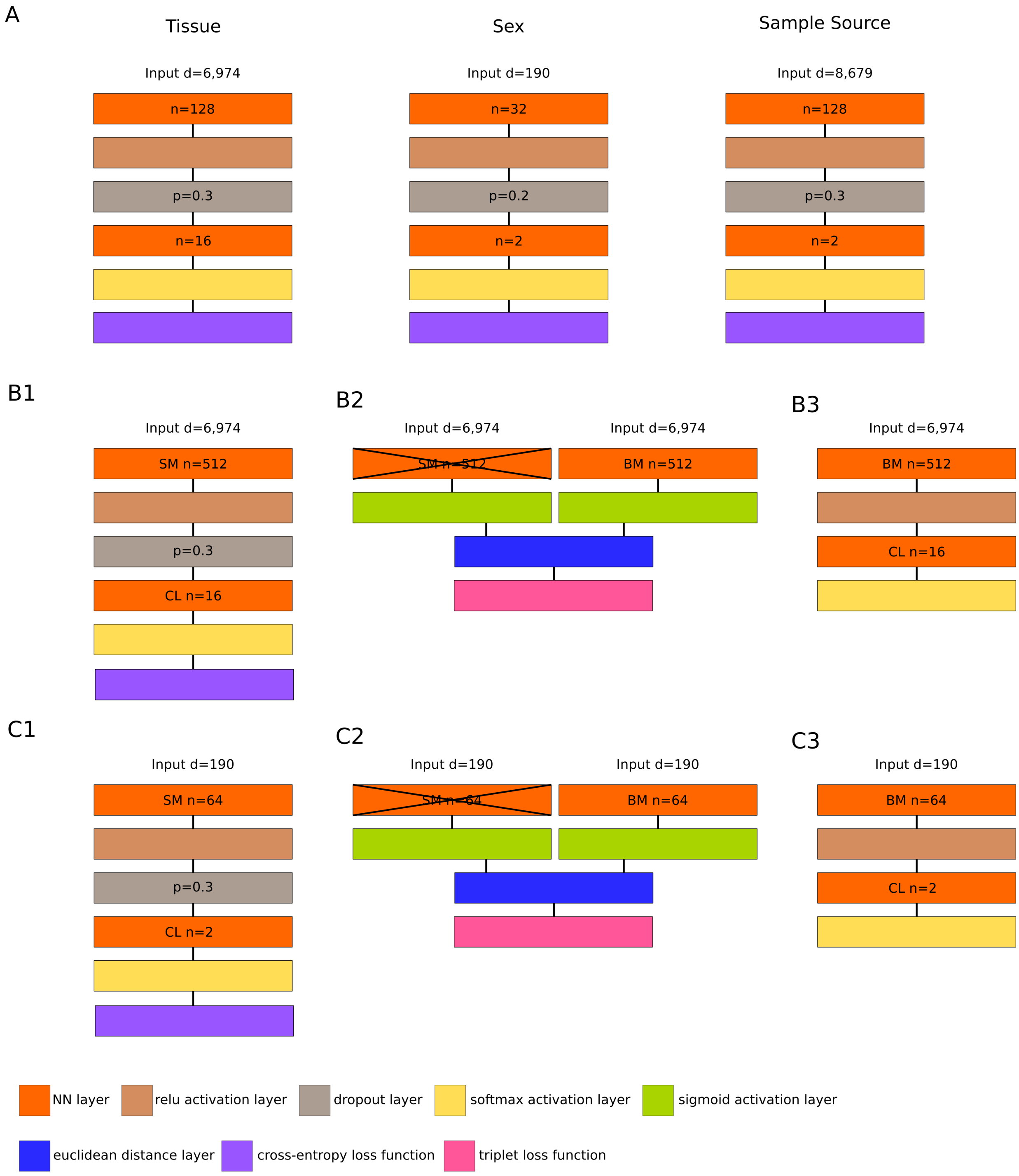

Fig. S5. Graphical Representation of Architectures for ANN Based Models. A) MLP models for tissue, sex and sample source, B) are the (1) SM-CL MLP, (2) SM-BM Siamese Network and (3) BM-CL prediction models for tissue and C) sex. Each rectangle represents a layer in the neural network and is colored according to the type of layer that has been used. d=input dimension, n=number of nodes, p=drop out probability, SM=source mapper, BM=bias mapper, CL=classification layer. B2 and C2 show the SM to have frozen weights.

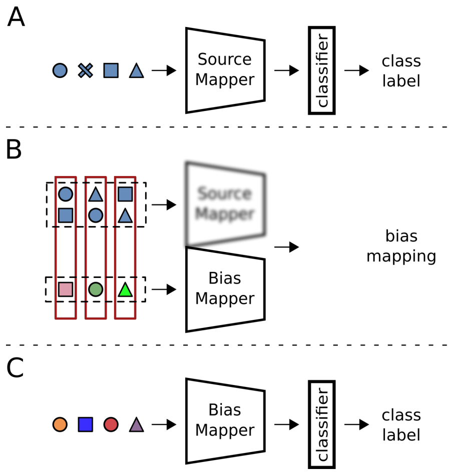

Fig. S6. Overview of DA Model. Samples are indicated according to their classes (circles, squares, triangles) and their bias (blue: source domain, other colors: bias domain, target domain). The model is ready for prediction after two training steps: A) A source mapper is trained on single bias data together with a classification layer. B) A bias mapper is created as a duplicate of the source mapper, the weights of the source mapper are fixed. Triplets are passed through the source mapper and bias mapper configuration to learn a bias mapping. C) The bias mapper, equipped with a classification layer, can be used to predict data from previously unseen datasets.

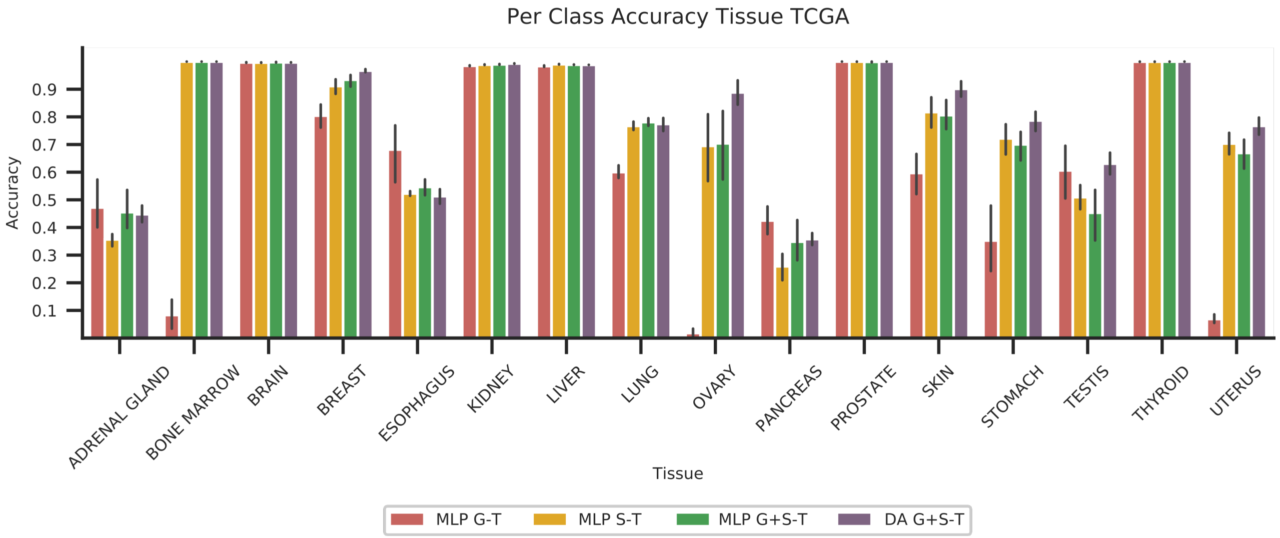

Fig. S7. Per Class Accuracy for TCGA Tissue Classification. Mean sample accuracy for each tissue and all ANN based models is shown. The error bar shows the standard deviation across 10 random seeds. The plot demonstrates the varied tissue classification performance of different tissues. For instance, it seems to be difficult to identify adrenal gland or pancreas with any of the models. In particular, the bad classification performance of MLP G-T for bone marrow, ovary and uterus is especially noticeable, along with the observation that performance can be salvaged by addition of (biased) SRA data to the training data set. This highlights the strength of ANN based models in capturing bias from training data.

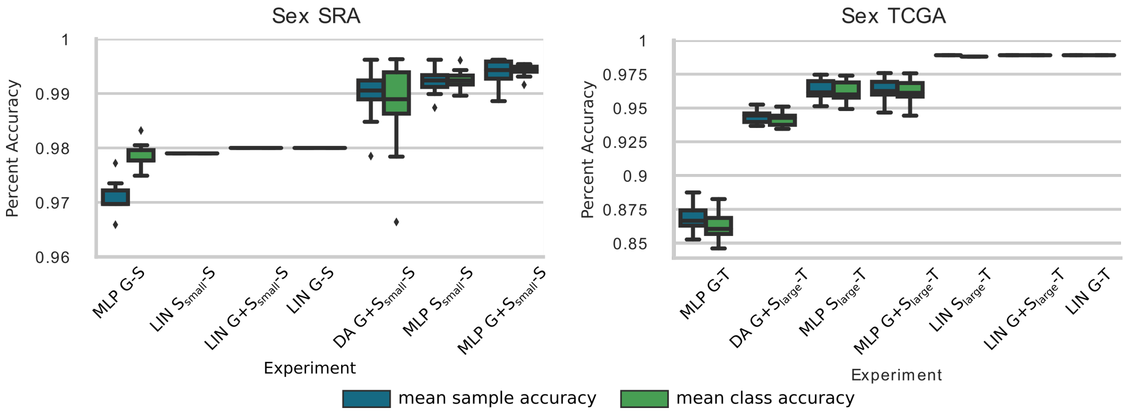

Fig. S8. Sex Phenotype Results. A) SRA and B) TCGA test data. LIN=linear model, MLP=multilayer perceptron, DA=domain adaptation, G=GTEx, S=SRA and T=TCGA. ANN based models yielded consistently worse results than the baseline model, until newly annotated data were incorporated into the training set.

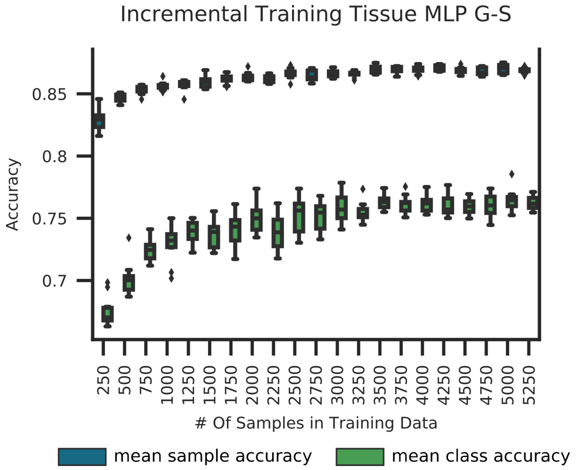

Fig. S9. Dependence of Prediction Performance on Increasing Training Data Set Sizes For MLP G-S. MLP models were trained on subsets of the GTEx data for SRA tissue classification on 10 seeds and averaged. At each step the subset was increased by 250 samples. Box Plots from 20 iterations for the msa and mca are shown in blue and green, respectively. Mean sample accuracy reaches its peak with only 25% of the training data, while 50% of the data is sufficient for the mean class accuracy to saturate.

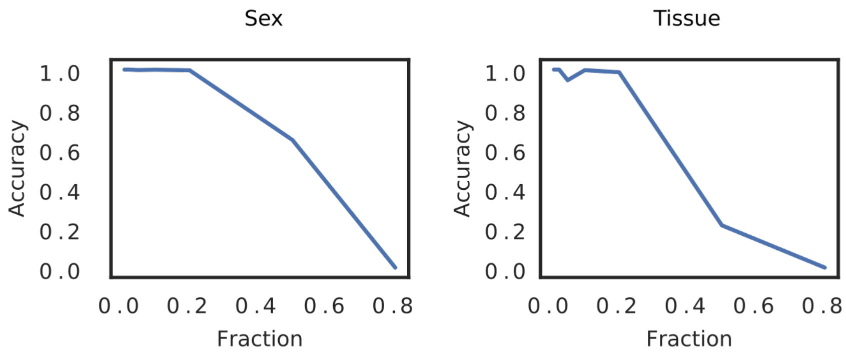

Fig. S10. Test of Overfitting An MLP model was trained on GTEx data. An increasing fraction of one class was assigned a wrong class label (e.g., brain to skin). The model was trained on the partially mislabeled data and the mislabeled data was predicted by the model after training. We quantify the model's susceptibility to overfitting by letting it correct the mislabeled training data. The MLP model was able to correct all mislabeled data up to a mislabeling fraction of 20%. We conclude that the ANN models are very robust in dealing with mislabeled data.

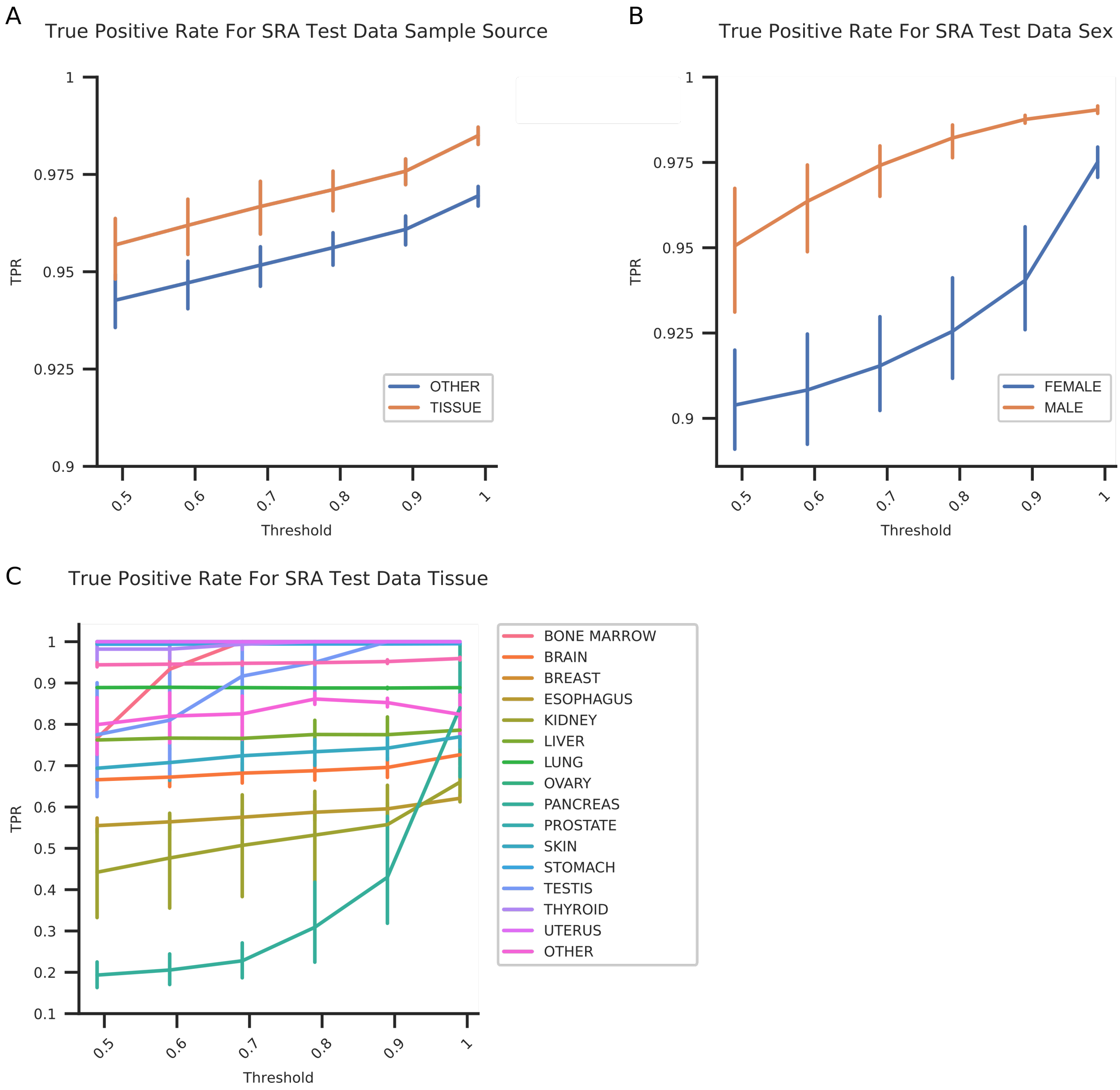

Fig. S11. True Positive Rate for Test Data Predicted With Annotation Models. (A) Sample source, (B) sex and (C) tissue classification.

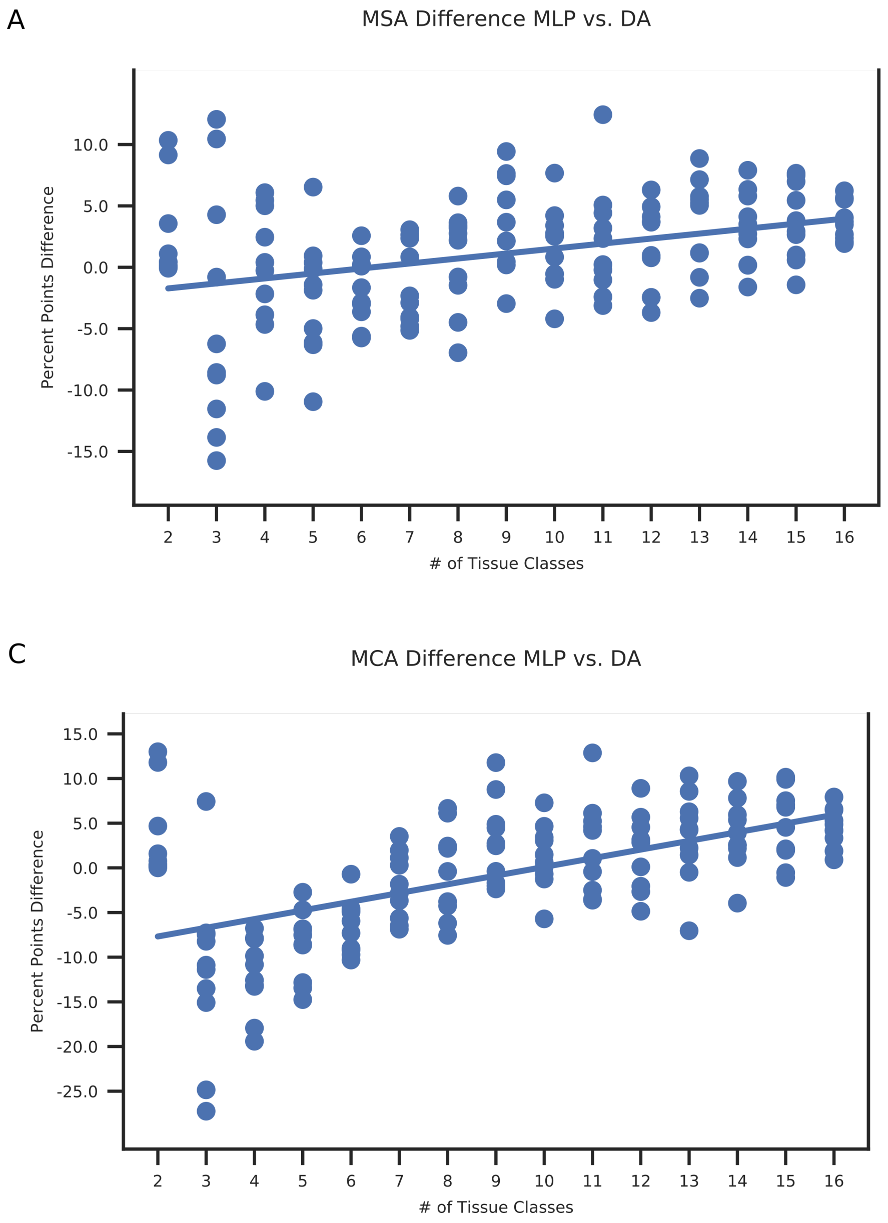

Fig. S12. Relationship Between Number of Classes And DA Performance in DA G+S-T. The 16 tissues were sorted by sample size in GTEx, at each step one tissue was added to the classification problem, starting with the largest two. MLP and DA were trained as described above for 10 seeds each and tested on TCGA data. The mean sample accuracy for each seed (top panel) or mean class accuracy (bottom panel) are shown. Each dot shows the difference in accuracy (DA-MLP) at each step for each seed. Seaborn's regplot was used to fit a regression line. While, on average, MLP performs better for lower number of classes, the performance gain by the DA model with respect to MLP increases with the number of classes.

Table S1. Mapping from GTEx tissue names to MetaSRA tissue names.

GTEx	MetaSRA
ovary	female gonad
skin	anatomical skin
thyroid	thyroid gland
prostate	prostate gland
bladder	urinary bladder
cervix uteri	uterine cervix

Table S2. Summary of the datasets used for each phenotype after pre-processing.

Dataset	# Samples	# Classes	# Input genes	Gini cut off Low	Gini cut off High
**Tissue**					
GTEx	5,480	16	6,974	0.5	1
TCGA	8,624				
SRA train	1,721				
SRA test	1,531				
**Sex**					
GTEx	9,662	2	190	0.4	0.7
TCGA	11,284				
SRA train	2,017				
SRA test	791				
**Sample Source**					
GTEx	9,662	1	8,679	0.3	0.8
TCGA	11,284				
SRA train	12,725				
SRA train	3,144	2			
SRA val	1,124				
SRA train annotation	16,463				
SRA test annotation	3,707				

Table S3. Number of samples per class for phenotype classification experiments.

	GTEx	TCGA	SRA train	SRA test
		Tissue		
Adrenal gland	159	266	14	5
Bone marrow	102	126	77	90
Brain	1,409	707	508	770
Breast	218	1246	123	30
Esophagus	790	198	35	5
Kidney	36	1030	94	88
Liver	136	424	111	134
Lung	374	1156	228	72
Ovary	108	430	23	12
Pancreas	197	183	17	5
Prostate	119	558	123	49
Skin	974	473	238	198
Stomach	204	453	25	11
Testis	203	156	14	18
Thyroid	361	572	51	32
Uterus	90	646	40	12
		Sex		
Male	6,036	5,395	1,217	538
Female	3,326	5,889	800	253
		Sample source		
Cell line	9,662	11,284	7,108	1,950
Biopsy	-	-	5,617	1,194

Table S4. Hyperparameters considered during model tuning and their initial range.

Hyperparameter	Range	Sampling mode
# Layers	[0,3]	linear
# Nodes per layer	[32,512]	linear
Batch size	[16,32,64]	step
Learning rate	[1e-4, 1e-2]	log
Optimizer	[Adam, SGD]	binary
Drop out	[0.1,0.2,0.3]	step
Gini cut off	manually	manually

Table S5. Summary of the hyperparameters used for each model.

Model	# Nodes	Dropout rate	Learning rate	Margin
MLP Tissue	128	0.3	0.0002	-
MLP Sex	32	0.2	0.0024	-
MLP Sample Source	128	0.3	0.0002	-
DA SM-CL Tissue	512 / 16	0.3	0.0001	-
DA SM-BM Tissue	512 / 512	-	0.0005	5
DA SM-CL Sex	64 / 2	0.3	0.0001	-
DA SM-BM Sex	64 / 64	-	0.0005	3

For every model 1 hidden layer was used, batch size was 64, trained epochs were 10 and the optimizer used Adam.

Table S6. Sample and class accuracy given are the mean over n=10 seeds

	msa	mca	msa std.	mca std.
	Tissue			
	SRA			
LIN G-S	0.893	0.765	NA	NA
LIN S_{small}-S	0.893	0.795	NA	NA
LIN G+S_{small}-S	0.908	0.785	NA	NA
MLP G-S	0.872	0.77	0.007	0.018
MLP S_{small}-S	0.894	0.746	0.005	0.017
MLP G+S_{small}-S	0.915	0.817	0.008	0.02
DA G+S_{small}-S	0.922	0.821	0.003	0.009
	TCGA			
LIN G-T	0.718	0.638	NA	NA
LIN S_{large}-T	0.784	0.724	NA	NA
LIN G+S_{large}-T	0.725	0.651	NA	NA
MLP G-T	0.684	0.605	0.015	0.017
MLP S_{large}-T	0.832	0.755	0.02	0.03
MLP G+S_{large}-T	0.842	0.773	0.015	0.017
DA G+S_{large}-T	0.875	0.813	0.004	0.006
LIN S_{small}-T	0.768	0.708	NA	NA
LIN G+S_{small}-T	0.729	0.658	NA	NA
MLP S_{small}-T	0.748	0.688	0.016	0.027
MLP G+S_{small}-T	0.764	0.716	0.033	0.028
DA G+S_{small}-T	0.81	0.763	0.014	0.024
	Sex			
	SRA			
LIN G-S	0.98	0.98	NA	NA
LIN S_{small}-S	0.979	0.979	NA	NA
LIN G+S_{small}-S	0.98	0.98	NA	NA
MLP G-S	0.971	0.979	0.002	0.04
MLP S_{small}-S	0.994	0.994	0.008	0.009
MLP G+S_{small}-S	0.993	0.992	0.003	0.003
DA G+S_{small}-S	0.99	0.987	0.025	0.036
	TCGA			
LIN G-T	0.989	0.989	NA	NA
LIN S_{large}-T	0.988	0.987	NA	NA
LIN G+S_{large}-T	0.989	0.989	NA	NA
MLP G-T	0.869	0.863	0.011	0.011
MLP S_{large}-T	0.963	0.962	0.01	0.01
MLP G+S_{large}-T	0.964	0.962	0.011	0.011
DA G+S_{large}-T	0.944	0.942	0.004	0.004
	Sample source			
LIN S_{large}-G	0.951	0.951	NA	NA
LIN S_{large}-T	0.882	0.882	NA	NA
LIN S_{small}-S	0.89	0.884	NA	NA
MLP S_{large}-G	0.943	0.943	0.001	0.001
MLP S_{large}-T	0.971	0.971	0.028	0.028
MLP S_{small}-S	0.95	0.941	0.003	0.005

msa=mean sample accuracy, mca=mean class accuracy, G=GTEx, T=TCGA, S=SRA, NA=not available

Click here to access/download;Figure;figure_S4_sx_sumy.png

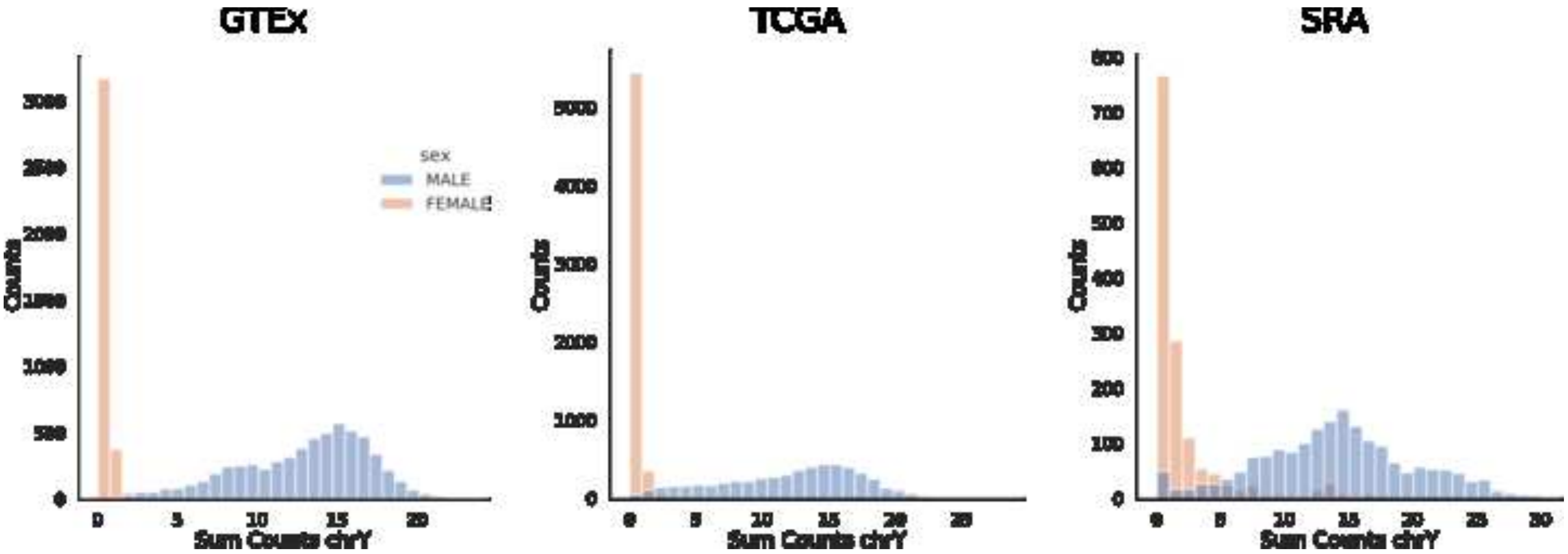

Click here to
access/download;Figure;figure_S5_model_config.png

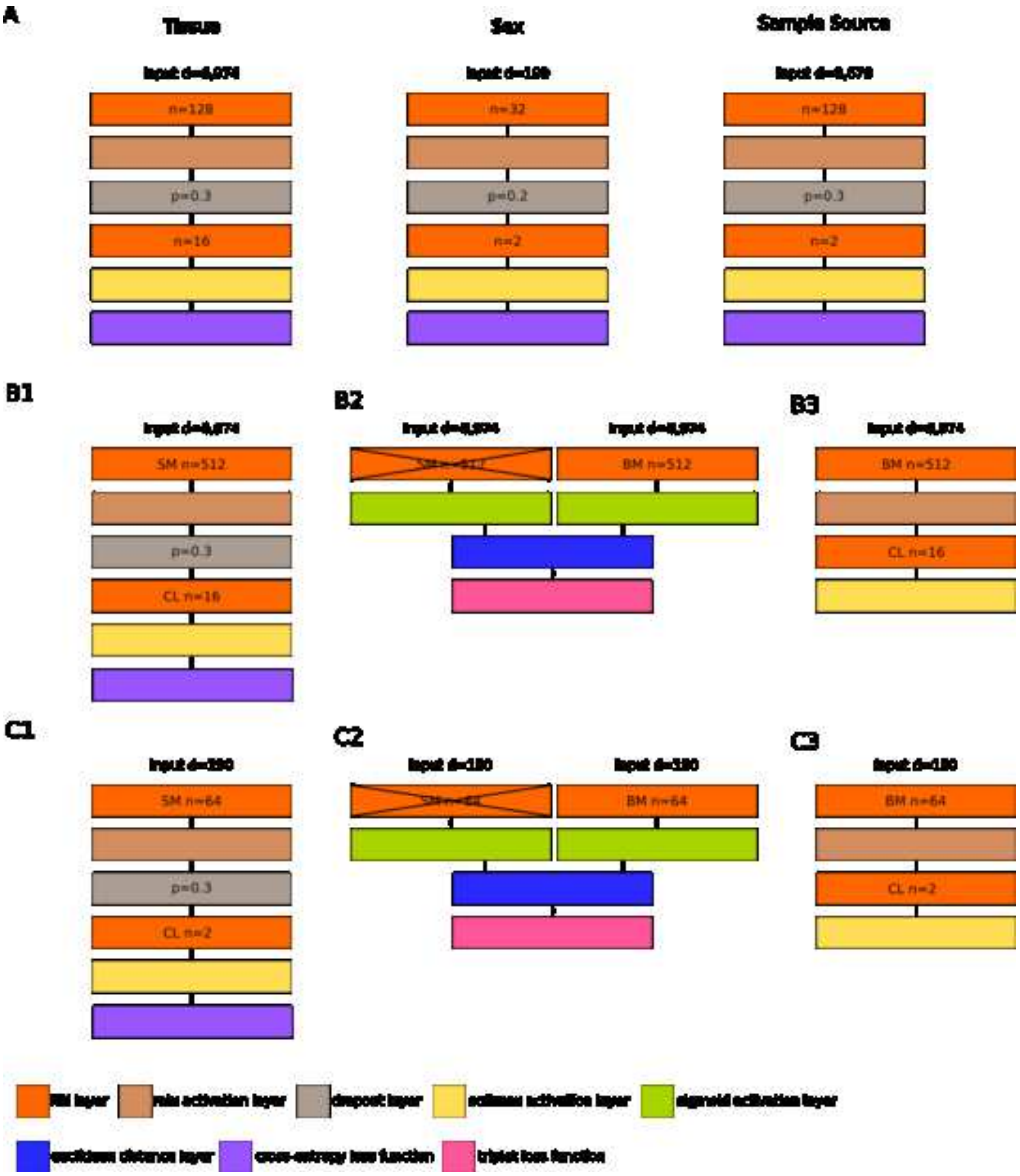

Click here to access/download;Figure;figure_S6_da_short.png

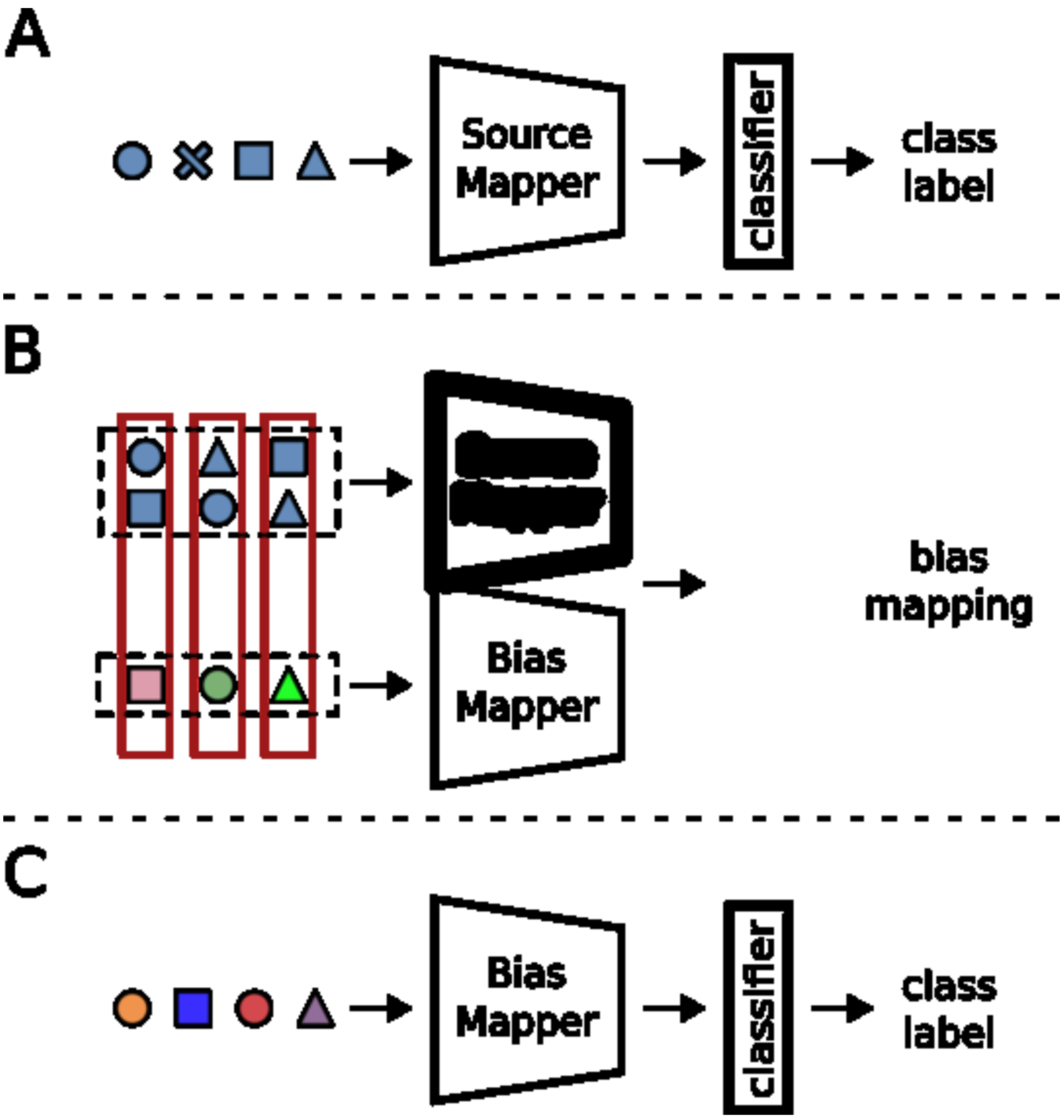

Click here to access/download;Figure;figure_S7_per_class_accuracy.png

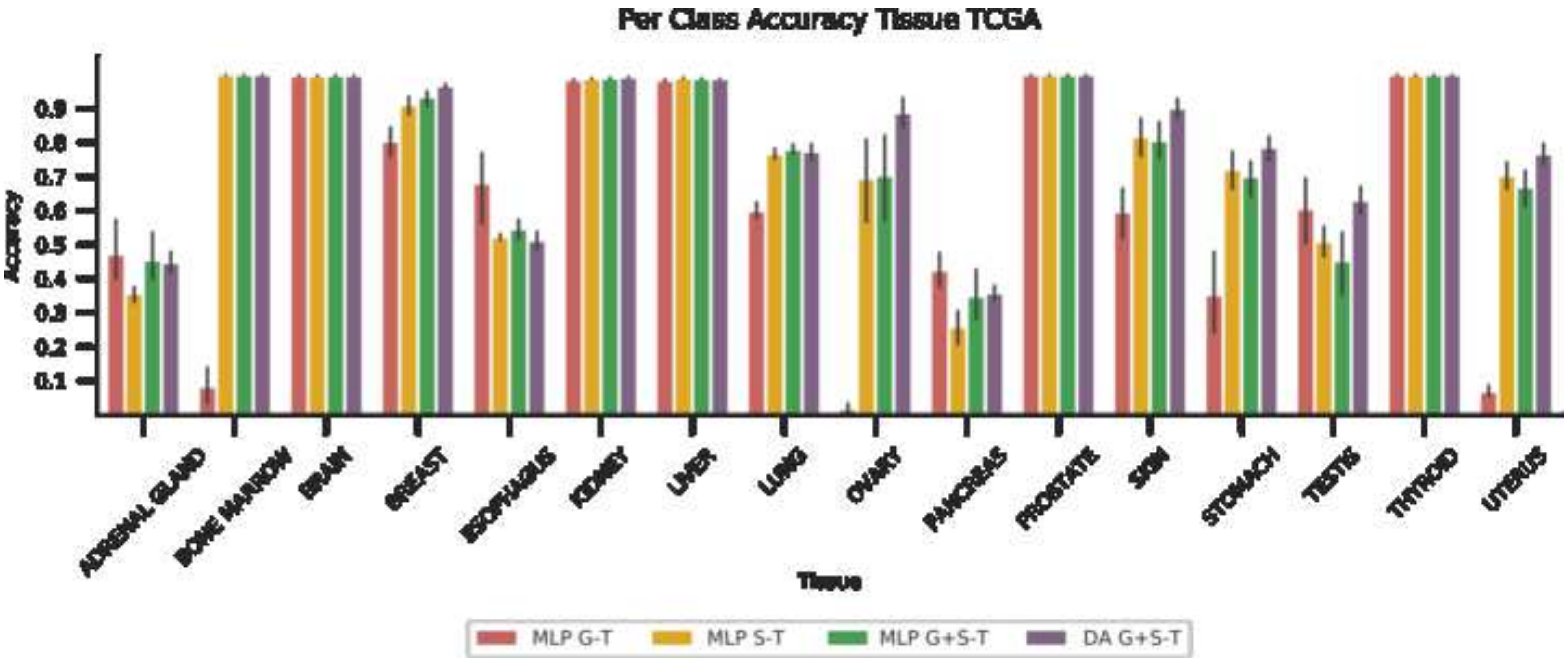

Click here to access/download;Figure;figure_S8_sex_tcga.png

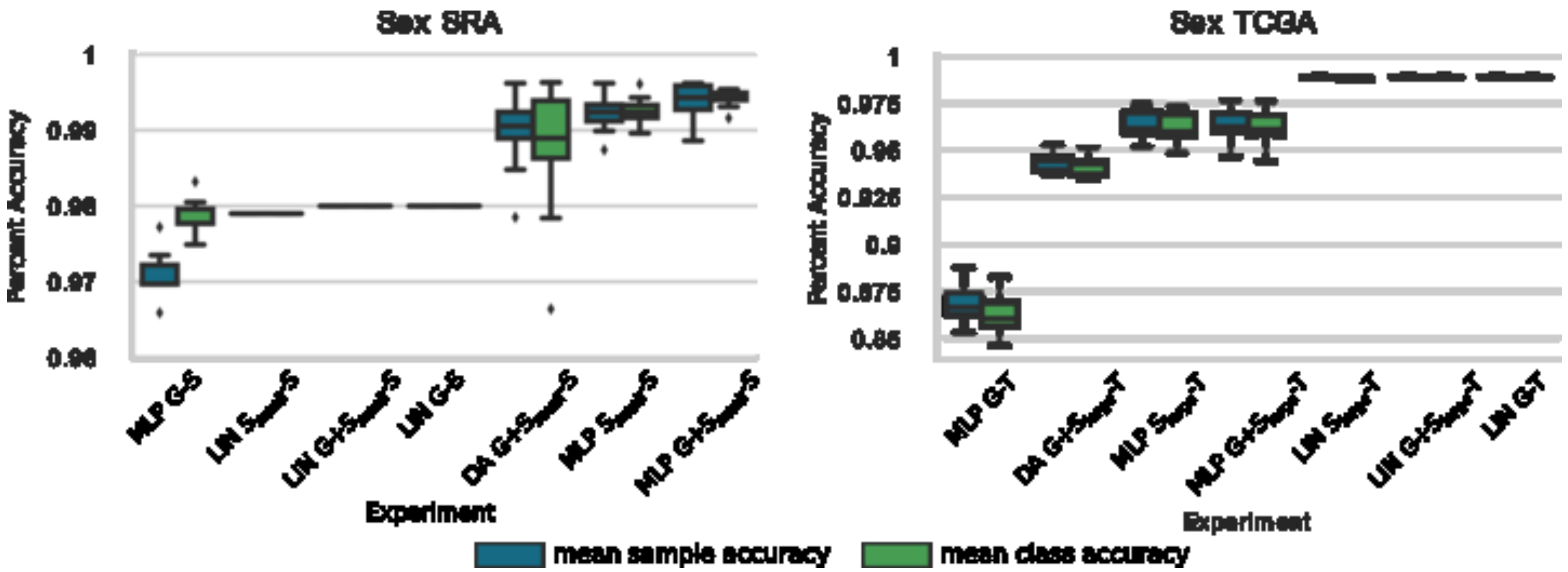

Click here to access/download;Figure;figure_S10_overfit_training_data.png

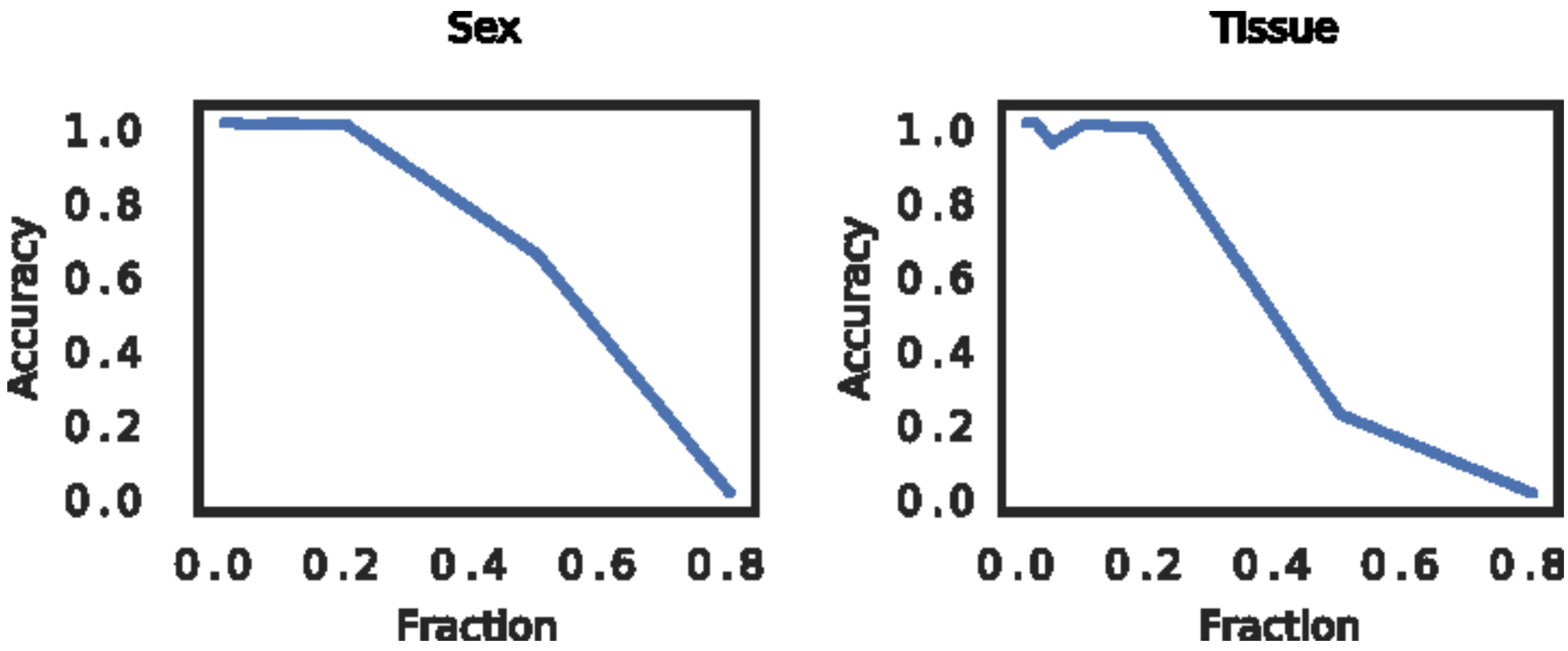

Click here to access/download;Figure;figure_S11_tpr.png

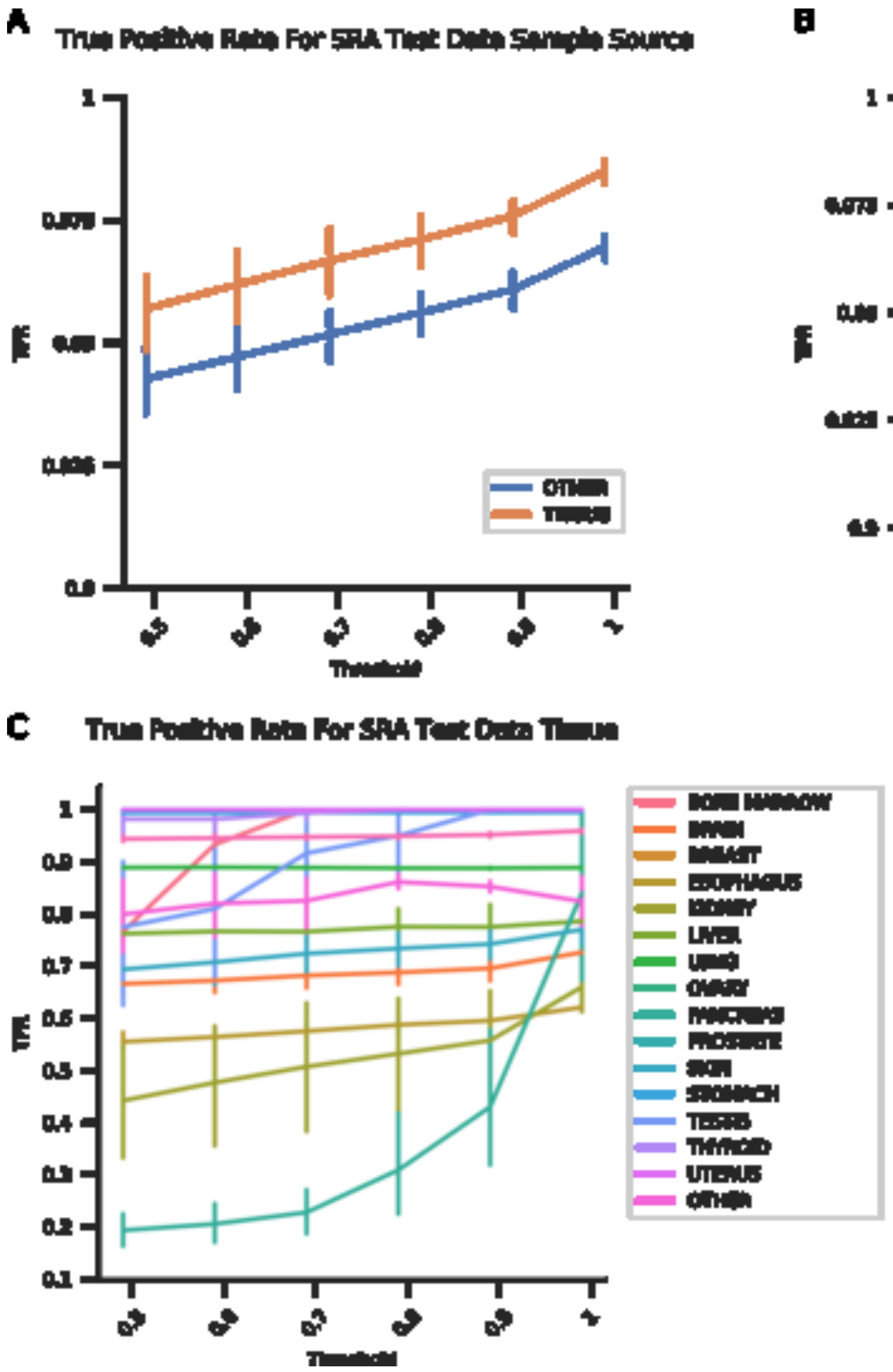

Click here to access/download;Figure;figure_S12_mlp_vs_da.png

A

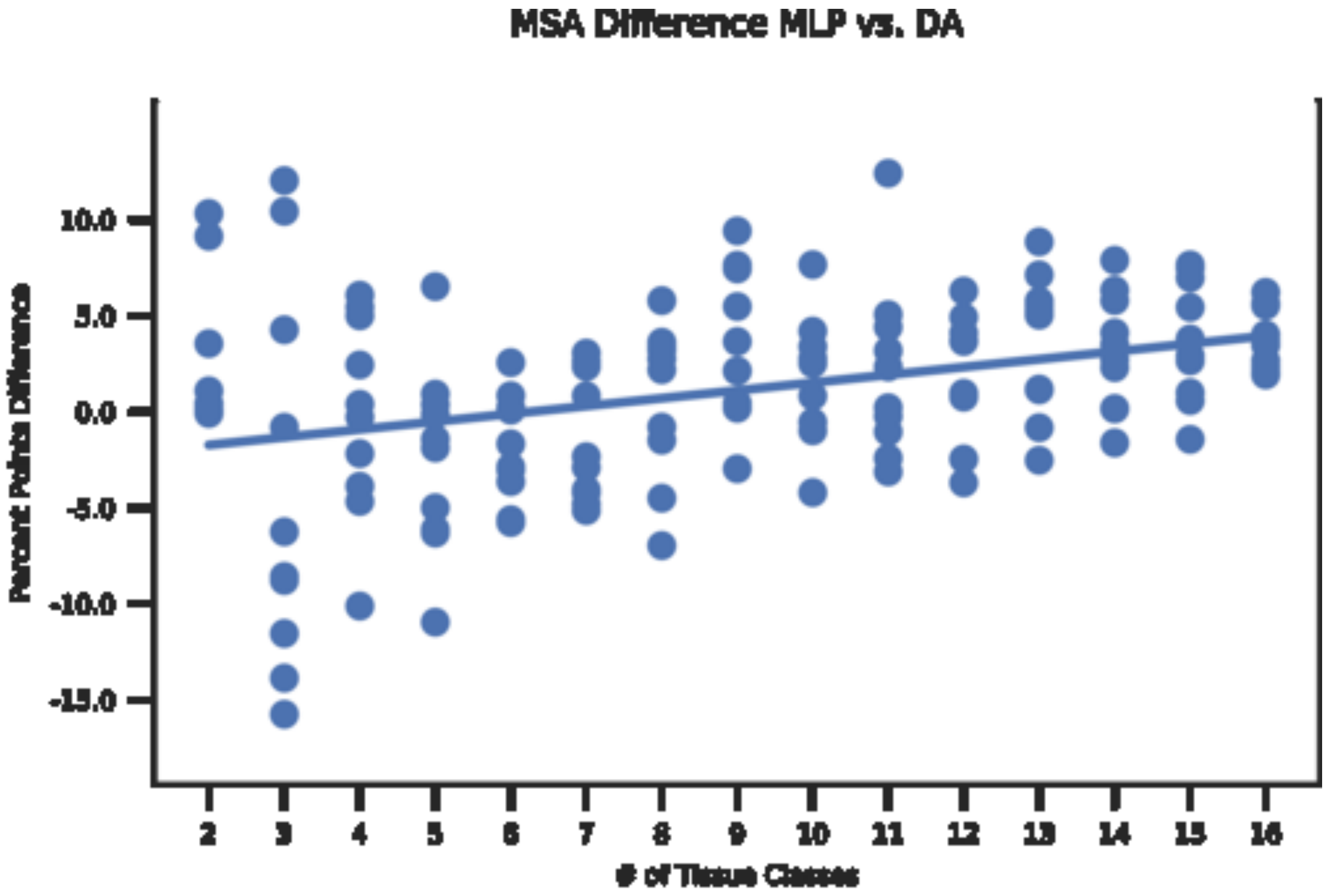

C

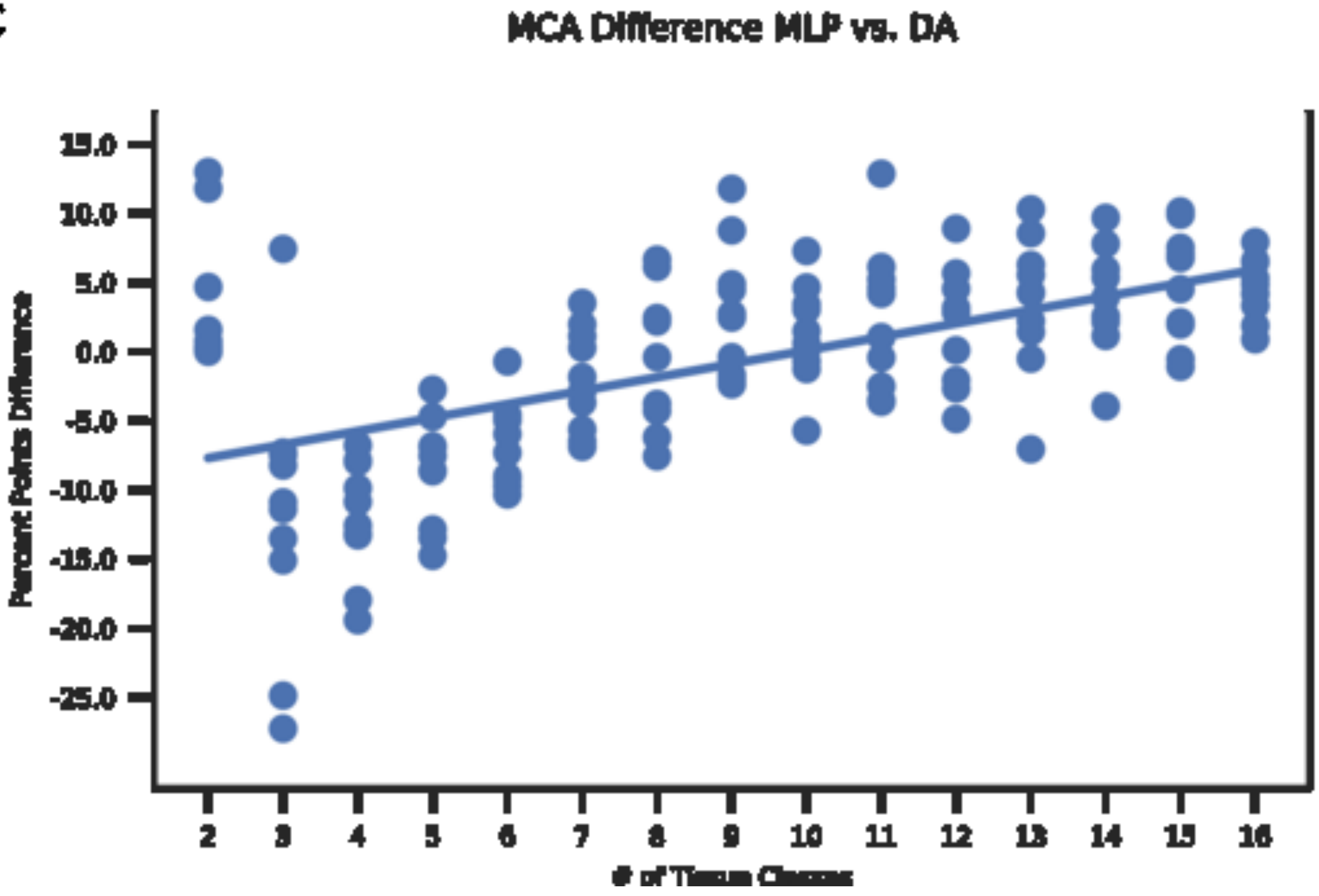

Click here to access/download;Figure;figure_1.png

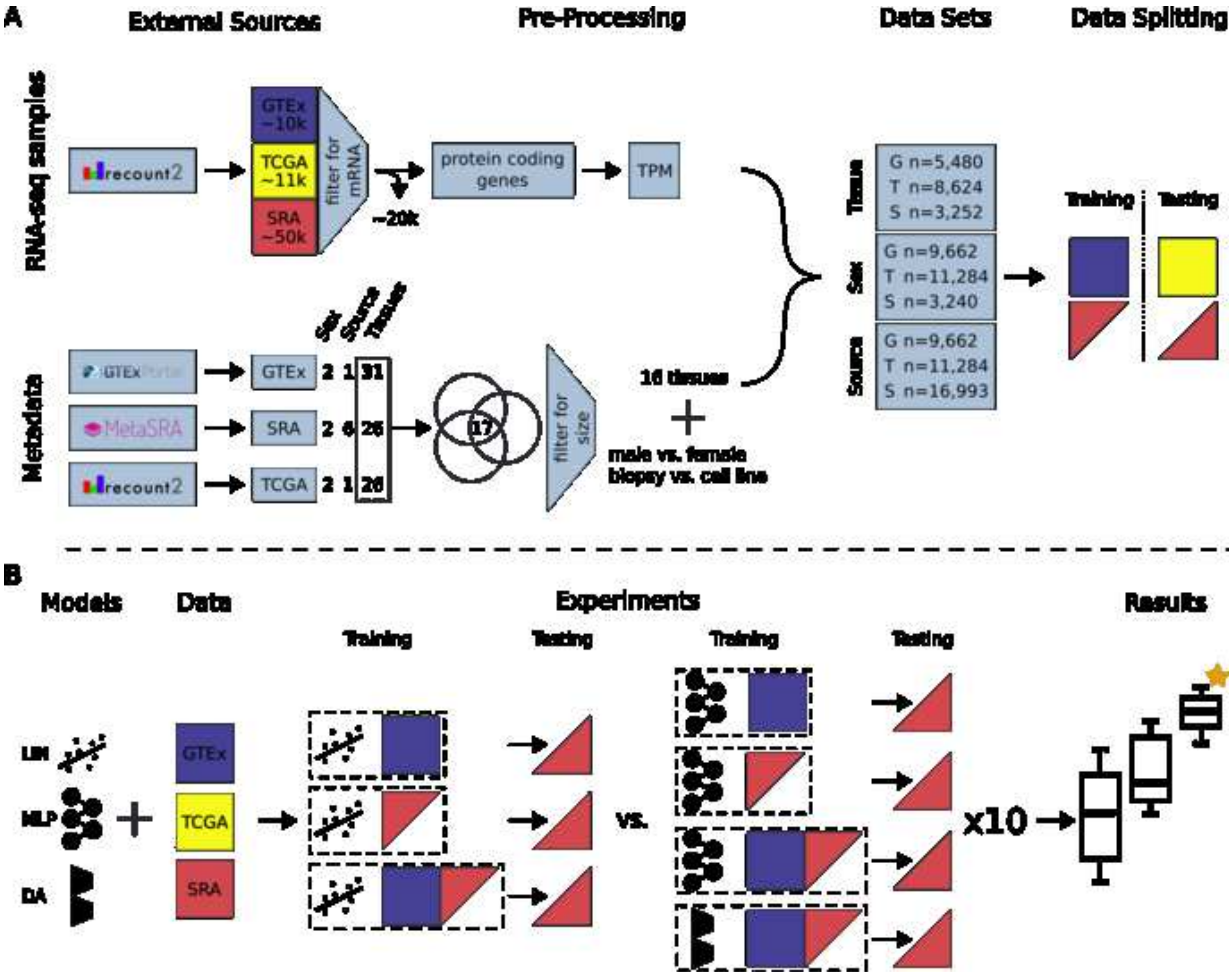

Click here to access/download;Figure;figure_3.png

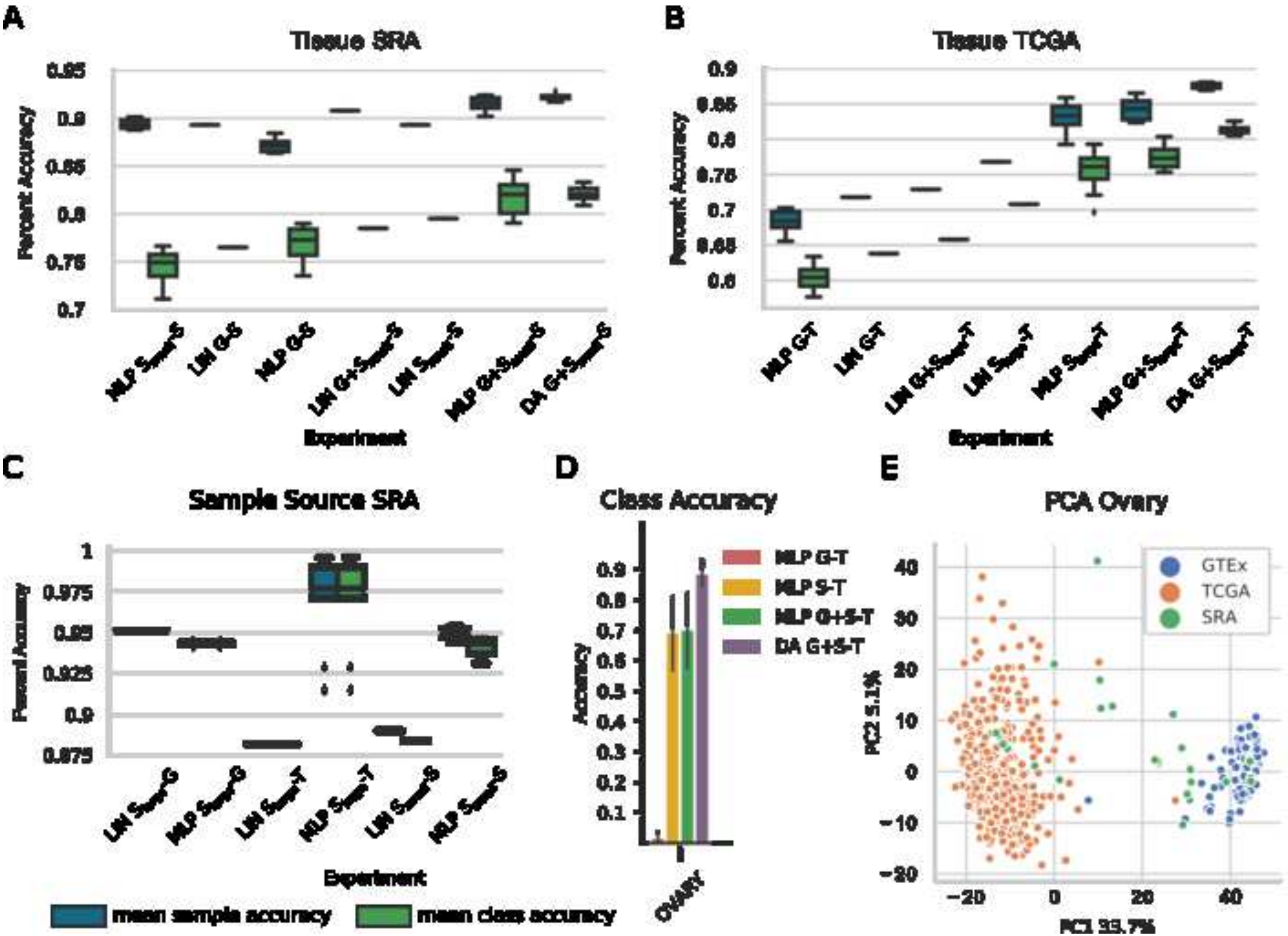

Click here to access/download;Figure;figure_S1_bias_vis.png

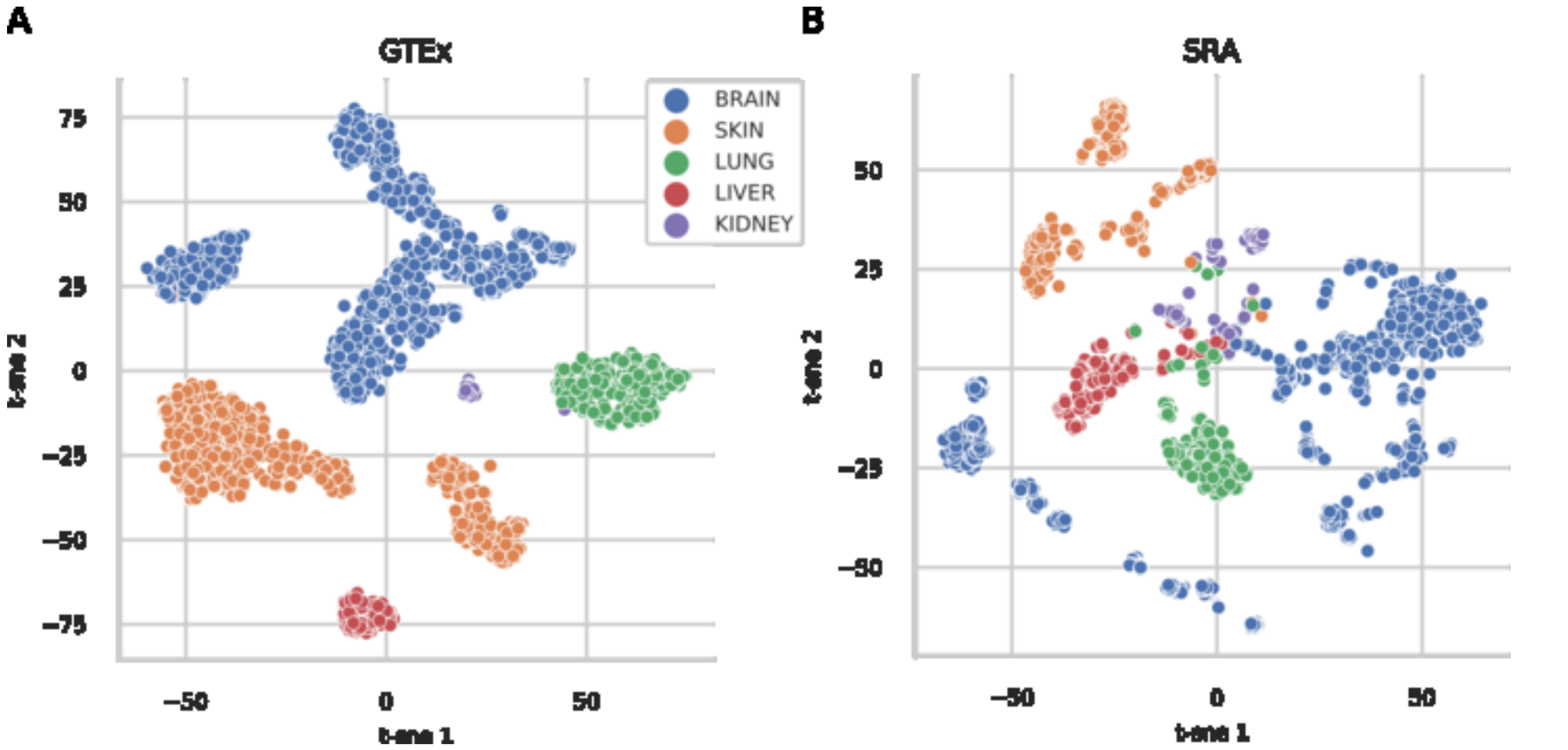

Manuscript

Click here to access/download
Supplementary Material
LetterToTheEditor.pdf

Manuscript

Click here to access/download
Supplementary Material
answers to reviewers.pdf

Click here to access/download
Supplementary Material
new_annotations_tissue.csv

Click here to access/download
Supplementary Material
new_annotations_sex.csv

Click here to access/download
Supplementary Material
new_annotations_sample_source.csv

Click here to access/download
Supplementary Material
metasra_mismatch_tissue.csv

Click here to access/download
Supplementary Material
metasra_mismatch_sex.csv

Click here to access/download
Supplementary Material
metasra_mismatch_sample_source.csv

Click here to access/download
Supplementary Material
labeled_sample_ids_TCGA_tissue.csv

Click here to access/download
Supplementary Material
labeled_sample_ids_TCGA_sex.csv

Click here to access/download
Supplementary Material
labeled_sample_ids_TCGA_sample_source.csv

Click here to access/download
Supplementary Material
labeled_sample_ids_SRA_val_sample_source.csv

Click here to access/download
Supplementary Material
labeled_sample_ids_SRA_train_tissue.csv

Click here to access/download

Supplementary Material

labeled_sample_ids_SRA_train_sex.csv

Click here to access/download
Supplementary Material
labeled_sample_ids_SRA_train_sample_source.csv

Click here to access/download
Supplementary Material
labeled_sample_ids_SRA_test_tissue.csv

Click here to access/download
Supplementary Material
labeled_sample_ids_SRA_test_sex.csv

Click here to access/download
Supplementary Material
labeled_sample_ids_SRA_test_sample_source.csv

Click here to access/download
Supplementary Material
labeled_sample_ids_GTEx_tissue.csv

Click here to access/download
Supplementary Material
labeled_sample_ids_GTEx_sex.csv

Click here to access/download
Supplementary Material
labeled_sample_ids_GTEx_sample_source.csv

Click here to access/download

Supplementary Material

input_features.xlsx
