# Supplementary material for: Bias-invariant RNA-sequencing metadata annotation
Source: Gigascience. 2021 Sep 22;10(9):giab064. doi: 10.1093/gigascience/giab064 (PMC8559615; doi:10.1093/gigascience/giab064)
Supplement: giab064_Supplemental_Files [file giab064_supplemental_files.zip › tables.pdf]

Table S1: Mapping from GTEx tissue names to MetaSRA tissue names.

GTEx	MetaSRA
ovary	female gonad
skin	anatomical skin
thyroid	thyroid gland
prostate	prostate gland
bladder	urinary bladder
cervix uteri	uterine cervix

Table S2: Summary of the datasets used for each phenotype after pre-processing.

Dataset	# Samples	# Classes	# Input genes	Gini cut off Low	Gini cut off High
		Tissue			
GTEx	5,480	16	6,974	0.5	1
TCGA	8,624				
SRA train	1,721				
SRA test	1,531				
		Sex			
GTEx	9,662	2	190	0.4	0.7
TCGA	11,284				
SRA train	2,017				
SRA test	791				
		Sample Source			
GTEx	9,662	1	8,679	0.3	0.8
TCGA	11,284				
SRA train	12,725				
SRA train	3,144	2			
SRA val	1,124				
SRA train annotation	16,463				
SRA test annotation	3,707				

Table S3: Number of samples per class for phenotype classification experiments.

	GTEx	TCGA	SRA train	SRA test
		Tissue		
Adrenal gland	159	266	14	5
Bone marrow	102	126	77	90
Brain	1,409	707	508	770
Breast	218	1246	123	30
Esophagus	790	198	35	5
Kidney	36	1030	94	88
Liver	136	424	111	134
Lung	374	1156	228	72
Ovary	108	430	23	12
Pancreas	197	183	17	5
Prostate	119	558	123	49
Skin	974	473	238	198
Stomach	204	453	25	11
Testis	203	156	14	18
Thyroid	361	572	51	32
Uterus	90	646	40	12
		Sex		
Male	6,036	5,395	1,217	538
Female	3,326	5,889	800	253
		Sample source		
Cell line	9,662	11,284	7,108	1,950
Biopsy	-	-	5,617	1,194

Table S4: Hyperparameters considered during model tuning and their initial range.

Hyperparameter	Range	Sampling mode
# Layers	[0,3]	linear
# Nodes per layer	[32,512]	linear
Batch size	[16,32,64]	step
Learning rate	[1e-4, 1e-2]	log
Optimizer	[Adam, SGD]	binary
Drop out	[0.1,0.2,0.3]	step
Gini cut off	manually	manually

Table S5: Summary of the hyperparameters used for each model.

Model	# Nodes	Dropout rate	Learning rate	Margin
MLP Tissue	128	0.3	0.0002	-
MLP Sex	32	0.2	0.0024	-
MLP Sample Source	128	0.3	0.0002	-
DA SM-CL Tissue	512 / 16	0.3	0.0001	-
DA SM-BM Tissue	512 / 512	-	0.0005	5
DA SM-CL Sex	64 / 2	0.3	0.0001	-
DA SM-BM Sex	64 / 64	-	0.0005	3

For every model 1 hidden layer was used, batch size was 64, trained epochs were 10 and the optimizer used Adam.

Table S6: Sample and class accuracy given are the mean over n=10 seeds

	msa	mca	msa std.	mca std.
**Tissue**				
**SRA**				
LIN G-S	0.893	0.765	NA	NA
LIN S_{small}-S	0.893	0.795	NA	NA
LIN G+S_{small}-S	0.908	0.785	NA	NA
MLP G-S	0.872	0.77	0.007	0.018
MLP S_{small}-S	0.894	0.746	0.005	0.017
MLP G+S_{small}-S	0.915	0.817	0.008	0.02
DA G+S_{small}-S	0.922	0.821	0.003	0.009
**TCGA**				
LIN G-T	0.718	0.638	NA	NA
LIN S_{large}-T	0.784	0.724	NA	NA
LIN G+S_{large}-T	0.725	0.651	NA	NA
MLP G-T	0.684	0.605	0.015	0.017
MLP S_{large}-T	0.832	0.755	0.02	0.03
MLP G+S_{large}-T	0.842	0.773	0.015	0.017
DA G+S_{large}-T	0.875	0.813	0.004	0.006
LIN S_{small}-T	0.768	0.708	NA	NA
LIN G+S_{small}-T	0.729	0.658	NA	NA
MLP S_{small}-T	0.748	0.688	0.016	0.027
MLP G+S_{small}-T	0.764	0.716	0.033	0.028
DA G+S_{small}-T	0.81	0.763	0.014	0.024
**Sex**				
**SRA**				
LIN G-S	0.98	0.98	NA	NA
LIN S_{small}-S	0.979	0.979	NA	NA
LIN G+S_{small}-S	0.98	0.98	NA	NA
MLP G-S	0.971	0.979	0.002	0.04
MLP S_{small}-S	0.994	0.994	0.008	0.009
MLP G+S_{small}-S	0.993	0.992	0.003	0.003
DA G+S_{small}-S	0.99	0.987	0.025	0.036
**TCGA**				
LIN G-T	0.989	0.989	NA	NA
LIN S_{large}-T	0.988	0.987	NA	NA
LIN G+S_{large}-T	0.989	0.989	NA	NA
MLP G-T	0.869	0.863	0.011	0.011
MLP S_{large}-T	0.963	0.962	0.01	0.01
MLP G+S_{large}-T	0.964	0.962	0.011	0.011
DA G+S_{large}-T	0.944	0.942	0.004	0.004
**Sample source**				
LIN S_{large}-G	0.951	0.951	NA	NA
LIN S_{large}-T	0.882	0.882	NA	NA
LIN S_{small}-S	0.89	0.884	NA	NA
MLP S_{large}-G	0.943	0.943	0.001	0.001
MLP S_{large}-T	0.971	0.971	0.028	0.028
MLP S_{small}-S	0.95	0.941	0.003	0.005

msa=mean sample accuracy, mca=mean class accuracy, G=GTEx, T=TCGA, S=SRA, NA=not available
