# Supplementary figures and images for: Bias-invariant RNA-sequencing metadata annotation
Source: Gigascience. 2021 Sep 22;10(9):giab064. doi: 10.1093/gigascience/giab064 (PMC8559615; doi:10.1093/gigascience/giab064)

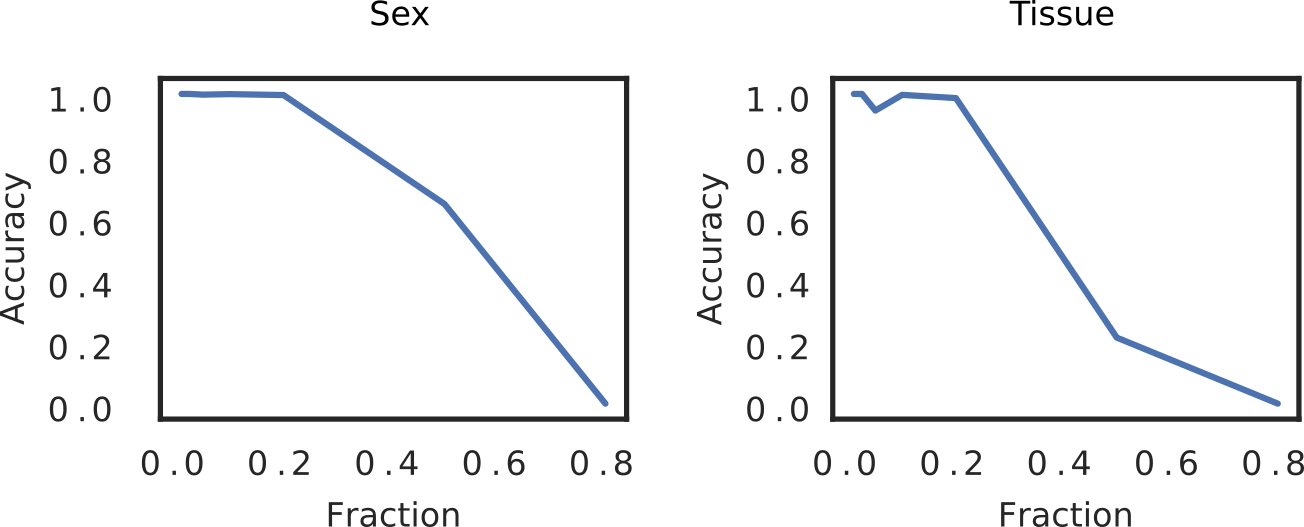

Supplement: giab064_Supplemental_Files [file giab064_supplemental_files.zip › figure_S10_overfit_training_data.png]

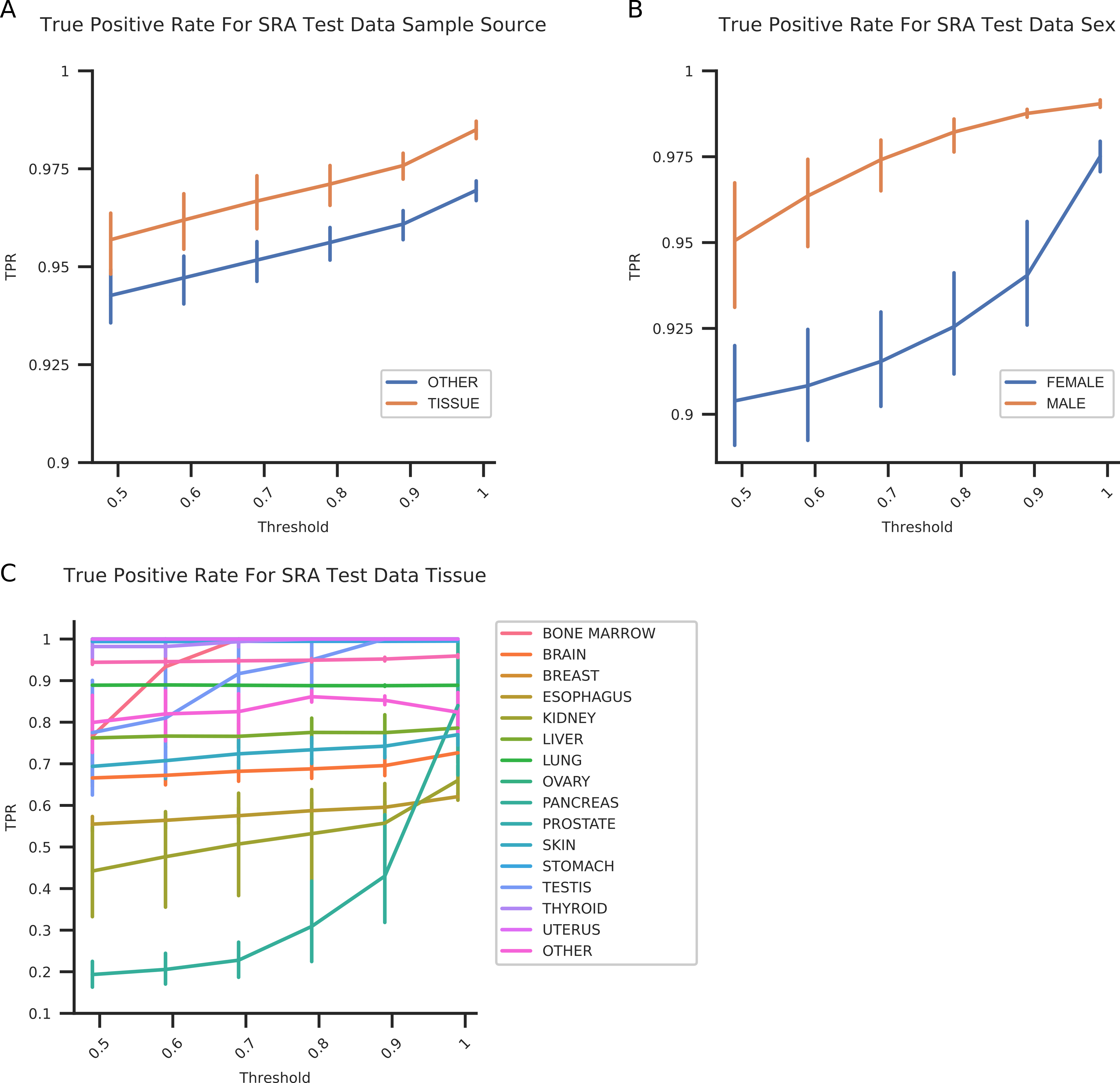

Supplement: giab064_Supplemental_Files [file giab064_supplemental_files.zip › figure_S11_tpr.png]

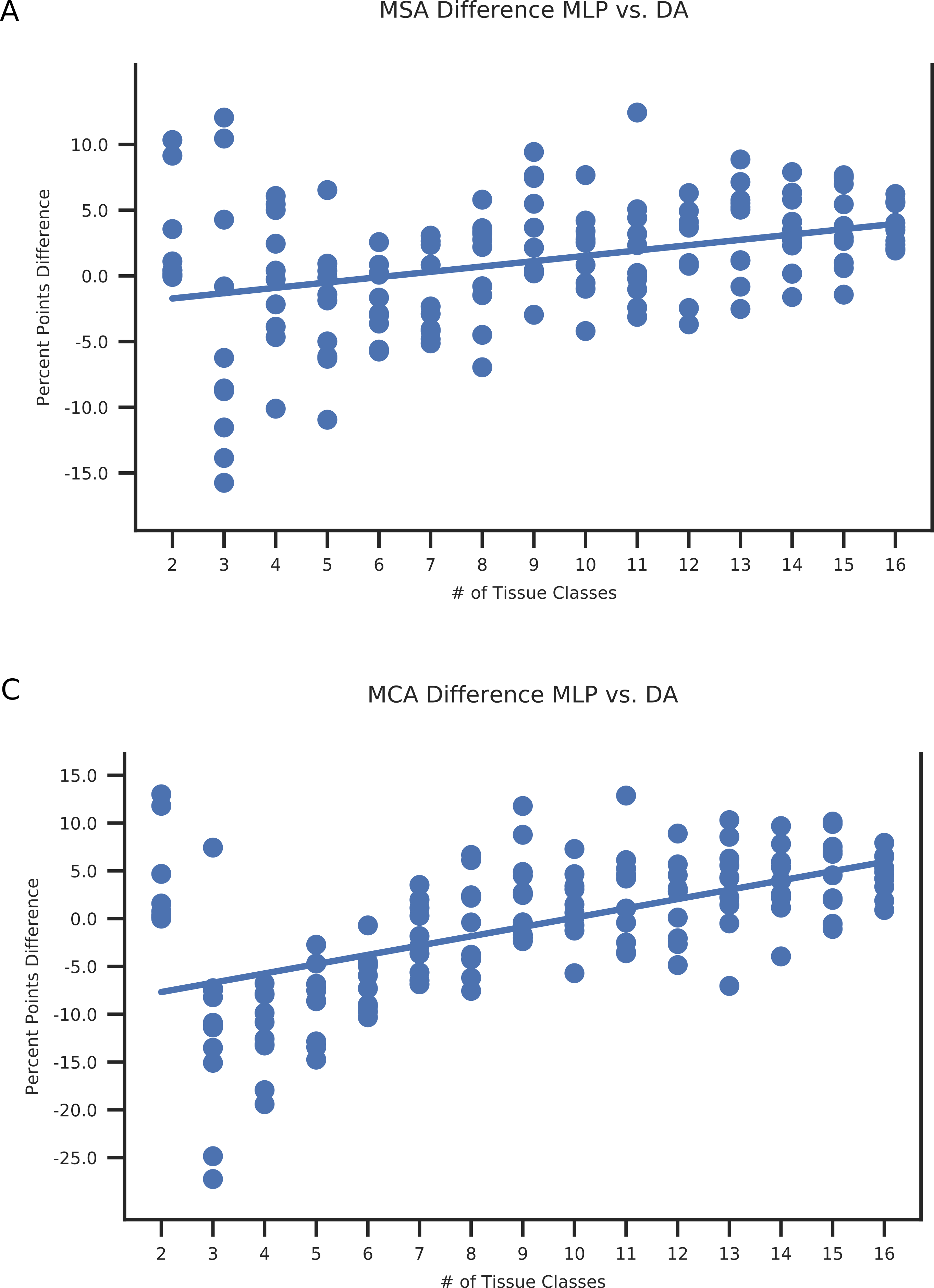

Supplement: giab064_Supplemental_Files [file giab064_supplemental_files.zip › figure_S12_mlp_vs_da.png]

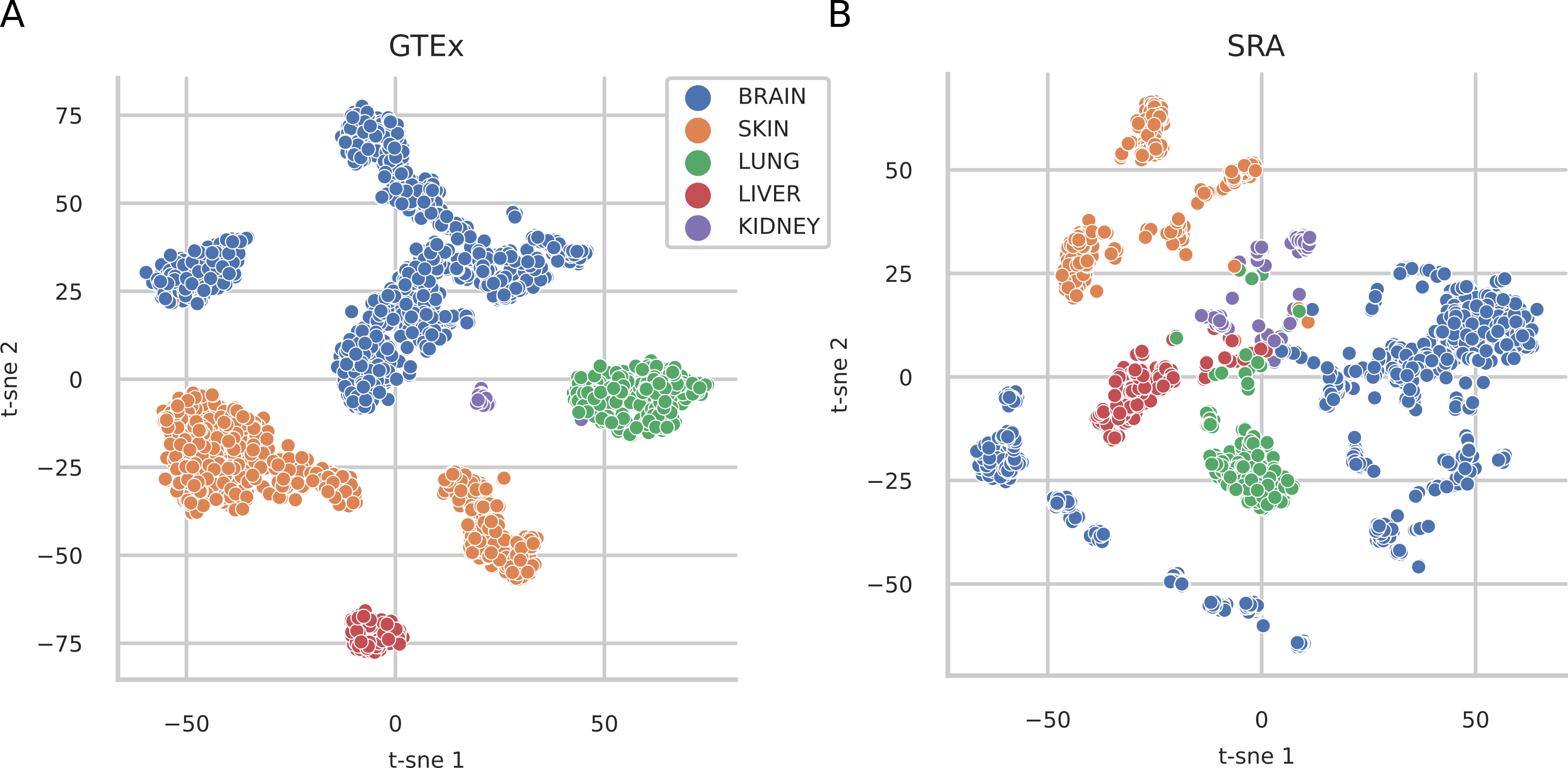

Supplement: giab064_Supplemental_Files [file giab064_supplemental_files.zip › figure_S1_bias_vis.png]

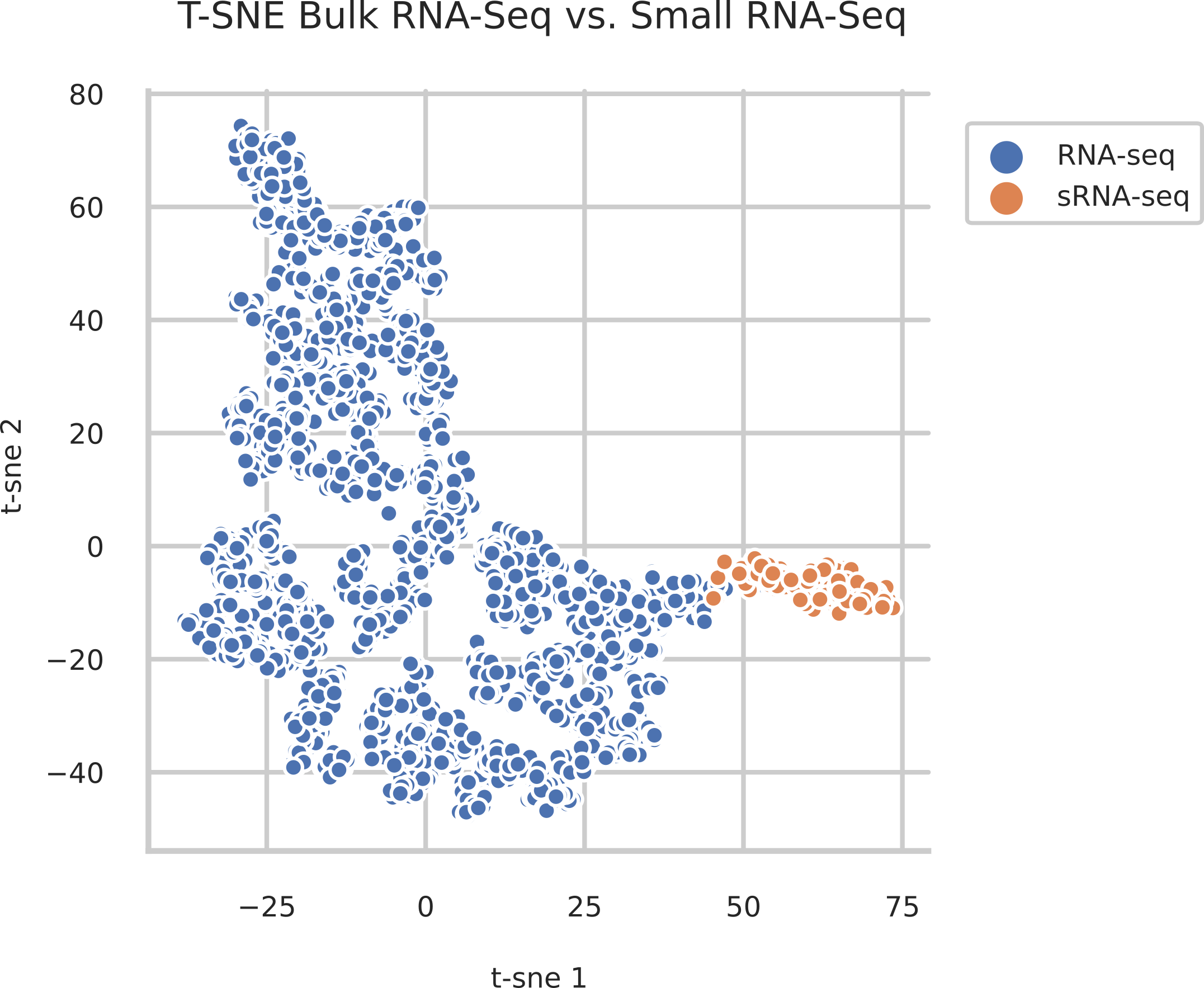

Supplement: giab064_Supplemental_Files [file giab064_supplemental_files.zip › figure_S2_srna_removal.png]

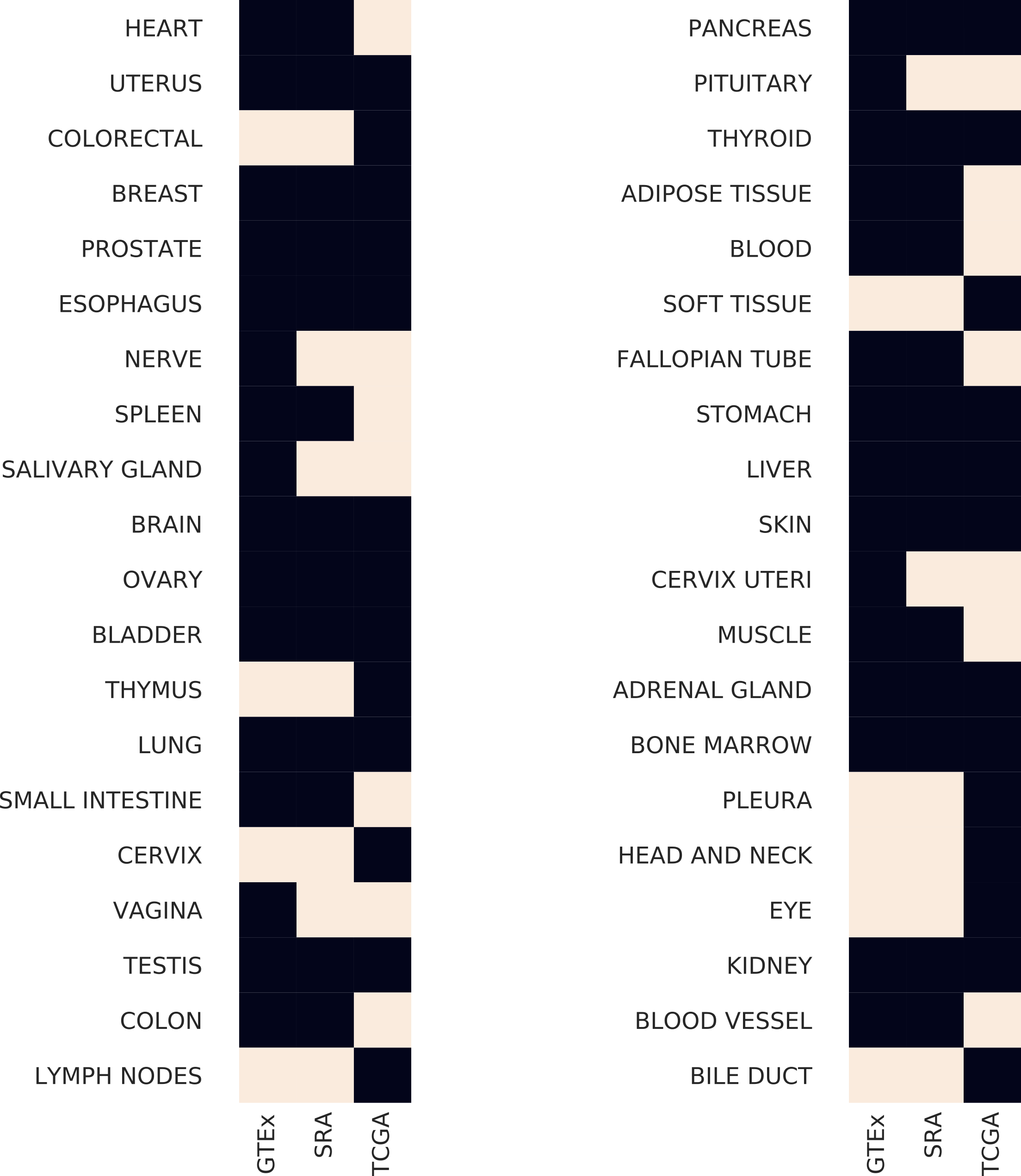

Supplement: giab064_Supplemental_Files [file giab064_supplemental_files.zip › figure_S3_tissue_overlap.png]

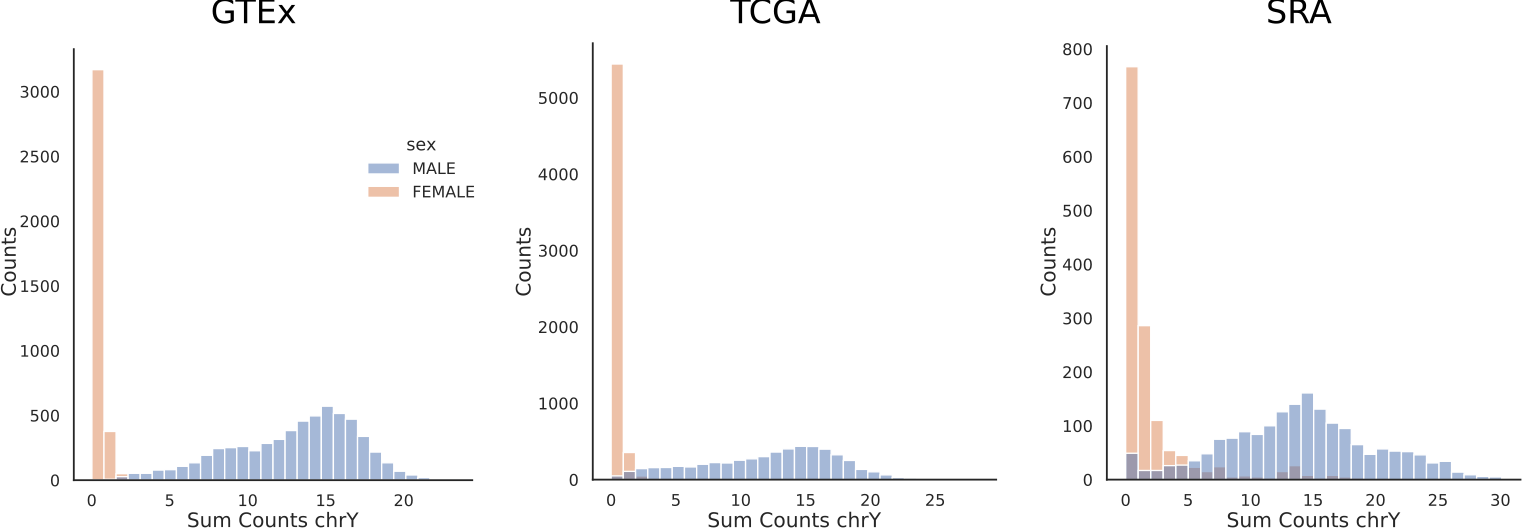

Supplement: giab064_Supplemental_Files [file giab064_supplemental_files.zip › figure_S4_sx_sumy.png]

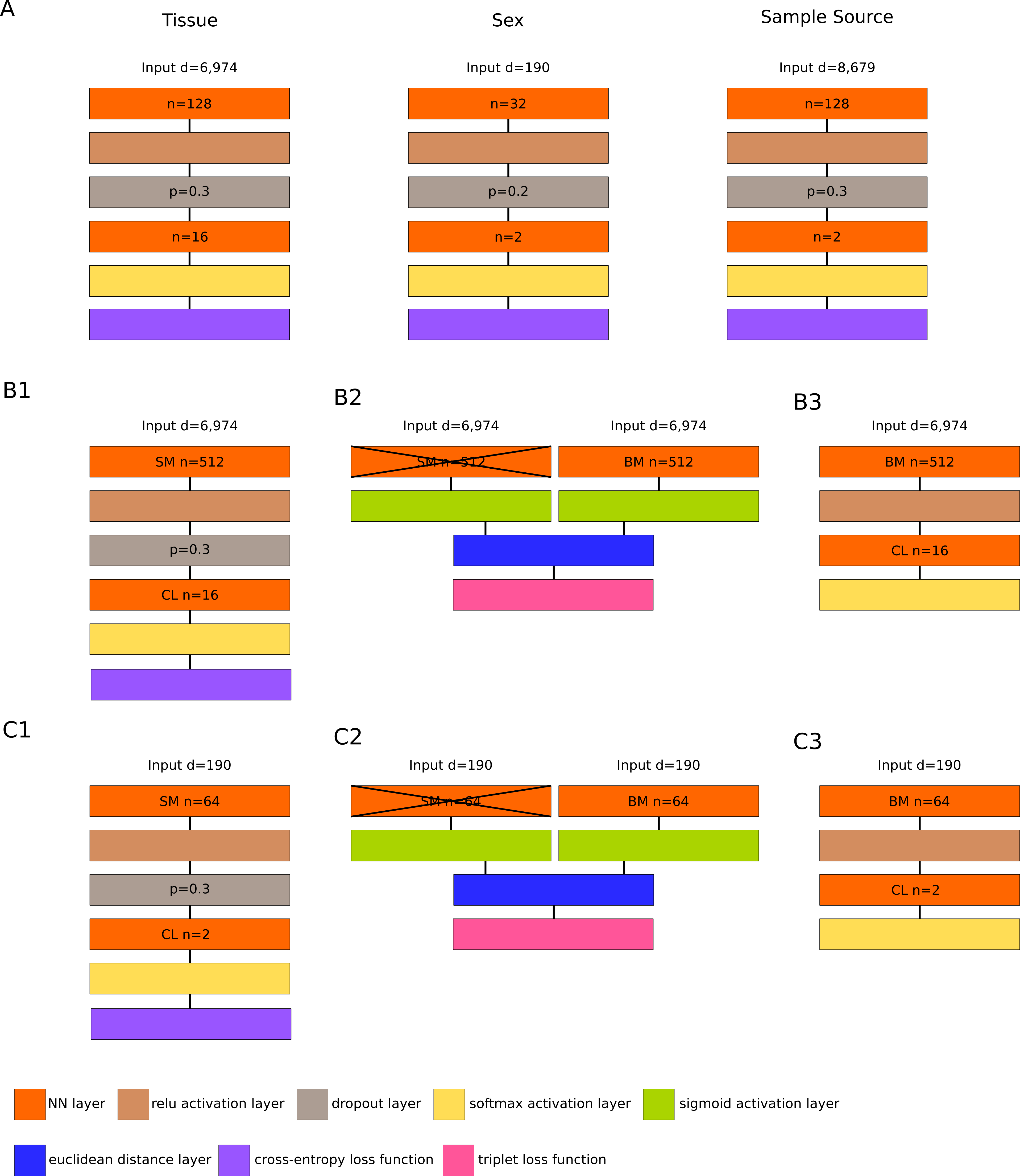

Supplement: giab064_Supplemental_Files [file giab064_supplemental_files.zip › figure_S5_model_config.png]

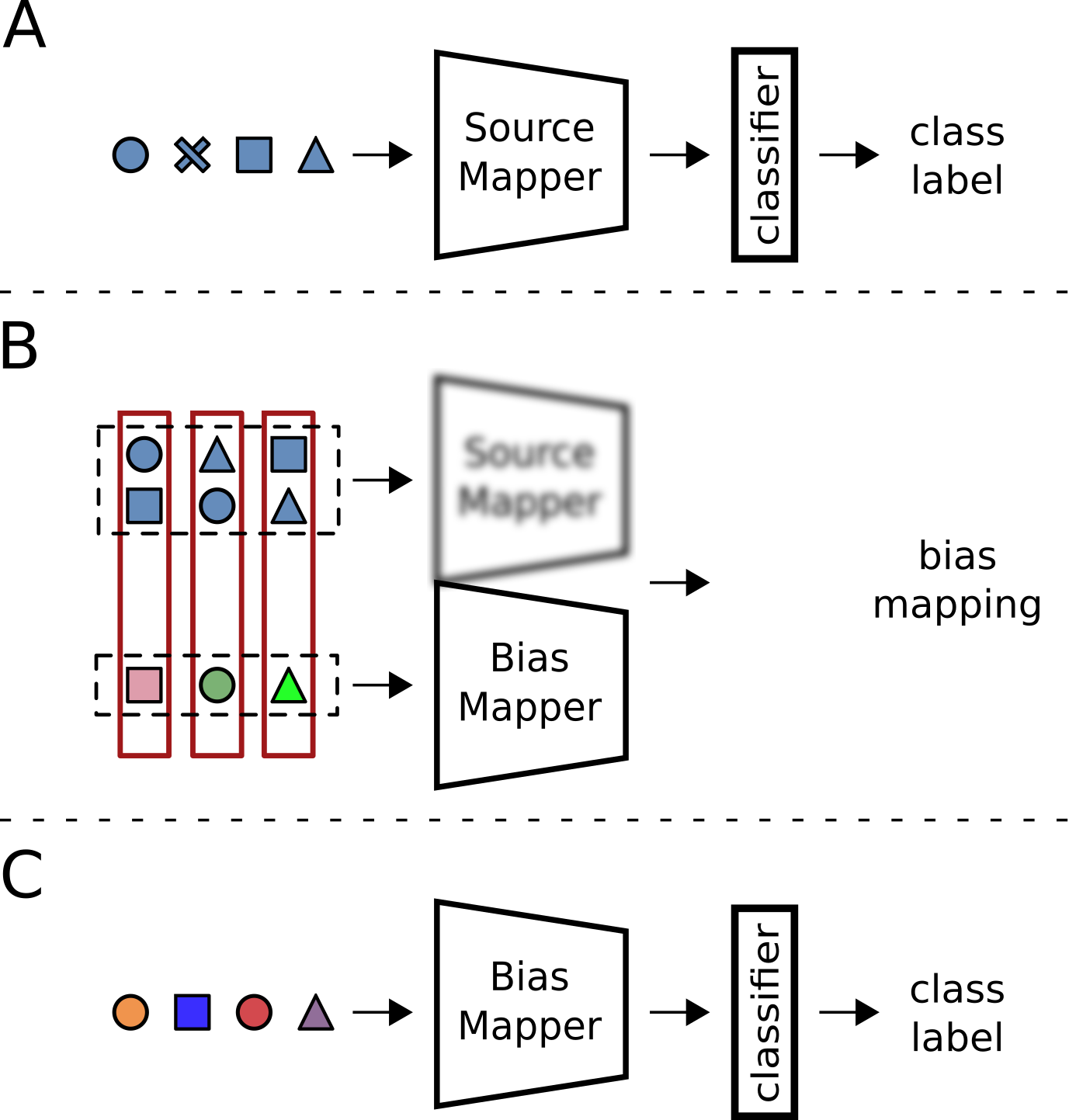

Supplement: giab064_Supplemental_Files [file giab064_supplemental_files.zip › figure_S6_da_short.png]

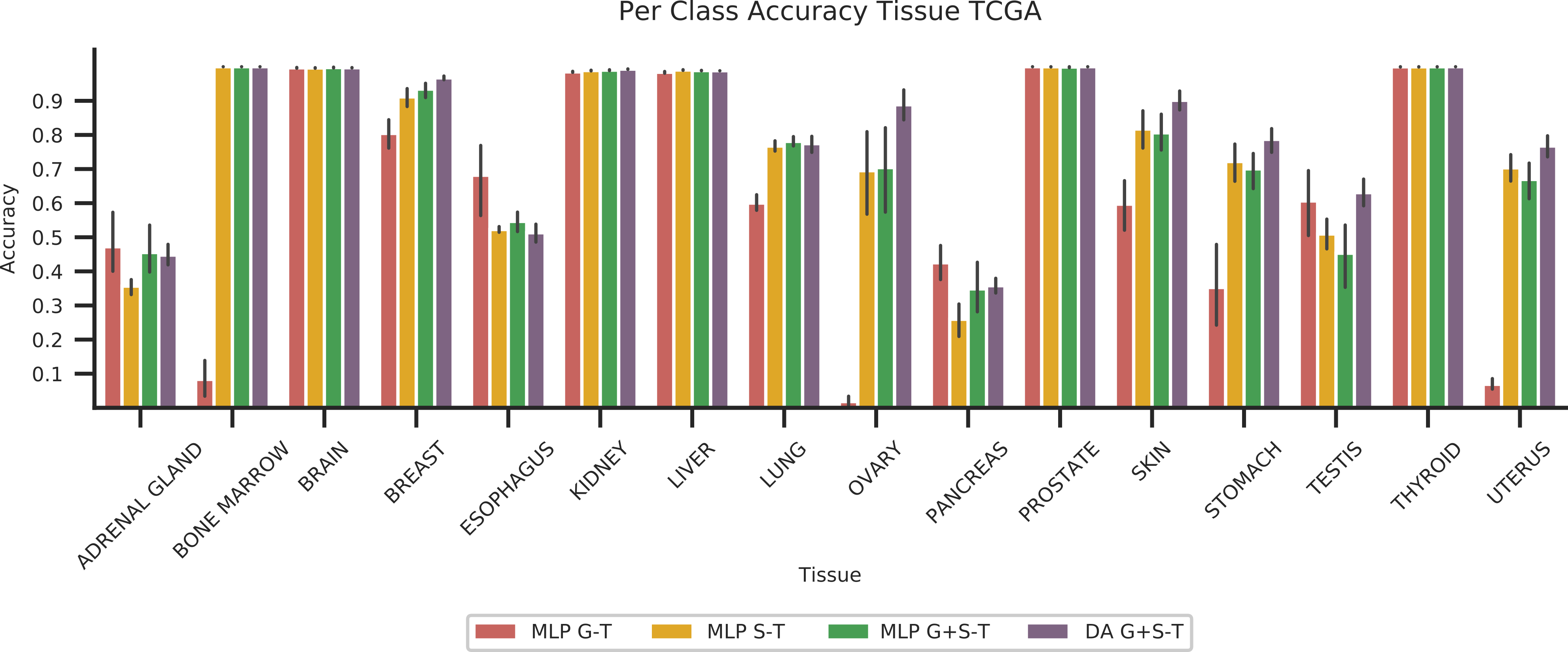

Supplement: giab064_Supplemental_Files [file giab064_supplemental_files.zip › figure_S7_per_class_accuracy.png]

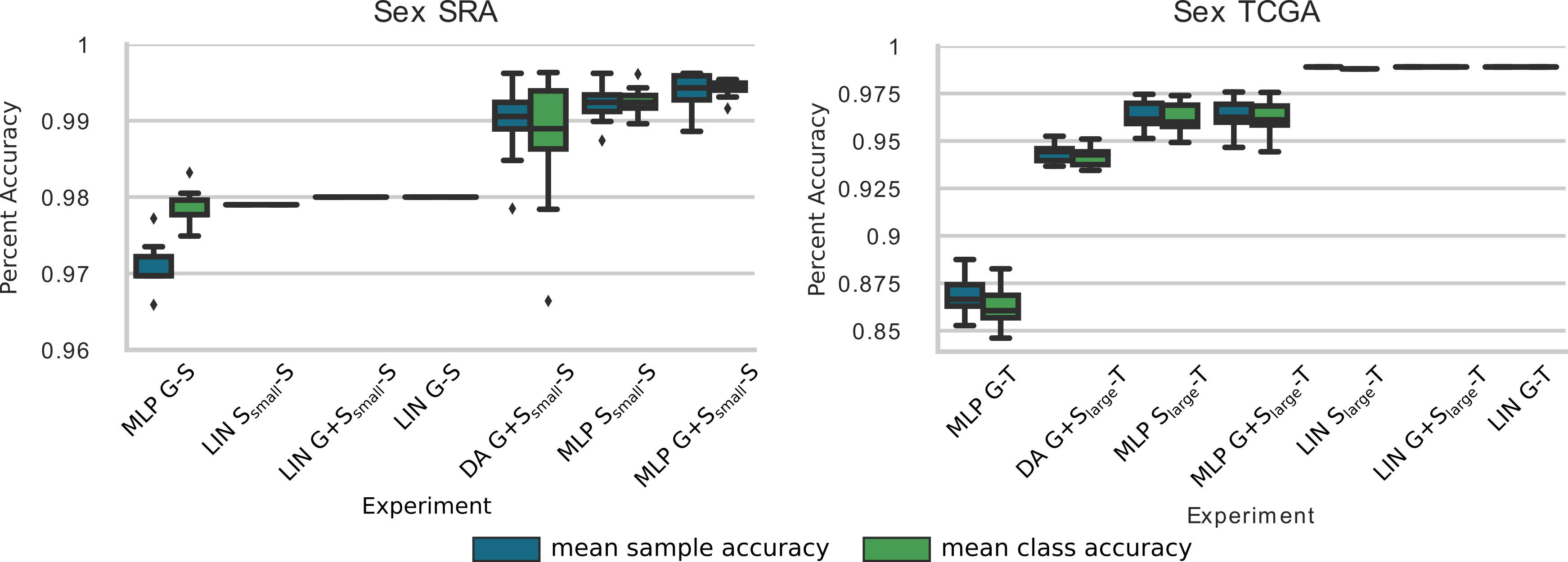

Supplement: giab064_Supplemental_Files [file giab064_supplemental_files.zip › figure_S8_sex_tcga.png]

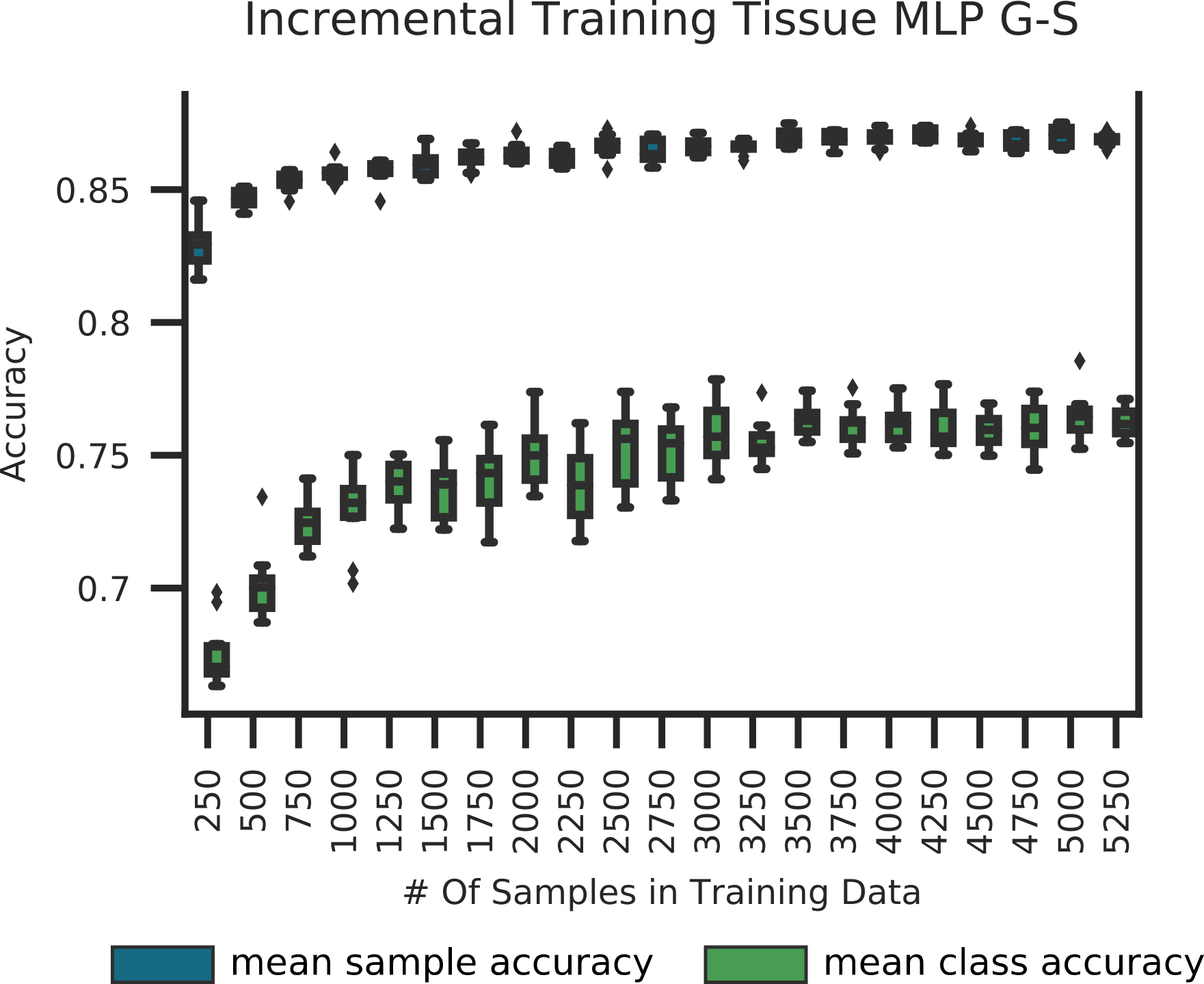

Supplement: giab064_Supplemental_Files [file giab064_supplemental_files.zip › figure_S9_mlp_g_s_inc.png]
