# Supplementary material for: Bias-invariant RNA-sequencing metadata annotation
Source: Gigascience. 2021 Sep 22;10(9):giab064. doi: 10.1093/gigascience/giab064 (PMC8559615; doi:10.1093/gigascience/giab064)
Supplement: giab064_Response_to_Reviewer_Comments_Original_Submission [file giab064_response_to_reviewer_comments_original_submission.pdf]

Author's Response To Reviewer Comments

Close

Rebuttal of ‘Bias invariant RNA-seq metadata annotation’

The reviewers raised a couple of very valid points of critique, especially with respect to potential overfitting of the ANN models. We addressed all concerns and believe that these changes significantly improved the scientific standard of the revised manuscript. In the end, we would like to thank the reviewers for their excellent suggestions, comments, and time.

In the PDF version, uploaded with the manuscript as "answers to reviewers" and tagged as "Supplementary Material", reviewer questions are in black font, our answers in blue font and text changes to the manuscript in black font in italics.

Reviewer reports:

Reviewer #1: In this manuscript, the authors address the task of phenotype prediction from gene expression data, with a focus on gene expression profiles measured via RNA-seq and the phenotypes of tissue, sample source (tissue biopsy or cell line culture), and sex. The primary motivation for the task is the improvement of metadata labeling RNA-seq samples, particularly in public databases such as the Sequence Read Archive (SRA), for which the metadata are often incomplete and unstandardized. Recently, a linear regression based approach was shown to be effective for this task (Ellis et al. 2018). This work explores the use of non-linear artificial neural networks (ANNs) as well as a "domain adaptation" (DA) training approach, which aims to reduce issues resulting from dataset-specific biases (also referred to as "batch effects"). The results of a series of thorough experiments involving phenotype prediction on SRA and TCGA samples indicate that the ANNs as well as the DA training approach improve upon the performance of the prior linear regression model. The authors then use their methods to provide phenotype labels for SRA samples missing this information.

I fully agree that improvements to the metadata for databases such as the SRA are important, both for more accurate retrieval of relevant datasets as well as for large-scale statistical meta-analyses or machine learning with data in these databases. I also agree that methods addressing dataset-specific biases, or batch effects, are critical in this context. Thus, the DA approach introduced in this manuscript is of great interest.

Overall, I found the manuscript to be well written and the experiments quite thorough. However, I have a few concerns regarding the evaluations that I believe need to be addressed in order for the accuracy improvements to be convincing.

Major comments:

1. A priori, I would expect prediction of sex from gene expression data would be a relatively trivial task using the counts of reads mapping to the X and Y chromosomes. Figure S1 confirms this expectation, at least for the GTEx and TCGA datasets: the male and female samples are easily, and linearly, separable. Thus, I was surprised that there were accuracy gains with the ANNs for this task. Looking at the right (SRA) panel in Figure S1, a major concern here is that the ground-truth sex labels on the SRA samples, which were used for the test set, are likely incorrect for a non-negligible number of samples. Because of this issue, it is possible that the ANNs are actually learning to predict such samples *incorrectly* in truth (but correctly with respect to the test labels). For example, perhaps the MetaSRA is systematically assigning an incorrect sex label to certain cell lines and the ANNs are then learning features of those cell lines that allow them to predict the sex correctly with respect to the MetaSRA label, but incorrectly in truth. A thorough investigation into the apparent performance gains for sex prediction would help to clear up this issue.

This is actually a great comment and yes, the assumption that the model might overfit if the training data would contain a considerable amount of false annotations is quite conceivable. We therefore first

performed an exploratory analysis of potential misannotations in the SRA dataset by investigating the range of chrY total sum counts per data source (see figure below).

Fig. S4. Misclassification in MetaSRA. Histogram of the total sum of normalized counts mapped to the chrY for GTEx, TCGA and SRA. Male and female clearly overlap in SRA, indicating mislabeling by MetaSRA.

The figure, which was added to the revised manuscript, shows histograms of all samples of GTEx, TCGA and SRA, respectively. Plotted are the sum counts on the chrY. The plot supports the notion that there are many FEMALE labeled samples that have a high chrY expression in the SRA data (and vica-versa). A threshold for chrY total count sum was chosen to clearly identify true labels. Because some GTEx and TCGA samples have a non-zero chrY sum count, we picked a threshold to define FEMALE at sum count chrY <= 2. Given this threshold we identified 366 of 3240 SRA samples labeled FEMALE to be above that threshold (i.e. they are probably MALE). Next we observed that 271 of these 366 samples were in the training set (FEMALE n=1,017, MALE n=1,246). If the model overfit on this wrongly labeled data, the trained model would predict the wrongly labeled training data wrong (i.e. with the training label FEMALE, and not with the likely correct label MALE). In other words, the potentially mis-annotated true male samples (falsely annotated as female) amount to 21.7% of all male samples (for females 25.3%). The DA model assigned 220 of these 271 training samples as FEMALE. We take this as evidence that the model overfit on the training data. In our answer to comment 2 by the reviewer we show that the ANN starts overfitting if >20% of the training data of a single class is incorrectly annotated, as is the case here. A similar observation was made for the samples annotated as MALE by MetaSRA that are above the chosen threshold.

To avoid overfitting of the model on wrong labels, we cleaned the SRA training and test set and removed all ambiguous samples according to the sum count chrY threshold stated above. This significantly changed the results for SEX phenotype prediction. For example, the DA model now predicts at 0.99 accuracy compared to 0.93 with the unfiltered training data. The difference in classification accuracy between the different models (LIN, MLP and DA) is now in the range of 1%. We still observed a small performance increase for the ANN models and decided to keep the phenotype in the study but gave it less weight by moving it into the supplementary figures. The SEX phenotype was removed from the main results figure 3 and merged with supplementary figure 8.
In the method subsection Phenotype Classification Experiments, we changed the paragraph about the sex phenotype from 'Sex: In total, 159 SRA studies contained samples annotated with male and or female by MetaSRA. These studies were combined into the training set (studies=78, n=2,317), and test set (studies=81, n=923) (Supplementary Tables 2 and 3). For model validation, GTEx was randomly split into training and test sets with an 80:20 ratio for both sex and tissue classification.' to 'Sex: We noticed SRA samples identified as female by MetaSRA to have a significant amount of reads mapped to chrY (Supplementary Figure 4). All samples labeled as female with a total normalized count >= 2 and all samples labeled male with a total normalized count <2 were removed. In total, 149 SRA studies contained samples annotated with male and or female by MetaSRA. These studies were combined into the training set (studies=73, n=2,017), and test set (studies=76, n=791) (Supplementary Tables 2 and 3). For model validation, GTEx was randomly split into training and test sets with an 80:20 ratio for both sex and tissue classification.' Supplementary Table 2,3 and 6 were adjusted according to the new data set sizes and experiment results.
As suggested by the reviewer, we then sought to correct potential mis-annotations of the MetaSRA data by predicting their labels using the model trained on the high-confidence annotations. We used the MLP G+S model to predict the true corrected label for the removed SEX samples. For 82% of the 132 filtered samples, the MLP model predicted the opposite of the presumably wrong MetaSRA labels. However, our MLP model was able to confirm the MetaSRA label for 24 samples. These samples had a mean chrY count sum of 2.4 (i.e. close to the cutoff value). We were able to manually confirm some samples. For example, SRR1164833, SRR1164787 and SRR1164842 are samples from a prostate cancer study labeled as MALE by MetaSRA. Our MLP model correctly classified these samples despite the fact that their chrY total sum count was between 0.4 and 1.4. On the other hand, SRR16076 54 / 56 / 61 / 62 / 64 / 65/ 70 / 71 are annotated as FEMALE by MetaSRA and the MLP but had a chrY total sum count of 2-5.3. Because the MLP is able to correctly classify these borderline cases, we are convinced that no overfitting on the training data is taking place.
These findings were added to the results section 'ANN Models Can Correct Mislabeling in MetaSRA', which summarizes the new results obtained answering the reviewers question 1-3 (paragraph shown as answer to question 2).

2. Related to comment #1, the same issue is also a concern for the prediction of tissue in the SRA, and potentially also for sample source. That is, if there are systematic annotation errors by the MetaSRA with respect to tissue of origin, the ANNs could actually be learning and propagating these systematic errors. Because the linear regression model is more limited, it is less able to learn such errors and is, in fact, more robust to them. In summary, the authors should provide some evidence that the presented performance gains are not largely due to learning such systematic label errors in the MetaSRA. Note that the MetaSRA is the result of an automated pipeline, not manual curation, so a certain fraction of errors are to be expected.

Again, a great comment and suggestion by the reviewer. We fully agree with the reviewer that a certain fraction of errors is to be expected in the MetaSRA annotation. An overfitting experiment was designed, to investigate the possibility that the ANN models overfit on wrongly annotated data. In a nutshell, the ANN models predict correctly when the level of mis-annotations in the training set does not exceed ~20%, above ~20% mis-annotations result in progressively increasing model overfitting. We added the following text to the methods section: '
Test for Overfitting
MetaSRA provides labels for SRA data generated in an automated way. We have identified mislabeled samples for the sex phenotype (see Methods). The following experiment was designed to test the ANN based model's susceptibility to overfitting on mislabeled training data. An MLP model was trained on GTEx data on four tissue classes (i.e., brain, esophagus, lung and skin). A range of fractions of the brain samples were randomly assigned to skin tissue (i.e., 0.01,0.025,0.05,0.1,0.20,0.5 and .8). The model was then trained on GTEx samples of the four classes, including the mislabeled brain samples. We tested the models overfitting capabilities by letting it predict the label of the mislabelled brain samples. If the model overfits, these samples should be predicted to be from skin tissue. The same experiment was conducted for the sex phenotype by mislabeling male samples as female.'. We also added a novel Fig. S10 to the manuscript, showing stable prediction performance of the ANNs with training data mis-labels of up to ~20%: '

Fig. S10. Test of Overfitting. An MLP model was trained on GTEx data. An increasing fraction of one class was assigned a wrong class label (e.g., brain to skin). The model was trained on the partially mislabeled data and the mislabeled data was predicted by the model after training. We quantify the model's susceptibility to overfitting by letting it correct the mislabeled training data. The MLP model was able to correct all mislabeled data up to a mislabeling fraction of 20%. We conclude that the ANN models are very robust in dealing with mislabeled data.

The following text was added to the results section: '
ANN Models Can Correct Mislabeling in MetaSRA
Given the difficulties with metadata standards in SRA data, mislabeling in MetaSRA is to be expected. To understand if and when ANN models would overfit on mislabelled MetaSRA data, we trained an MLP on partially mislabeled samples (see Methods). Supplementary Figure 10 shows that the MLP model correctly predicts brain samples, even if they were presented as skin samples during model training. A decrease of this accuracy was observed if more than 20\% of all brain samples were mislabeled as skin. A similar observation was made for the sex phenotype (Supplementary Figure 10). We concluded that our models are robust if less than 20\% mislabelled data is present during training. More importantly, these models can be used to correct mislabeled MetaSRA data.
In the specific case of sex classification, the MLP G+S was used to predict the true corrected label for the SEX samples that were removed from training due to low sex-chromosome counts (see Methods). For 82% of the 132 filtered samples, the MLP model predicted the opposite of the presumably wrong MetaSRA labels. However, our MLP model was able to confirm the MetaSRA label for 24 samples. These samples had a mean chrY count sum of 2.4 (i.e. close to the cutoff value). Manual confirmation revealed a high model accuracy. For example, SRR1164833, SRR1164787 and SRR1164842 are samples from a prostate cancer study labeled as MALE by MetaSRA. Our MLP model correctly classified these samples despite the fact that their chrY total sum count was between 0.4 and 1.4. On the other hand, SRR16076 54 / 56 / 61 / 62 / 64 / 65/ 70 / 71 are annotated as FEMALE by MetaSRA and the MLP but had a chrY total sum count of 2-5.3. We see the correct classification of these borderline cases as further evidence that no overfitting is taking place.
A list of all SRA samples for which the MetaSRA labels and the predicted labels mismatched is available in the Supplementary Material.'. We thank the reviewer for this great comment and hope that the revised manuscript builds a strong case for the stability of the approach taken.

3. Also continuing the line of thought from comments #1 and #2, an additional major application of phenotype prediction is *correction* of mislabeled samples, but this is not discussed in this manuscript. I don't think the authors necessarily need to demonstrate this application (and in fact they do briefly in the "Prediction of SRA Sex" section of the results), but a deeper analysis of this might go hand in hand with addressing comments #1 and #2.

Another valuable suggestion, which we have addressed in the answers to comments #1 and #2 (and the revised document). We really think that the first three main comments of the reviewer and our investigation into them strengthened the overall quality of the manuscript considerably.

4. An important contribution of this work is the set of newly-predicted phenotype labels. I cannot find mention of where this set can be accessed. Perhaps it can be archived at a site such as Zenodo, if it is not already.

We apologize if we did not make the newly-predicted phenotype labels available to the reviewer in the initial submission. We have now uploaded all supplementary data to gigadb.org, as requested by the editor. In addition, the data can now also be downloaded from the git page https://github.com/imsb-uke/rna_augment/tree/master/supplementary%20material

Minor comments:

5. In the introduction, the authors describe some prior DA approaches and then state that "All these methods have been implemented and applied by us for RNA-seq phenotype prediction and found not to be scalable to a situation with hundreds of different and scarce target domains, encountered, for instance, in the SRA." This would seem to be a result rather than a statement of prior facts, and should be moved to the results section (ideally with experiments), unless this was shown in a prior publication.

We completely agree that our work on other architectures that did not provide good results are results rather than background information. We fear, however, that expanding on these 'negative' results too much would further complicate and lengthen a manuscript that is already quite long and non-trivial. In agreement with the reviewer, we therefore added an extended text to the novel methods section 'Other Models': '
Other Models
While developing our DA model we did a thorough literature research and implemented and tested multiple architectures and strategies. Here we give a brief overview of the models we found not suitable for the problem of bias invariant RNA-seq metadata annotation. The first strategy that has been tested was interpolation between source and target domain by training feature extractors on an increasing ratio of target to source domain data. The second strategy was adversarial training by applying two loss functions. The first loss function forces the model to learn weights for the class prediction task, while the second forces the model to learn to ignore differences between the source and target domain. We also implemented Tzeng's [ref?] adaptation of this idea, proposing a model using a separate source and target encoder, using them as 'real' and generator input for a generative adversarial network that is capable of ignoring bias. These models ultimately failed due to the hundreds of dataset biases in the SRA data and their relatively small sample size (data not shown). For the case of scarce target data an approach was previously proposed using Siamese networks. The trained model achieved an msa of 0.83 and mca of 0.79 for tissue classification on SRA data. The mca achieved is comparable to the results of the MLP model, however, the msa score is 6% lower than even the LIN model. The more challenging task of learning to map the bias embedding into the pre-learned class embedding, as presented in this paper, finally resulted in the desired outcome.'. In addition, the beginning of the methods section 'Model Architecture' was changed from 'Our DA architecture is based on the Siamese network architecture.' to 'Our DA architecture is based on the Siamese network architecture. A Siamese network usually shares the weights between two equal networks, here however, we do not use weight sharing. Weight-sharing and other types of architecture did not prove to be applicable to this problem (see Methods section Other Models).' to reference the novel section. Again, we thank the reviewer for this excellent comment, which increased the quality of our manuscript.

6. At the beginning of the methods section, I found the phrase "which we define as the number of unique dataset biases present within one data source" confusing. Only later did I come to understand that this was simply referring to the number of studies. I think this could be made clearer earlier in the text. Also, that phrase references Fig S1, which shows the sex labels on the samples from each source, and doesn't really show the heterogeneity of the source. It is unclear why that figure is referenced here.

We thank the reviewer for pointing out this misleading reference. To correct this mistake, we have modified the beginning of the first paragraph of subsection "Data Acquisition". 'To train and test models we gathered data from three different sources, each with a different level of homogeneity, which we define as the number of unique dataset biases present within one data source (Supplementary Figure 1).' to 'To train and test models we gathered data from three different sources (i.e. GTEx, TCGA and SRA), each with a different level of heterogeneity (Supplementary Figure 1). We measure data source heterogeneity by the number of unique dataset (or studies) in the source. Each dataset (or study) is believed to have a unique bias.'
We also agree that the current supplementary figure does not visualize the data heterogeneity in the data sources sufficiently. We thus decided to replace it with the following figure:

Fig. S1. Visualizing Data Set Bias. GTEx is a single-study data source, while SRA is a multi-study data source. A) T-SNE plot of gene expression values of GTEx and B) SRA samples, belonging to five different tissues. The GTEx data is more coherently clustered compared to the SRA data. The individual studies in the SRA data appear to form less homogeneous clusters, indicating a larger within-variance in the data source.

We believe that these changes better reflect the data and claims of our study and thank the reviewer for the suggestions.

7. Sample source definition: I understand that the MetaSRA sample type classifications were used, but it is not clear to me how they were mapped to "biopsy" and "lab grown cell line" categories. It sounds like "tissue" was mapped to "biopsy" but I'm not sure about the rest. One MetaSRA category is "primary cells", which can be cells sorted from a disassociated (biopsied) tissue sample. Are those also considered "biopsy?"

We agree with the reviewer that there is potential for confusion between the phenotype tissue and the category TISSUE of phenotype sample source. Therefore, we renamed the MetaSRA category TISSUE to biopsy. Throughout the text (e.g. in the caption of Figure 1) we try to emphasize this by writing "tissue (e.g. lung, heart)". To make this even more clear, we added the following three sentences to the method section Phenotype Classification Experiments. Below we marked in red the added information:'
Phenotype Classification Experiments.
Tissue: To ensure that … For model validation GTEx was randomly split into training and test sets with an 80:20 ratio for both sex and tissue classification. Sample Source: A confidence cutoff of >= 0.7 was applied (provided by MetaSRA), reducing the total amount of annotated samples for SRA from 23,651 to 17,343. MetaSRA provided six different types of sample source. The two largest classes, TISSUE and CELL LINE were selected. In this study we renamed the MetaSRA label TISSUE to biopsy to not be confused with the phenotype tissue (e.g., heart, lung, skin). For each of the two selected categories we sorted all available studies by number of samples, placed the first third of studies into the training (studies=420, n=12,725), the second third into the test (studies=422, n=3,144) and the last third into the SRA validation set (studies=418, n=1,124) (Supplementary Tables 2 and 3). A list of the sample ids and corresponding labels is available in the Supplementary Material.'.
The reviewer also mentioned the MetaSRA term PRIMARY CELLS. It is true that the MetaSRA defines PRIMARY CELLS as a subtype of TISSUE in their hierarchical classification (supplementary figure 6, Bernstein et al. 2017). However, we believe that samples obtained from biopsies are split into altered and unaltered cells. The full name of the PRIMARY CELLS label is PRIMARY SPECIALIZED CELLS which would indicate some kind of alteration to the sample. PRIMARY CELLS and the other 3 categories that are not TISSUE or CELL LINE were thus mapped to "others" (the "catch-all") class during the annotation phase. We now specify this in the result section "Prediction and Availability of Novel Metadata" with the following sentence: 'Specifically, we first trained a new MLP model to identify the sample source biopsy vs. all other sample sources available in the SRA data as defined by MetaSRA.'. We hope that these changes help readers understand the terminology used in this manuscript.

8. What was used for "gene length" to normalize to TPM?

We extracted the 'gene length' from Gencode v25, GRCh38, 07.2016. We have integrated this information in the revised document section Dimensionality Reduction and Normalization 'First standard log2 Transcript per Million (TPM) normalization was applied to normalize for gene length (Gencode v25,

GRCh38, 07.2016) and library size.'.

9. "Metadata annotation" section: I did not understand the phrase "no samples were discharged because of their tissue label." Are samples being removed from the training or test sets with some criteria?

This sentence is indeed hard to understand. In brief, we downloaded ~50,000 SRA samples from recount2 and selected the samples with a tissue label belonging to the 16 classes. For the annotation we take all samples. The samples not belonging to one of the 16 tissues goes into a specially "catch-all" class.
In the revised document we have removed 'no samples were discharged because of their tissue label.' from the paragraph as we believe the next sentence 'Samples from a tissue class other than the original 16 classes were pooled together into a 'catch-all' class, resulting in 17 classes.' makes the point perfectly clear. We hope that this change adequately addresses the reviewer's justified critique.

10. DA model architecture: the text says that the model "is trained on semi-hard triplets" and then gives a definition of this based on Euclidean distances in the embedding spaces. This confuses me because bias embedding mapper (BM) is what is being trained here, so this appears circular. How do you get the distances without already having the BM?

We are sorry for not clarifying this better in the first manuscript. The BM is pretrained on the source domain, as it is a direct copy of the trained SM. We have changed 'For a second training cycle, the bias mapper is created with the same architecture as the SM. The CL is removed and the weights of the SM are frozen. Triplets of data are forward propagated through the BM and SM in parallel (Figure 2C).' to 'For the second training cycle, the SM and the CL are separated and their weights frozen. Frozen weights are not updated during the second training cycle. The bias mapper is created by copying the architecture and weights of the trained source mapper. SM and BM are trained on triplets drawn from the source and the bias domain. Samples from the source domain are passed through the SM, samples from the bias domain through the BM at the same time (Figure 2C).' in the revised document. In addition, we changed 'Where d(i,j) are the distances in embedding space between the respective outputs of the BM and SM on samples i and j.' to 'Where d(i,j) are the distances between the constricted embedding space of the SM and the bias mapping into that space of the BM on samples i and j.'.
We also added the new section 'Other Models' to the methods, introducing the msa and mca achieved with a Siamese network where weights are shared between the SM and BM. (see response to minor comment 5).

11. Figure 3D: y-axis appears to be mislabeled

We thank the reviewer for pointing out this mistake. In the revised document, we have corrected the y-axis labeling of what is now Figure 3C.

Reviewer #2: Summary:
The authors present a Domain adaptation model that uses a Siamese network architecture to lead missing metadata from bulk RNAseq data and compare the performance to a previously published linear regression model (LIN) and a multilayer perceptron (MLP). As data sources, the authors used GTEx, SRA, TCGA. The DA model outperforms the LIN and MLP, when many classes to learn (e.g. in case of tissues), but not in the case of sex and sample source. While the authors present their work in a concise and clear way, I think that they can improve their manuscript in several points.

Major:

I did not see a cross-validation of any of the used models. Instead, the authors varied the random seeds for model initialization. While I appreciate the split by study in the case of the SRA data, I think that the authors should add a cross-validation approach on the model training to increase robustness, even if that means that the training dataset has varying size.

Overfitting is a pivotal concern in any Machine Learning task, and we believe the reviewer addresses a very critical point. Domain adaptation methods such as Siamese Neural Networks have been developed to overcome the problem of overfitting, by learning latent space representations that reliably map two (or more) biases to the same manifold.
To understand if and when our ANN models start to overfit, we chose to simulate mis-labelled data in the training sets of the models and subsequently observed 'overfitting' of the model based on 'wrong' predictions (predicting the mis-annotated class of the data).

In a nutshell, the ANN models predict correctly when the level of mis-annotations in the training set does not exceed ~20%, above ~20% mis-annotations result in progressively increasing model overfitting. We added the following text to the methods section: '
Test for Overfitting
MetaSRA provides labels for SRA data generated in an automated way. We have identified mislabeled samples for the sex phenotype (see Methods). The following experiment was designed to test the ANN based model's susceptibility to overfitting on mislabeled training data. An MLP model was trained on GTEx data on four tissue classes (i.e., brain, esophagus, lung and skin). A range of fractions of the brain samples were randomly assigned to skin tissue (i.e., 0.01,0.025,0.05,0.1,0.20,0.5 and .8). The model was then trained on GTEx samples of the four classes, including the mislabeled brain samples. We tested the models overfitting capabilities by letting it predict the label of the mislabelled brain samples. If the model overfits, these samples should be predicted to be from skin tissue. The same experiment was conducted for the sex phenotype by mislabeling male samples as female.'. We also added a novel Fig. S10 to the manuscript, showing stable prediction performance of the ANNs with training data mis-labels of up to ~20%: '

Fig. S10. Test of Overfitting. An MLP model was trained on GTEx data. An increasing fraction of one class was assigned a wrong class label (e.g., brain to skin). The model was trained on the partially mislabeled data and the mislabeled data was predicted by the model after training. We quantify the model's susceptibility to overfitting by letting it correct the mislabeled training data. The MLP model was able to correct all mislabeled data up to a mislabeling fraction of 20%. We conclude that the ANN models are very robust in dealing with mislabeled data.

The following text was added to the results section: '
ANN Models Can Correct Mislabeling in MetaSRA
Given the difficulties with metadata standards in the SRA state above, mislabeling in MetaSRA is to be expected. We designed an overfit test for the ANN models where we trained an MLP on partially mislabeled samples (see Methods). Supplementary Figure 10 shows that the MLP model correctly predicts brain samples, even if they were presented as skin samples during model training. A decrease of this accuracy was observed if more than 20% of all brain samples were mislabeled as skin. A similar observation was made for the sex phenotype (Supplementary Figure 10). We concluded that our models can be used to correct mislabeled MetaSRA data.
In the specific case of sex classification, the MLP G+S was used to predict the true corrected label for the removed SEX samples. For 82% of the 132 filtered samples, the MLP model predicted the opposite of the presumably wrong MetaSRA labels. However, our MLP model was able to confirm the MetaSRA label for 24 samples. These samples had a mean chrY count sum of 2.4 (i.e. close to the cutoff value). Manual confirmation revealed a high model accuracy. For example, SRR1164833, SRR1164787 and SRR1164842 are samples from a prostate cancer study labeled as MALE by MetaSRA. Our MLP model correctly classified these samples despite the fact that their chrY total sum count was between 0.4 and 1.4. On the other hand, SRR16076 54 / 56 / 61 / 62 / 64 / 65/ 70 / 71 are annotated as FEMALE by MetaSRA and the MLP but had a chrY total sum count of 2-5.3. We see the correct classification of these borderline cases as further evidence that no overfitting is taking place.
A list of all SRA samples for which the MetaSRA labels and the predicted labels mismatched is available in the Supplementary Material.'. We thank the reviewer for this great comment and hope that the revised manuscript builds a strong case for the stability of the approach taken. While we have not used a cross-validation approach to assess and minimize model overfitting, we hope that our results are compelling enough to convince the reviewer of the robustness of our approach. One of the reasons we chose not to go via cross-validation is the training set size differences that would make cross-validation results hard to compare (as the author also correctly stated). On another note, it might be interesting for the reviewer to also read our response to reviewer 1's first main concern (overfitting of sex annotations), which further proves the validity of the reviewer's excellent comment.

Page 8: The introduction of the "percentage point" (ppt) as metric is superfluous. I suggest to use percent (%) instead, if the authors really want to state relative changes. Further, I have the impression that ppt and % are used synonymously, so the authors should use the unit consistently.

We apologize for this unnecessary confusion. The ppt was dropped, and the result section was changed accordingly.

Figure 3: Plotting the relative change to the baseline model overrates the actual improvement of the DA approach and is not statistically sound. I suggest to state absolute changes in accuracy (or mca/msa)

instead.

We thank the reviewer for pointing out this problem. In the revised manuscript, we have changed figure 3 to show absolute accuracy. To highlight changes, we took the liberty to rescale the X-axis from ~0.6 to 1. In case the reviewer deems it necessary to show the full spectrum from 0 to 1 we would be more than willing to adjust the X-axis.

Statistical analysis page 8: When using a t-test, the authors assume that the msa/mca scores would be normally distributed around some unknown mean value. By looking at the boxplots, I think that the assumption is not necessarily true and I suggest to use a non-parametric test (e.g. Wilcoxon rank sum test) instead of the t-test.

This is an excellent observation, and we have replaced the t-test in the Prediction of SRA Tissue section with a non-parametric Mann-Whitney test and changed the text of the revised manuscript accordingly.

The method subsection Statistical Tests was changed from 'Accuracy distributions for sex and tissue prediction were tested for statistically significant differences using a t-test (two distributions, scipy.stats.ttest_ind v 1.3.1) or ANOVA (more than two distributions, scipy.stats.f_oneway) with a significance threshold of 0.01.' to 'Accuracy distributions were tested for significance using the non-parametric Mann-Whitney-U-Test (scipy.stats.mannwhitneyu v 1.3.1).'

Identifying novel training data (page 10): I would be very careful in including predicted labels as ground truth data (which is known as data imputation task). In these cases, retraining a classifier on both ground truth and predictions will lead to overfitting and spurious results, when the predicted labels dominate the ground truth labels.

The reviewer states that re-training on predicted data could result in overfitting, which is per se a valid concern. We would like to highlight, however, that 'E/M-like' approaches that train iteratively on 'harder' samples have shown to give superior classification and regression performances in several contexts. We agree that we haven't shown beyond reasonable doubt that in this case the re-training is not overfitting, which is why we have decided to remove this last paragraph. We thank the reviewer for raising this very good point of critique.

Minor:

Page 4: Gene selection based on Gini index: was there an overlap of the genes used for tissue and sample source classification? I would assume so from the setup with the range of Gini indices.

This is an interesting question, which we followed up upon. A list of all used genes per phenotype is now available for download at giga.db. The file input_features.xlsx contains the list of genes used for each phenotype and the intersection between the phenotypes. The sex and sample source phenotype share 166 input features, sex and tissue phenotype 155, and sample source and tissue share 2976 input features.

Figures and Subfigures are not fully in the order of first appearance (esp. subfigures 1 B-C compared to figure 2).

Unfortunately, we cannot find the noted inconsistent labeling of Figure 1 (B) compared to Figure 2.

Figure 1A: Datasets could be visualized as bar charts to give the reader an idea about the dataset sizes. Exact numbers can be stated in the supplement or figure legend

While we agree with the reviewer on almost every other point raised, we beg to differ here. We think that the number of samples per data source is of secondary importance in this study, it is rather the number of biases in the training data that is of utmost relevance. In case the reviewer and the editor insist on the suggested change, however, we will comply.

Figure 1: The figure design is clean, however, the green TCGA box is not colorblind friendly and difficult to distinguish from the purple GTEx box. Consider a lighter color.

We thank the reviewer for this valid comment, we have changed the TCGA box to yellow in the revised

manuscript.

Figure 3:
I struggle to understand the plot, especially the numbers at the top of each boxplot (please clarify in the figure legend, that this is msa and mca).
What was the baseline model in Figure 3D? Why do you present this as main figure, when the relative changes are within 1% (and therefore within the range of the noise level)?

We have significantly revised the figure and legend, in accordance with the reviewer's excellent suggestions and valid points of critique.

Supplementary Figure 7A:
The PCA analysis of the ovary data is interesting. The differences of TCGA-ovary and GTEx-ovary data is reflected in PC1 and I was wondering whether the authors could give a more detailed explanation on the reasons for this systematic bias, e.g. by analysing the loadings of PC1.

We agree with the reviewer, that the observed shifts in the GTEx and TCGA domain are of great interest and it could be informative to scrutinize the loadings of the respective PC1. Unfortunately, we did not find any biological pathway or category that might be enriched in PC1 when performing a PANTHER pathway enrichment analysis on the top 1% and top 5% genes (ranked according to absolute loading). Also, just looking at the genes themselves did not yield any insights to us, which might be due to the fact that we do not possess enough biological understanding of the matter. Therefore, we were not able to come up with any biological explanation of the shift between TCGA-ovary and GTEx-ovary data in PC1.
We would like to stress, however, that our manuscript is already quite large and complex and we fear that a closer inspection of genes and pathways responsible for cluster differences might be outside the scope of this study. We will include this information into a revised version if the reviewer or editor deems it relevant.

Page 9: Clarify. "For example, SRP056612 is a study on the effect of the coronavirus on cultured kidney and
lung cells [39] and SRP045611 is a study involving HEK cells, which lack the Y chromosome but are annotated as male by MetaSRA [40]."
As far as I understood the cited reference, it corresponds to the MERS coronavirus.

We thank the author for noting this and we corrected this in the revised manuscript.

Second, HEK cells are (most likely) of female origin, therefore, clearly state the nature of the mislabeling (I consider this as human error in the MetaSRA).

We completely agree with the reviewer that the annotation error is in the MetaSRA data. The nature of the error is either, as the reviewer suggested, a human error during submission, or a mapping error of the MetaSRA pipeline. Since we don't know which of the two is the problem we opted to not state the nature of the error in more detail.

By the way, line numbers would have been nice to comment on certain passages.

We absolutely agree with the reviewer, line numbers can greatly facilitate the review process. We tried to adhere to the GIGA Science submission guidelines, which unfortunately do not mention line numbers.

Close
