# Supplementary material for: Bias-invariant RNA-sequencing metadata annotation
Source: Gigascience. 2021 Sep 22;10(9):giab064. doi: 10.1093/gigascience/giab064 (PMC8559615; doi:10.1093/gigascience/giab064)
Supplement: giab064_Response_to_Reviewer_Comments_Revision_1 [file giab064_response_to_reviewer_comments_revision_1.pdf]

Author's Response To Reviewer Comments

Close

Reviewer #1: With this revised manuscript the authors have addressed the majority of my previous comments. I have a handful of new or remaining comments:

Major comments:

1. Overall claims made in the abstract and a couple of other places in the manuscript that the DA algorithm specifically outperforms the linear regression and traditional neural network models are either misleading or incorrect. What *is* clear is that training on heterogenous (or "multiple bias" in the terminology of the authors) datasets results in improvements when testing on samples from other datasets. I consider this to be the main finding of this work. The authors should rephrase their main findings to make this clear and not overstate the performance of the DA model. In particular, the abstract states:

"We present a deep-learning based domain adaptation algorithm for the automatic annotation of RNA-seq metadata. We show how our algorithm outperforms existing linear regression based approaches as well as traditional neural network methods for the prediction of tissue, sample source, and patient sex information across several large data repositories."

This statement is not supported by the manuscript. For sample source, DA is not evaluated, so it cannot have outperformed the other methods on that task. For patient sex, a traditional neural network trained on heterogenous data outperforms DA on both SRA and TCGA. For tissue, DA does have better accuracy than the other methods for TCGA data. For SRA tissue data, DA is comparable to a traditional neural network trained on heterogenous data.

We completely agree with the reviewer and changed the abstract accordingly. We changed the sentence in the abstract "We show how our algorithm outperforms existing linear regression based approaches as well as traditional neural network methods for the prediction of tissue, sample source, and patient sex information across several large data repositories." to "We show, in multiple experiments, that our model is better at integrating heterogeneous training data compared to existing linear regression-based approaches, resulting in improved tissue type classification."

In addition, we changed the following sentence form the last paragraph of the introduction. "Importantly, we find that our DA network significantly outperforms the strongly supervised LIN model by up to 15.7% in prediction accuracy." to "Importantly, we find that our DA network is able to integrate heterogeneous training data such that classification accuracy is up to 15.7% higher for tissue classification compared to the supervised LIN model."

We believe that all other sections of the text state the benefits of DA correctly, but would be more than willing to change any other overstatement that evaded our scrutiny.

2. The new experiments that test the neural networks' robustness to mislabeled training data are most welcome. However, I think it is important to note that mislabeling is likely not random, especially if it arises as an error in the MetaSRA's automated pipeline. Unlike random errors, systematic errors create signals in the training data that a model can learn and then replicate on test data. I am not suggesting that the authors perform new experiments (which may be challenging in this case). Rather, I am suggesting that the authors discuss this point.

We thank the reviewer for this great comment. It is correct that we can only approximate a systematic error in the training data. As suggested by the reviewer, we have changed our discussion from "We showed that our models are robust to overfitting, if up to 20% of the training samples per class are mislabeled. Our models are able to predict the correct class of a sample, even if the sample was mislabeled during model training. This property of our models was exploited for the correction of

wrongly annotated metadata in the MetaSRA and made publicly available.” to “A major concern with our experiments is the potential misclassification in the MetaSRA-annotated ground truth. The MetaSRA pipeline serves mainly as a normalizer for already existing metadata, and is therefore susceptible to human error. Systematic annotation errors create signals in the training data that a model can learn and then replicate on the test set. We approximated a systematic error by randomly mislabeling training data from a single class. We showed that our models are robust to overfitting, if up to 20% of the training samples per class are mislabeled. Our models are able to predict the correct class of a sample, even if the sample was mislabeled during model training. This property of our models was exploited for the correction of wrongly annotated metadata in the MetaSRA and made publicly available. ”.

Minor comments:
3. The definition of how "gene length" is determined is still unclear. The manuscript cites the Gencode annotation as providing gene length, but this is still not clear. Gene length is not a well-defined concept because each isoform of a gene can have a different length, and the isoforms of a gene can be expressed at very different levels. For a given RNA-seq sample, the accepted way to define "gene length" is to use the expression-weighted average of the gene's isoform lengths.

In principle, we agree with the reviewer that “gene length” is an ambiguous concept. However, here we use “gene length” for TPM normalization. The first step in TPM normalization is to calculate the reads per kilobase (RPK) values. To this end, the counts for each gene are divided by the gene length in kilobases. The count tables we downloaded from Recount2 were created by mapping the raw reads to the gene versions defined in Gencode v25, GRCh38, 07.2016. These “genes” are defined by a start and end position on the respective strands of the chromosomes. The length of the sequence the reads are mapped to is therefore exactly defined by Gencode v25, GRCh38, 07.2016.

4. In a number of places in the manuscript there are statements about the MetaSRA mislabeling samples. It should be made clear that the MetaSRA automated pipeline serves only to *standardize* the metadata in SRA (with the exception of sample source, which is predicted). There may be many errors in the raw metadata, and these will simply be standardized by the MetaSRA. Of course, it is also possible that the standardization process is also introducing errors. However, I think it is important to note the various ways in which these errors can arise.

We agree with the reviewer, which is why we have added the sentence “The MetaSRA annotations serve mainly as a normalizer for already existing metadata, and are therefore susceptible to human error.” to the discussion of the revised manuscript (also see answer to comment 2).

5. The definition of the triplets for training the BM is still a bit unclear. Are these triplets defined *once* at the beginning of training when the BM and SM have the same weights? Or is the triplet set updated during the training procedure as the BM weights change?

We appreciate this very good question and agree that this is not clear yet. To answer the question, we added the following sentence to the methods section Domain Adaptation Model - DA subsection Model Architecture: “Triplets are mined online, meaning that they are newly generated for each batch \cite{schroff2015}.”.

Close
