# Supplementary material for: Bias-invariant RNA-sequencing metadata annotation
Source: Gigascience. 2021 Sep 22;10(9):giab064. doi: 10.1093/gigascience/giab064 (PMC8559615; doi:10.1093/gigascience/giab064)
Supplement: giab064_Response_to_Reviewer_Comments_Revision_2 [file giab064_response_to_reviewer_comments_revision_2.pdf]

Author's Response To Reviewer Comments

Close

Dear Editor,

As requested, we added the references to the Giga Database and the git repository.
We re-checked the author's instructions and hope that everything is in order.

Kind Regards

Hannes Wartmann

Close
