# Supplementary material for: Bias-invariant RNA-sequencing metadata annotation
Source: Gigascience. 2021 Sep 22;10(9):giab064. doi: 10.1093/gigascience/giab064 (PMC8559615; doi:10.1093/gigascience/giab064)
Supplement: giab064_Reviewer_1_Report_Original_Submission — Colin Dewey -- 3/15/2021 Reviewed [file giab064_reviewer_1_report_original_submission.pdf]

Reviewer Report

Title: Bias invariant RNA-seq metadata annotation

Version: Original Submission **Date:** 3/15/2021

Reviewer name: Colin Dewey

Reviewer Comments to Author:

In this manuscript, the authors address the task of phenotype prediction from gene expression data, with a focus on gene expression profiles measured via RNA-seq and the phenotypes of tissue, sample source (tissue biopsy or cell line culture), and sex. The primary motivation for the task is the improvement of metadata labeling RNA-seq samples, particularly in public databases such as the Sequence Read Archive (SRA), for which the metadata are often incomplete and unstandardized. Recently, a linear regression based approach was shown to be effective for this task (Ellis et al. 2018). This work explores the use of non-linear artificial neural networks (ANNs) as well as a "domain adaptation" (DA) training approach, which aims to reduce issues resulting from dataset-specific biases (also referred to as "batch effects"). The results of a series of thorough experiments involving phenotype prediction on SRA and TCGA samples indicate that the ANNs as well as the DA training approach improve upon the performance of the prior linear regression model. The authors then use their methods to provide phenotype labels for SRA samples missing this information.

I fully agree that improvements to the metadata for databases such as the SRA are important, both for more accurate retrieval of relevant datasets as well as for large-scale statistical meta-analyses or machine learning with data in these databases. I also agree that methods addressing dataset-specific biases, or batch effects, are critical in this context. Thus, the DA approach introduced in this manuscript is of great interest.

Overall, I found the manuscript to be well written and the experiments quite thorough. However, I have a few concerns regarding the evaluations that I believe need to be addressed in order for the accuracy improvements to be convincing.

Major comments:

1. A priori, I would expect prediction of sex from gene expression data would be a relatively trivial task using the counts of reads mapping to the X and Y chromosomes. Figure S1 confirms this expectation, at least for the GTEx and TCGA datasets: the male and female samples are easily, and linearly, separable. Thus, I was surprised that there were accuracy gains with the ANNs for this task. Looking at the right (SRA) panel in Figure S1, a major concern here is that the ground-truth sex labels on the SRA samples, which were used for the test set, are likely incorrect for a non-negligible number of samples. Because of this issue, it is possible that the ANNs are actually learning to predict such samples *incorrectly* in truth (but correctly with respect to the test labels). For example, perhaps the MetaSRA is systematically assigning an incorrect sex label to certain cell lines and the ANNs are then learning features of those cell lines that allow them to predict the sex correctly with respect to the MetaSRA label, but incorrectly in truth. A thorough investigation into the apparent performance gains for sex prediction would help to clear up this issue.

2. Related to comment #1, the same issue is also a concern for the prediction of tissue in the SRA, and potentially also for sample source. That is, if there are systematic annotation errors by the MetaSRA with respect to tissue of origin, the ANNs could actually be learning and propagating these systematic errors. Because the linear regression model is more limited, it is less able to learn such errors and is, in fact, more robust to them. In summary, the authors should provide some evidence that the presented performance gains are not largely due to learning such systematic label errors in the MetaSRA. Note that the MetaSRA is the result of an automated pipeline, not manual curation, so a certain fraction of errors are to be expected.

3. Also continuing the line of thought from comments #1 and #2, an additional major application of phenotype prediction is *correction* of mislabeled samples, but this is not discussed in this manuscript. I don't think the authors necessarily need to demonstrate this application (and in fact they do briefly in the "Prediction of SRA Sex" section of the results), but a deeper analysis of this might go hand in hand with addressing comments #1 and #2.

4. An important contribution of this work is the set of newly-predicted phenotype labels. I cannot find mention of where this set can be accessed. Perhaps it can be archived at a site such as Zenodo, if it is not already.

Minor comments:

5. In the introduction, the authors describe some prior DA approaches and then state that "All these methods have been implemented and applied by us for RNA-seq phenotype prediction and found not to be scalable to a situation with hundreds of different and scarce target domains, encountered, for instance, in the SRA." This would seem to be a result rather than a statement of prior facts, and should be moved to the results section (ideally with experiments), unless this was shown in a prior publication.

6. At the beginning of the methods section, I found the phrase "which we define as the number of unique dataset biases present within one data source" confusing. Only later did I come to understand that this was simply referring to the number of studies. I think this could be made clearer earlier in the text. Also, that phrase references Fig S1, which shows the sex labels on the samples from each source, and doesn't really show the heterogeneity of the source. It is unclear why that figure is referenced here.

7. Sample source definition: I understand that the MetaSRA sample type classifications were used, but it is not clear to me how they were mapped to "biopsy" and "lab grown cell line" categories. It sound like "tissue" was mapped to "biopsy" but I'm not sure about the rest. One MetaSRA category is "primary cells", which can be cells sorted from a disassociated (biopsied) tissue sample. Are those also considered "biopsy?"

8. What was used for "gene length" to normalize to TPM?

9. "Metadata annotation" section: I did not understand the phrase "no samples were discharged because of their tissue label." Are samples being removed from the training or test sets with some criteria?

10. DA model architecture: the text says that the model "is trained on semi-hard triplets" and then gives a definition of this based on Euclidean distances in the embedding spaces. This confuses me because bias embedding mapper (BM) is what is being trained here, so this appears circular. How do you get the distances without already having the BM?

11. Figure 3D: y-axis appears to be mislabeled

Level of Interest

Please indicate how interesting you found the manuscript: Choose an item.

Quality of Written English

Please indicate the quality of language in the manuscript: Choose an item.

Declaration of Competing Interests

Please complete a declaration of competing interests, considering the following questions:

- Have you in the past five years received reimbursements, fees, funding, or salary from an organisation that may in any way gain or lose financially from the publication of this manuscript, either now or in the future?
- Do you hold any stocks or shares in an organisation that may in any way gain or lose financially from the publication of this manuscript, either now or in the future?
- Do you hold or are you currently applying for any patents relating to the content of the manuscript?
- Have you received reimbursements, fees, funding, or salary from an organization that holds or has applied for patents relating to the content of the manuscript?
- Do you have any other financial competing interests?
- Do you have any non-financial competing interests in relation to this paper?

If you can answer no to all of the above, write 'I declare that I have no competing interests' below. If your reply is yes to any, please give details below.

I declare that I have no competing interests

I agree to the open peer review policy of the journal. I understand that my name will be included on my report to the authors and, if the manuscript is accepted for publication, my named report including any attachments I upload will be posted on the website along with the authors' responses. I agree for my report to be made available under an Open Access Creative Commons CC-BY license (http://creativecommons.org/licenses/by/4.0/). I understand that any comments which I do not wish to be included in my named report can be included as confidential comments to the editors, which will not be published.

Choose an item.

To further support our reviewers, we have joined with Publons, where you can gain additional credit to further highlight your hard work (see: https://publons.com/journal/530/gigascience). On publication of

this paper, your review will be automatically added to Publons, you can then choose whether or not to claim your Publons credit. I understand this statement.

Yes Choose an item.
