# Supplementary material for: Bias-invariant RNA-sequencing metadata annotation
Source: Gigascience. 2021 Sep 22;10(9):giab064. doi: 10.1093/gigascience/giab064 (PMC8559615; doi:10.1093/gigascience/giab064)
Supplement: giab064_Reviewer_1_Report_Revision_1 — Colin Dewey -- 7/8/2021 Reviewed [file giab064_reviewer_1_report_revision_1.pdf]

Reviewer Report

Title: Bias invariant RNA-seq metadata annotation

Version: Revision 1 **Date:** 7/8/2021

Reviewer name: Colin Dewey

Reviewer Comments to Author:

With this revised manuscript the authors have addressed the majority of my previous comments. I have a handful of new or remaining comments:

Major comments:

1. Overall claims made in the abstract and a couple of other places in the manuscript that the DA algorithm specifically outperforms the linear regression and traditional neural network models are either misleading or incorrect. What *is* clear is that training on heterogenous (or "multiple bias" in the terminology of the authors) datasets results in improvements when testing on samples from other datasets. I consider this to be the main finding of this work. The authors should rephrase their main findings to make this clear and not overstate the performance of the DA model. In particular, the abstract states:
"We present a deep-learning based domain adaptation algorithm for the automatic annotation of RNA-seq metadata. We show how our algorithm outperforms existing linear regression based approaches as well as traditional neural network methods for the prediction of tissue, sample source, and patient sex information across several large data repositories."
This statement is not supported by the manuscript. For sample source, DA is not evaluated, so it cannot have outperformed the other methods on that task. For patient sex, a traditional neural network trained on heterogenous data outperforms DA on both SRA and TCGA. For tissue, DA does have better accuracy than the other methods for TCGA data. For SRA tissue data, DA is comparable to a traditional neural network trained on heterogenous data.
2. The new experiments that test the neural networks' robustness to mislabeled training data are most welcome. However, I think it is important to note that mislabeling is likely not random, especially if it arises as an error in the MetaSRA's automated pipeline. Unlike random errors, systematic errors create signals in the training data that a model can learn and then replicate on test data. I am not suggesting that the authors perform new experiments (which may be challenging in this case). Rather, I am suggesting that the authors discuss this point.

Minor comments:

3. The definition of how "gene length" is determined is still unclear. The manuscript cites the Gencode annotation as providing gene length, but this is still not clear. Gene length is not a well-defined concept because each isoform of a gene can have a different length, and the isoforms of a gene can be expressed at very different levels. For a given RNA-seq sample, the accepted way to define "gene length" is to use the expression-weighted average of the gene's isoform lengths.
4. In a number of places in the manuscript there are statements about the MetaSRA mislabeling samples. It should be made clear that the MetaSRA automated pipeline serves only to *standardize*

the metadata in SRA (with the exception of sample source, which is predicted). There may be many errors in the raw metadata, and these will simply be standardized by the MetaSRA. Of course, it is also possible that the standardization process is also introducing errors. However, I think it is important to note the various ways in which these errors can arise.
5. The definition of the triplets for training the BM is still a bit unclear. Are these triplets defined *once* at the beginning of training when the BM and SM have the same weights? Or is the triplet set updated during the training procedure as the BM weights change?

Level of Interest

Please indicate how interesting you found the manuscript: Choose an item.

Quality of Written English

Please indicate the quality of language in the manuscript: Choose an item.

Declaration of Competing Interests

Please complete a declaration of competing interests, considering the following questions:

- Have you in the past five years received reimbursements, fees, funding, or salary from an organisation that may in any way gain or lose financially from the publication of this manuscript, either now or in the future?
- Do you hold any stocks or shares in an organisation that may in any way gain or lose financially from the publication of this manuscript, either now or in the future?
- Do you hold or are you currently applying for any patents relating to the content of the manuscript?
- Have you received reimbursements, fees, funding, or salary from an organization that holds or has applied for patents relating to the content of the manuscript?
- Do you have any other financial competing interests?
- Do you have any non-financial competing interests in relation to this paper?

If you can answer no to all of the above, write 'I declare that I have no competing interests' below. If your reply is yes to any, please give details below.

I declare that I have no competing interests.

I agree to the open peer review policy of the journal. I understand that my name will be included on my report to the authors and, if the manuscript is accepted for publication, my named report including any attachments I upload will be posted on the website along with the authors' responses. I agree for my report to be made available under an Open Access Creative Commons CC-BY license

(http://creativecommons.org/licenses/by/4.0/). I understand that any comments which I do not wish to be included in my named report can be included as confidential comments to the editors, which will not be published.

Choose an item.

To further support our reviewers, we have joined with Publons, where you can gain additional credit to further highlight your hard work (see: https://publons.com/journal/530/gigascience). On publication of this paper, your review will be automatically added to Publons, you can then choose whether or not to claim your Publons credit. I understand this statement.

Yes Choose an item.
