# Supplementary material for: Bias-invariant RNA-sequencing metadata annotation
Source: Gigascience. 2021 Sep 22;10(9):giab064. doi: 10.1093/gigascience/giab064 (PMC8559615; doi:10.1093/gigascience/giab064)
Supplement: giab064_Reviewer_2_Report_Original_Submission — Maren BÃ%ttner -- 4/23/2021 Reviewed [file giab064_reviewer_2_report_original_submission.pdf]

Reviewer Report

Title: Bias invariant RNA-seq metadata annotation

Version: Original Submission **Date:** 4/23/2021

Reviewer name: Maren BÃ¼ttner

Reviewer Comments to Author:

Summary:
The authors present a Domain adaptation model the uses a Siamese network architecture to lead missing metadata from bulk RNAseq data and compare the performance to a previously published linear regression model (LIN) and a multilayer perceptron (MLP). As data sources, the authors used GTEx, SRA, TCGA. The DA model outperforms the LIN and MLP, when many classes to learn (e.g. in case of tissues), but not in the case of sex and sample source. While the authors present their work in a concise and clear way, I think that they can improve their manuscript in several points.

Major:
I did not see a cross-validation of any of the used models. Instead, the authors varied the random seeds for model initialization. While I appreciate the split by study in the case of the SRA data, I think that the authors should add a cross-validation approach on the model training to increase robustness, even if that means that the training dataset has varying size.

Page 8: The introduction of the "percentage point" (ppt) as metric is superfluous. I suggest to use percent (%) instead, if the authors really want to state relative changes. Further, I have the impression that ppt and % are used synonymously, so the authors should use the unit consistently.

Figure 3:
Plotting the relative change to the baseline model overrates the actual improvement of the DA approach and is not statistically sound. I suggest to state absolute changes in accuracy (or mca/msa) instead.

Statistical analysis page 8: When using a t-test, the authors assume that the msa/mca scores would be normally distributed around some unknown mean value. By looking at the boxplots, I think that the assumption is not necessarily true and I suggest to use a non-parametric test (e.g. Wilcoxon rank sum test) instead of the t-test.

Identifying novel training data (page 10): I would be very careful in including predicted labels as ground truth data (which is known as data imputation task). In these cases, retraining a classifier on both ground truth and predictions will lead to overfitting and spurious results, when the predicted labels dominate the ground truth labels.

Minor:
Page 4: Gene selection based on Gini index: was there an overlap of the genes used for tissue and sample source classification? I would assume so from the setup with the range of Gini indices.

Figures and Subfigures are not fully in the order of first appearance (esp. subfigures 1 B-C compared to figure 2).

Figure 1A: Datasets could be visualized as bar charts to give the reader an idea about the dataset sizes. Exact numbers can be stated in the supplement or figure legend

Figure 1: The figure design is clean, however, the green TCGA box is not colorblind friendly and difficult to distinguish from the purple GTEx box. Consider a lighter color.
Figure 3:
I struggle to understand the plot, especially the numbers at the top of each boxplot (please clarify in the figure legend, that this is msa and mca).
What was the baseline model in Figure 3D? Why do you present this as main figure, when the relative changes are within 1% (and therefore within the range of the noise level)?
Supplementary Figure 7A:
The PCA analysis of the ovary data is interesting. The differences of TCGA-ovary and GTEx-ovary data is reflected in PC1 and I was wondering whether the authors could give a more detailed explanation on the reasons for this systematic bias, e.g. by analysing the loadings of PC1.
Page 9: Clarify. "For example, SRP056612 is a study on the effect of the coronavirus on cultured kidney and
lung cells [39] and SRP045611 is a study involving HEK cells, which lack the Y chromosome but are annotated as male by MetaSRA [40]."
As far as I understood the cited reference, it corresponds to the MERS coronavirus. Second, HEK cells are (most likely) of female origin, therefore, clearly state the nature of the mislabeling (I consider this as human error in the MetaSRA).
By the way, line numbers would have been nice to comment on certain passages.

Level of Interest

Please indicate how interesting you found the manuscript: Choose an item.

Quality of Written English

Please indicate the quality of language in the manuscript: Choose an item.

Declaration of Competing Interests

Please complete a declaration of competing interests, considering the following questions:

- Have you in the past five years received reimbursements, fees, funding, or salary from an organisation that may in any way gain or lose financially from the publication of this manuscript, either now or in the future?
- Do you hold any stocks or shares in an organisation that may in any way gain or lose financially from the publication of this manuscript, either now or in the future?
- Do you hold or are you currently applying for any patents relating to the content of the manuscript?
- Have you received reimbursements, fees, funding, or salary from an organization that holds or has applied for patents relating to the content of the manuscript?

- Do you have any other financial competing interests?
- Do you have any non-financial competing interests in relation to this paper?

If you can answer no to all of the above, write 'I declare that I have no competing interests' below. If your reply is yes to any, please give details below.

I declare that I have no competing interests

I agree to the open peer review policy of the journal. I understand that my name will be included on my report to the authors and, if the manuscript is accepted for publication, my named report including any attachments I upload will be posted on the website along with the authors' responses. I agree for my report to be made available under an Open Access Creative Commons CC-BY license (http://creativecommons.org/licenses/by/4.0/). I understand that any comments which I do not wish to be included in my named report can be included as confidential comments to the editors, which will not be published.

Choose an item.

To further support our reviewers, we have joined with Publons, where you can gain additional credit to further highlight your hard work (see: https://publons.com/journal/530/gigascience). On publication of this paper, your review will be automatically added to Publons, you can then choose whether or not to claim your Publons credit. I understand this statement.

Yes Choose an item.
